# A novel family of (1-aminoalkyl)(trifluoromethyl)- and -(difluoromethyl)phosphinic acids – analogues of α-amino acids

**DOI:** 10.3762/bjoc.10.66

**Published:** 2014-03-26

**Authors:** Natalia V Pavlenko, Tatiana I Oos, Yurii L Yagupolskii, Igor I Gerus, Uwe Doeller, Lothar Willms

**Affiliations:** 1 Institute of Organic Chemistry National Academy of Sciences of Ukraine, Murmanskaya str. 5, 02660 Kiev-94, Ukraine; 2 Institute of Bioorganic Chemistry and Petrochemistry National Academy of Sciences of Ukraine, Murmanskaya str. 1, 02660 Kiev-94, Ukraine; 3 Bayer CropScience Aktiengesellschaft BCS AG-R-WC-WCC-C2 Weed Control Chemistry 2, Frankfurt, G836, 101, Germany

**Keywords:** (1-aminoalkyl)phosphinic acids, ethyl (difluoromethyl)phosphinate, hydrophosphinylation, organo-fluorine, Shiff bases, (trifluoromethyl)phosphinic acid

## Abstract

A series of novel (1-aminoalkyl)(trifluoromethyl)- and -(difluoromethyl)phosphinic acids – analogues of proteinogenic and nonproteinogenic α-amino acids were prepared. The synthetic methodology was based on nucleophilic addition of (trifluoromethyl)phosphinic acid or (difluoromethyl)phosphinic acid or its ethyl ester to substrates with C=N or activated C=C double bonds. Analogues of glycine, phenylglycine, alanine, valine, proline, aminomalonic and aspartic acids were thus prepared. Three-component one-pot reactions of (trifluoromethyl)phosphinic acid and dibenzylamine with aldehydes were also tested to prepare the title compounds.

## Introduction

For a long time aminophosphonic and aminophosphinic acids as isosters of aminocarboxylic acids have attracted a particular interest for the preparation of analogues of numerous natural products. Among the literature concerning various aspects of the chemistry and biological activity of aminophosphonic and aminophosphinic acids, several monographs and reviews have appeared over the last decade [1–6]. The chemistry of fluorinated aminophosphonic and -phosphinic acids is a relatively new area of research. Incorporation of fluorine or fluorinated moieties can be used for the alteration of physiological properties of many biologically significant substances. The changes of their biological properties caused by this fluorination are influenced by complex factors, however. The similarity of the diameters of fluorine and hydrogen atoms in organic compounds makes fluorine an obvious choice as a substituent for biologically active substances, frequently without disrupting the shape and geometry of the substituted molecules. Nevertheless fluorine influences the electronic properties of a compound drastically because of its strong electronegativity. This enables modulation of the lipophilicity profile, of electrostatic interactions with the target structure and inhibition of some metabolic pathways [7–9]. Data concerning the biological activity and synthetic approaches toward fluorinated aminophosphonates, bearing side chain C–F linkages are well documented in a review [10].

The isolation of phosphinothricin, a naturally occurring phosphorus analogue of glutamic acid and the discovery of its antibiotic, fungicidal and herbicidal properties [11] has led to an increased activity in the study of methylphosphinic acid analogues of the protein amino acids [12] and those of glycine [13], alanine [14], valine [14], leucine [15], proline [16], aspartic [17] and glutamic [11] acids and GABA [18] have been described. But almost nothing is known about phosphorus isosters of aminocarboxylic acids bearing a (trifluoromethyl)- or (difluoromethyl)phosphonyl moiety instead of the carboxylate function. To the best of our knowledge there is only one report on the application of ethyl (difluoromethyl)phosphinate CHF_2_(H)P(O)(OEt) in the synthesis of a (difluoromethyl)phosphinic acid analogue of GABA, as a potent agonist of the GABA_B_ receptor [18].

In light of the above and in connection with our interest in the chemistry of fluorinated compounds of phosphorus we report here the preparation of a series of novel (1-aminoalkyl)phosphinic acids bearing CF_3_ or CHF_2_ groups at phosphorus.

## Results and Discussion

Research efforts have established H-phosphinates R(H)P(O)(OR′) or appropriate P(III) acids R(H)P(O)(OH) as appropriate starting materials for the preparation of aminophosphinic acids. The most typical route involves the three-component reaction of an aldehyde, an amine and a P–H substrate in a one-pot Mannich type protocol [19–21]. An alternative to this approach involves the simple addition of alkyl H-phosphinates or H-phosphinic acids to Schiff bases [5,22]. In this paper we exploit both routes to prepare (1-aminoalkyl)(trifluoromethyl)- and -(difluoromethyl)phosphinic acids using P–H compounds bearing CF_3_ and CHF_2_ groups attached to phosphorus.

### P–H Substrates

**Scheme 1:**
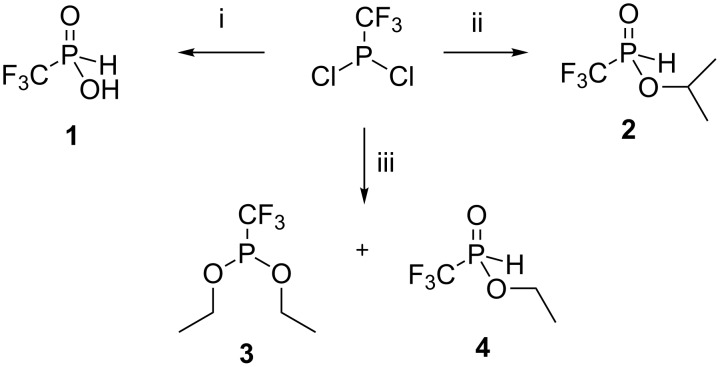
Synthesis of (trifluoromethyl)phosphinic acid (**1**) and ethyl and isopropyl esters **2–4**. Reagents and conditions: i) 1.8 equiv H_2_O per 1 equiv CF_3_PCl_2_, hexane, −10 °C → 0 °C, 3 h, then rt overnight, argon atmosphere, 84%. ii) 2 equiv anhydrous iPrOH per 1 equiv CF_3_PCl_2_, −40 °C → 0 °C, 3 h, then rt overnight, argon atmosphere, 68%. iii) 2 equiv anhydrous EtOH per 1 equiv CF_3_PCl_2_, −40 °C → 0 °C, 3 h, then rt overnight, argon atmosphere, mixture **3**:**4** ~ 10:1, ~70%.

(Trifluoromethyl)phosphinic acid CF_3_P(O)H(OH) (**1**) was first prepared in 1954 [23], but since then little chemistry has been reported involving **1**. Also monoesters of **1** such as CF_3_P(O)H(OAlk) [24–25] have not been widely applied. These compounds, contain a labile P–H bond and synthetic problems underly their preparation. Emeléus and Haszeldine were the first [26] to prepare CF_3_P(III) compounds via the interaction of red phosphorus and CF_3_I in an autoclave. This gave mixtures of CF_3_-containing phosphanes and phosphane iodides but in poor yields. More recently, Ruppert described the reaction between CF_3_Br, P(NEt_2_)_3_ and PCl_3_, which gave CF_3_P(NEt_2_)_2_ in a good yield [27]. We applied this procedure to prepare CF_3_PCl_2_ [27] by interaction of diamide CF_3_P(NEt_2_)_2_ with gaseous HCl and then the chlorine was replaced by neutral hydrolysis to give (trifluoromethyl)phosphinic acid (**1**) [23] or by alcoholysis with ethanol or isopropanol to reach the appropriate esters **2–4** [24–25] (Scheme 1).

In our hands CF_3_PCl_2_ was hydrolyzed by two equivalents of water in hexane over the temperature range −10 °C → 0 °C, to give water-free (trifluoromethyl)phosphinic acid (**1**). Acid **1** proved easy to handle as a distillable liquid when prepared in this way and is stable under storage for months in contrast to that prepared by Emeléus and Haszeldine [23]. Phosphinate **2** was prepared with anhydrous isopropanol. When stored under anhydrous conditions at room temperature, ester **2** was partially converted to acid **1** as determined by ^31^P NMR. Alcoholysis of CF_3_PCl_2_ with ethanol under the same conditions produced diethyl phosphinate **3** admixed with monoester **4** (~10%), as previously described [25]. ^31^P NMR of these products indicated that they were converted to esters **3** and **4** on storage in a ratio of ~ 3:2 with acid **1** as an impurity. The low stability of all three esters **2**–**4** can be attributed to the lability of the O–C ester bond, resulting from the electron-withdrawing effect of the CF_3_ group attached to phosphorus, therefore esters **2**, **3** and **4** were used in the syntheses only when freshly prepared and distilled.

We next explored the CHF_2_ group attached to phosphorus. Ethyl (difluoromethyl)phosphinate CHF_2_P(O)H(OEt) (**5**) was prepared as previously described [18]. The appropriate (difluoromethyl)phosphinic acid CHF_2_P(O)H(OH) (**6**) was obtained from **5** by ester deprotection with NaHCO_3_, as a viscous undistillable liquid, which was stable for weeks on storage.

### Three-component reactions

**Scheme 2:**
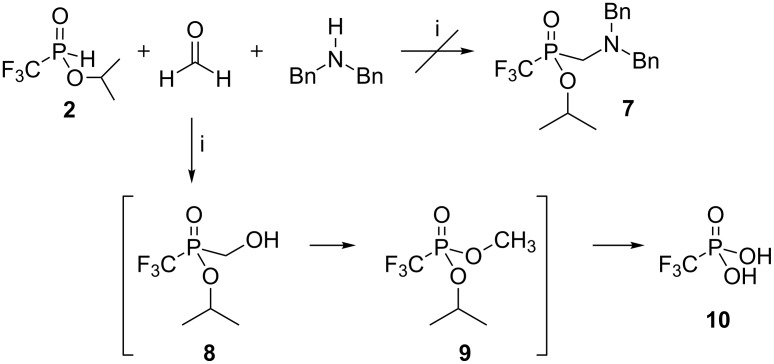
Three-component Kabachnik–Fields reaction of CF_3_(H)P(O)(OiPr) (**2**) with formaldehyde and dibenzylamine. Reagents and conditions: i) an equimolar mixture of reagents, H_2_O, 80 °C, 3 h, argon atmosphere, yield **10** ~80% or an equimolar mixture of reagents, dioxane, 100 °C, 3 h, argon atmosphere, yield **10** ~90%.

At the outset of our work three-component reactions of formaldehyde, dibenzylamine and the esters **2** or **5** were explored as model transformations to evaluate the feasibility of the Kabachnik–Fields procedure [19–20] to the synthesis of fluorinated (1-aminoalkyl)phosphinate **7** (Scheme 2).

It turned out that this method is unsuitable for the synthesis of phosphinate **7**. Formalin was added to an equimolar mixture of dibenzylamine and ester **2** at 80 °C under an oxygen-free atmosphere to give reaction mixtures with a low content of P–C products (^31^P NMR). A similar outcome was obtained when the reaction was run at room temperature or in dioxane with simultaneous water azeotropic distillation. Such an result might be explained by high reactivity of the starting ester, which readily reacted with formaldehyde, forming (α-hydroxymethyl)phosphinate **8**. Its further irreversible rearrangement [28] to the corresponding phosphonate **9** was accompanied with hydrolysis of the ester function and formation of (trifluoromethyl)phosphonic acid (**10**) [29] as the main product. Analogous results were obtained, when CHF_2_ containing ester **5** was introduced into the reaction with formaline and dibenzylamine to give CHF_2_P(O)(OH)_2_ (**11**) as the major product [30].

**Scheme 3:**
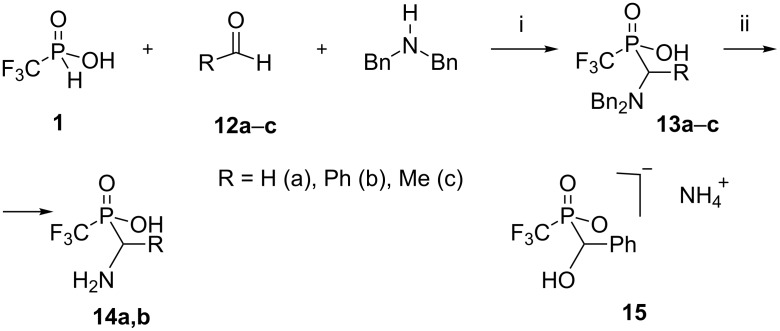
Three-component synthesis of CF_3_ containing α-aminophosphinic acids **14a,b**. Reagents and conditions: i) An equimolar mixture of acid **1**, dibenzylamine, HCl and two fold excess of aldehyde, H_2_O, 80 °C, 3 h; isolated yields: **13a** (52%), **13b** (28%); yields, determined by ^31^P and ^19^F NMR: **13c** (<10%). ii) H_2_, ethanol, catalysis 10% Pd/C, rt, normal pressure, yields: **14a** (95%), **14b** (90%).

Such results prompted us to explore the Mannich-type procedure of Moedritzer and Irani [21] for the syntheses of the desired aminophosphinic acids starting from acid **1**. This resulted in the preparation of the analogues of glycine **14a** and phenylglycine **14b** (Scheme 3).

The three-component reaction with formaldehyde gave the best results and *N-*protected aminophosphinic acid **13a** was isolated in a moderate yield, alongside phosphonic acid **10**. The analogous reaction with benzaldehyde provided acid **13b** in 28% yield and the main product of this reaction was an adduct of acid **1** with benzaldehyde which was isolated from the reaction mixture as ammonium salt **15** in 60% yield. Reaction with acetaldehyde was less successful and generated aminophosphinic acid **13c** in low yield (<10%). Attempts to improve conversions products **13a–c** by increasing the reaction temperature or varying the amino component (MeC(O)NH_2_, BnOC(O)NH_2_ or NH_4_OAc instead of Bn_2_NH) and molar equivalent of HCl were unsuccessful. It should be noted, that in contrast to the non-fluorinated counterparts the adducts **13a,b** did not form hydrochlorides under this procedure consistent with the strongly acidic nature of the CF_3_ phosphinic acid group. Catalytic hydrogenation of intermediates **13a,b** with Pd/C removed the benzyl groups and produced the corresponding acids **14a,b** in high yields.

### The hydrophosphinylation of azomethines

The addition of the P–H functionality to C=N double bonds is a very general procedure for the formation of P–C–N systems. Based on our experience of these three-component reactions (Scheme 2 and Scheme 3) we investigated the scope and limitations of the addition of (trifluoromethyl)phosphinic acid (**1**) to a series of *N*-benzylimines **16a**–**e** in order to obtain fluorinated phosphorus analogues of glycine **14a**, phenylglycine **14b**, alanine **14c**, valine **14d** and proline **14e** (Table 1).

**Table 1:** The interaction of (trifluoromethyl)phosphinic acid (**1**) with Shiff bases.^a^

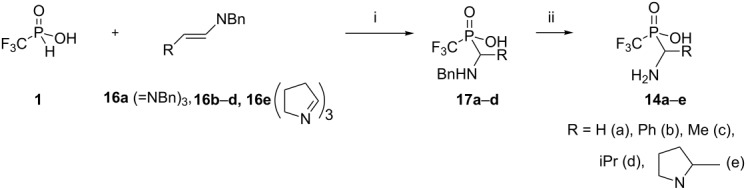				

Entry	Shiff base	R	**17** (yield, %)^b^	**14** (yield, %)^b^

1^c^	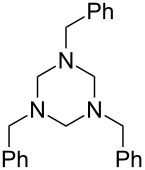	H	**17a** (83)	**14a** (95)
2	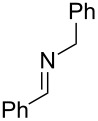	Ph	**17b** (79)	**14b** (96)
3^d^	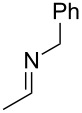	Me	**17c** (59)	**14c** (96)
4	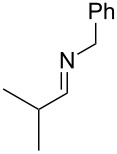	iPr	**17d** (92)	**14d** (98)
5^c^	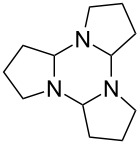	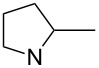	–	**14e** (66)

^a^Reagents and conditions: i) an equimolar mixture of acid **1** and Shiff base, DME, rt, ^31^P NMR control; ii) H_2_, ethanol, catalysis 10% Pd/C, rt, normal pressure. ^b^Isolated yields. ^c^Symmetrical cyclic triazinanes (masked imines) were used to generate unstable imines. ^d^The best yield was obtained with 2 mol equivalents of imine.

The transformations were mildly exothermic and were monitored by ^31^P NMR. Acid **1** undergoes the typical P–C bond forming reactions with Shiff bases to give adducts **17** in satisfactory yields and these were successfully transformed into the appropriate free acids **14**.

The same series of Shiff bases was used to explore the reactivity of ethyl (difluoromethyl)phosphinate (**5**) in reactions with C=N double bonds and the desired (α-aminoalkyl)phosphinic acids **20a–e** were accordingly prepared (Table 2).

**Table 2:** The interaction of ethyl (difluoromethyl)phosphinate (**5**) with Shiff bases.^a^

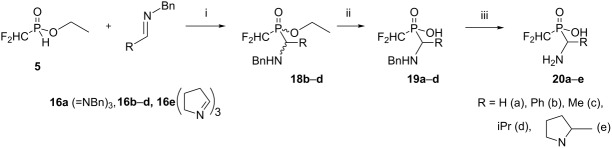					

Entry	Shiff base	R	**18** (yield, %)^b^	**19** (yield, %)^b^	**20** (yield, %)^b^

1^c^	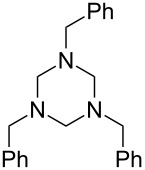	H	**18a** (78)^d^	**19a** (68)	**20a** (91)
2	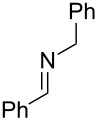	Ph	**18b** (58)^e^	**19b** (86)^f^	**20b** (96)
3^g^	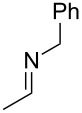	Me	**18c** (56)^e^	**19c** (80)^f^	**20c** (95)
4	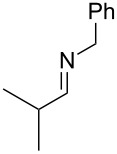	iPr	**18d** (36)^e^	**19d** (82)^f^	**20d** (95)
5^c^	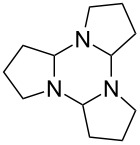	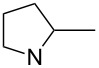	–	–	**20e** (79)

^a^Reagents and conditions: i) an equimolar mixture of ester **5** and Shiff base, DME, rt, ^31^P NMR control, under argon atmosphere; ii) 1 N HCl, rt, until clear solution; iii) H_2_, ethanol, catalysis 10% Pd/C, rt, normal pressure. ^b^Isolated yields. ^c^Symmetrical cyclic triazinanes (masked imines) were used to generate unstable imines. ^d^Yield was defined with ^31^P NMR. ^e^Diastereomeric ratio: **18b** (~7:2), **18c** (~3:2), **18d** (~3:2). ^f^Yields were defined as the sum of yields of compounds **19b–d**, isolated from the reaction mixture and obtained after hydrolysis of intermediates **18b–d**. ^g^The maximum yield was obtained with 2 mol equivalents of imine.

The syntheses of compounds **20a–e** were performed with purification and characterization of the intermediates, wherever possible as summarized in Table 2.

Products **14b–e** and **20b–e** were obtained as racemic mixtures. As expected, the ^31^P NMR spectra of **14** and **20** display characteristic signals around 11–15 ppm with ^2^*J*_FP_ couplings for CF_3_ bearing substrates. The CHF_2_ bearing also possessed ^2^*J*_FP_ couplings of 75–95 Hz. The ^19^F NMR spectra were characterized by signals in the region −75 ppm for CF_3_ derivatives and −137 ppm for CHF_2_ ones. Some distinctive characteristics of the reactivity of ester **5** were observed. This ester readily reacted with azomethines, but in contrast to its nonfluorinated counterparts this ester generated mixtures of the adducts **18** and **19**. The interaction of ester **5** with imine **16a** gave adduct **18a** (Table 2, entry 1) in high conversion yield but after purification over silica gel only the appropriate acid **19a** was isolated. In the case of the reaction of ester **5** with imine **16e** no adduct was formed. Ethyl phosphinates **18b–d** were obtained as a mixture of two diastereoisomers, which were not separated but they are clearly observed by ^1^H NMR as separate signals for the CHF_2_ group. The adducts **18b–d** were hydrolyzed to acids **19b–d** in quantitative yields and did not form hydrochlorides similar to the CF_3_ aminophosphinic acids **13a–b** and **17a–d**. Hydrogenolysis of the **17a–d** and **19a–d** efficiently gave free acids **14** and **20**, but required column ion-exchange chromatography to produce analytically pure products.

**Scheme 4:**
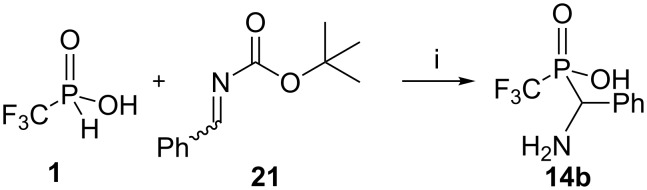
Interaction of the acid **1** with *tert*-butyl benzylidenecarbamate (**21**). Reagents and conditions: i) an equimolar mixture of reagents, DME, rt, 48 h, under argon atmosphere, 32%.

We then explored the addition of acid **1** to imine **21** [31], which is *N-*Boc protected, typically used for amino acid protection (Scheme 4). This produced acid **14b** in one step, but in only 32% yield. The *N-tert*-butoxycarbonyl group was removed during the reaction due to the high acidity of the CF_3_ phosphinic acid group.

**Scheme 5:**
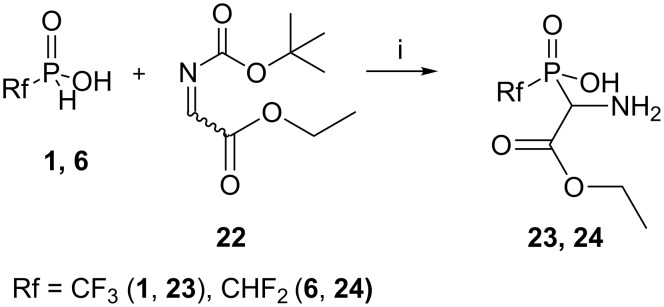
Interaction of the acids **1** and **6** with ethyl 2-[(*tert*-butoxycarbonyl)imino]acetate (**22**). Reagents and conditions: i) an equimolar mixture of reagents, DME, rt, ^31^P NMR control, under argon atmosphere, isolated yields: **23** (68%), **24** (61%).

The variability of Schiff bases ensures access to a range of structurally diverse phosphinic acid analogues of amino acids in the relatively simple way. Thus, we investigated the hydrophosphinylation of some Shiff bases bearing a carboxylate functionality to obtain aminocarboxylic acids, containing pendant CF_3_ or CHF_2_ phosphinic acid linkages. Thus, phosphinic acids **1** and **6** reacted with the *N-*Boc*-*protected Schiff base of ethyl glyoxalate **22** [32] under mild conditions to produce the *N-*deprotected phosphinylglycines **23** and **24** in satisfactory yields (Scheme 5).

Attempts to convert ester **23** to the free acid failed. Removal of the ester group from **23** by acidolysis with HCl or HI was accompanied by cleavage of the P–C bond to give only (trifluoromethyl)phosphonic acid (**10**) after an ion-exchange chromatography. Attempts to remove the ester group in anhydrous base with 1 equivalent of sodium silanolate (Me_3_SiONa) at room temperature efficiently produced the highly stable sodium salt of acid **23**. With an excess of Me_3_SiONa and heating to 50 °C, fluoroform liberation from **23** was observed to give fluorine-free products.

**Scheme 6:**
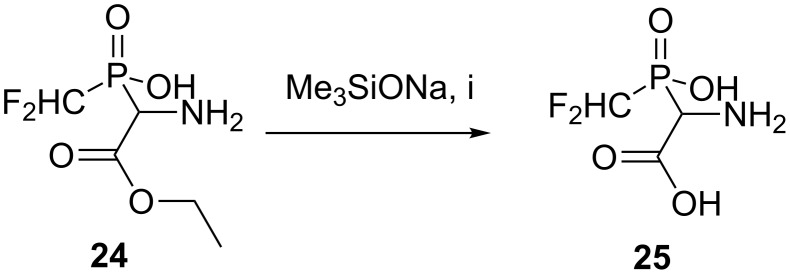
Transformation of the ester **24** into the appropriate free acid **25**. Reagents and conditions: i) two fold excess of Me_3_SiONa, DME, 50 °C, 24 h, under argon atmosphere, ion-exchange chromatography, H_2_O, 35%.

In contrast the CHF_2_-containing ester **24** was stable toward acidic hydrolysis under mild conditions, but in the presence of an excess of sodium silanolate, free phosphinylglycine **25** was obtained, but in a poor yield (Scheme 6).

**Scheme 7:**
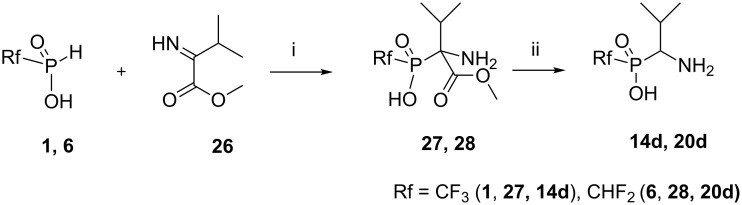
Reaction of the acids (**1**) and (**6**) with methyl 2-imino-3-methylbutanoate (**26**). Reagents and conditions: i) an equimolar mixture of reagents, DME, rt, ^31^P NMR control, under argon atmosphere, isolated yields: **27** (63%), **28** (40%).

The reactions of substrates **1** and **6** with imine **26**, which is available from valine [33], readily gave adducts **27** and **28**, which were decarboxylated under acidolysis to afford the phosphinic acid analogues of valine **14d** and **20d** (Scheme 7).

### The hydrophosphinylation of substrates with activated C=C double bonds

**Scheme 8:**
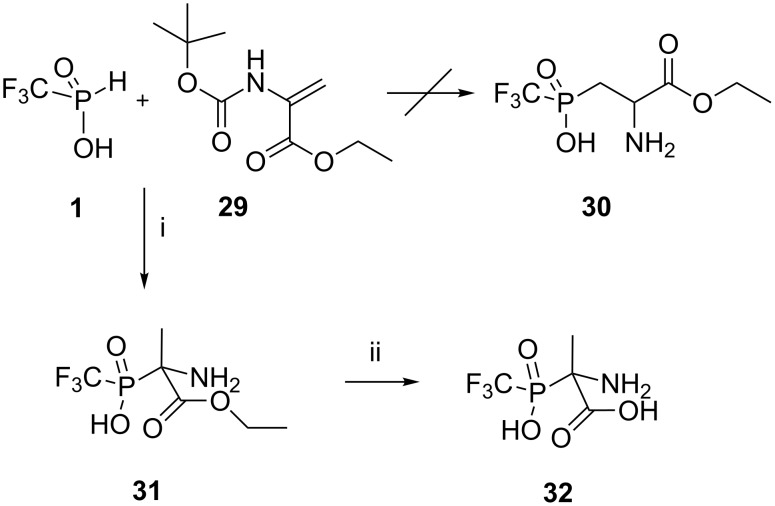
Interaction of the acid **1** with ethyl 2-(*tert*-butoxycarbonylamino)acrylate (**29**). Reagents and conditions: i) an equimolar mixture of reagents, DME, rt, ^31^P NMR control, under argon atmosphere, 59%. ii) 5 N HCl, rt, 48 h, ion-exchange chromatography, H_2_O, 54%.

The high reactivity of (trifluoromethyl)phosphinic acid (**1**) with C=N double bonds prompted us to explore its reactivity towards activated C=C double bonds. By analogy with the synthesis of the phosphonic acid analogue of aspartic acid, developed by Chambers and Isbell [34], the P–H substrate **1** was reacted with *N-*Boc*-*protected aminoacrylate **29** [35], and this gave the precursor of the aspartic acid analogue **30** (Scheme 8).

Surprisingly, under the mild conditions of our experiment only the addition of acid **1** to the C=N double bond of the acrylic ester **29**, occurred to produce the *tertiary* phosphinyl derivative of alanine **31** in a satisfactory yield. Ester **31** was then hydrolyzed to give the free acid **32** in a moderate yield.

**Scheme 9:**
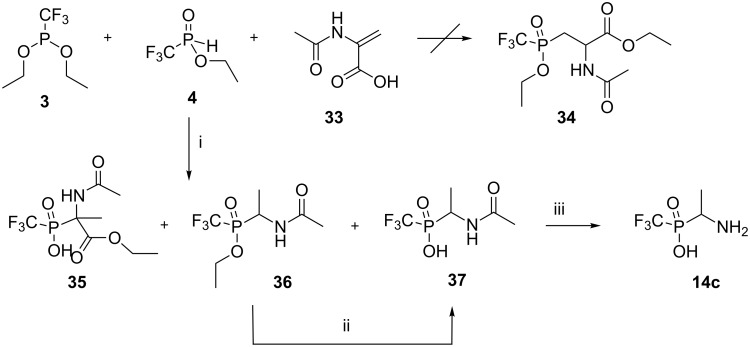
Interaction of a mixture of the esters **3** and **4** with 2-acetamidoacrylic acid (**33**). Reagents and conditions: i) an equimolar mixture of **4** and **33** and 1.5 equiv of **3**, rt, ^31^P NMR control, under argon atmosphere, isolated yields: **35** (7%), **36** (40%) (diastereomeric ratio ~8:7), **37** (18%). ii) 5 N HCl, rt, 24 h, 95%. iii) 5 N HCl, in an ampoule, 130 °C, 8 h, 34%.

For the synthesis of the aspartic acid analogue **34**, a reaction between a mixture of the freshly prepared esters **3** and **4** and *N*-acetyl-protected aminoacrylic acid **33** was carried out [34] (Scheme 9).

It was thought that diester **3** might esterify amidoacrylic acid **33** [17,34] to produce ethyl 2-acetamidoacrylate and this compound in turn might add to monoester **4** to give the protected phosphinic acid analogue of aspartic acid **34**. Unfortunately, the only addition of **4** to the C=N double bond occurred similar to the previous transformation illustrated in Scheme 8. Insoluble in the reaction mixture phosphinic acid **37** was filtered off and characterized. ^31^P NMR analysis of filtrate showed the presence of adduct **35** and the decarboxylation product **36** in an approximate 1:10 ratio along with starting esters **3** and **4** and (trifluoromethyl)phosphonic acid (**10**) (<5%). Products **35** and **36** were separated by chromatography and characterized. Ester **36** was obtained as a mixture of two diastereoisomers, which are clearly seen by ^1^H- and ^19^F NMR. This ester was then readily converted by acidolysis into the phosphinic acid analogue **37**, of *N*-acetylalanine, which was isolated in the 56% from acid **33**, and then transformed into free amino acid **14c**.

**Scheme 10:**
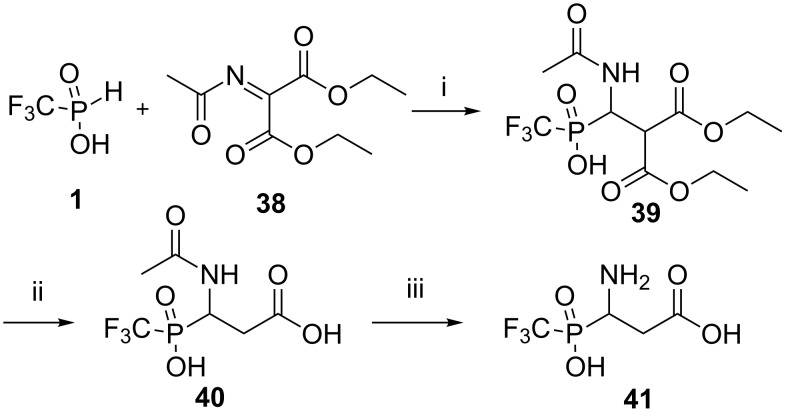
Interaction of a mixture of the acid **1** with diethyl acetaminomethylenemalonate (**38**). Reagents and conditions: i) an equimolar mixture of reagents, acetonitrile, rt, ^31^P NMR control, under argon atmosphere, 44%. ii) 5 N HCl, reflux, 12 h, 82%. iii) 5 N HCl, in an ampoule, 130 °C, 5 h, 43%.

We have been able to prepare the isomeric aspartic acid analogue **41** with phosphorous α- to the amino group by the analogy with the published method [17,36]. The synthesis of phosphinic acid **41** was accomplished by addition of acid **1** to the activated C=C double bond of malonate **38** followed by hydrolysis and decarboxylation to generate adduct **39** in two steps (Scheme 10).

## Conclusion

In conclusion, we have presented a variety of approaches to novel fluorinated (1-aminoalkyl)phosphinic acids starting from the appropriate fluorinated P–H compounds with CF_3_ or CHF_2_ groups attached to phosphorus. Three-component one pot Mannich-type reactions of CF_3_(H)P(O)(OH) with dibenzylamine and aldehydes were investigated. Also nucleophilic addition of CF_3_(H)P(O)(OH) or CHF_2_(H)P(O)(OEt) to Shiff bases, aminoacrylates and acetaminomethylenemalonate have been used to prepare the title compounds.

## Experimental

All reactions with P–H compounds were performed under an argon atmosphere. Flash chromatography was carried out using Merck silica gel 60 (230–400 mesh ASTM) and Aldrich ion-exchange resin Dowex WX-50. The NMR spectra were recorded on Varian VXR-300 or Bruker Avance DRX-500 spectrometers for ^1^H (TMS); on a Bruker Avance DRX-500 spectrometer for ^13^C {H} (TMS); on Varian Gemini-200 or Varian VXR-300 spectrometers for ^19^F (CFCl_3_) and for ^31^P (H_3_PO_4_).

### Synthesis of starting materials

(Trifluoromethyl)phosphinic acid (**1**). To an emulsion of water (3.2 g, 180 mmol) in anhydrous hexane (20 mL), cooled to −78 °C CF_3_PCl_2_ [27] (16.5 g, 96.5 mmol) was added under stirring and the temperature was slowly raised to −10 °C, when hydrolysis started. The reaction mixture was allowed to come to 0 °C at such a rate to avoid a vigorous reaction (~3 h) and then to room temperature and stirring was continued overnight. Hexane was evaporated under reduced pressure and the residue was distilled to give **1** as a colorless liquid (10.84 g, 84%), bp 35 °C (0.05 mm Hg); ^1^H NMR (300 MHz, DMSO-*d*_6_) δ_H_ 7.05 (dq, ^1^*J*_HP_ = 638.9 Hz, 1H, ^3^*J*_HF_ = 4.2 Hz, P*H*), 13.6 (1H, s, O*H*); ^31^P NMR (121 MHz) δ_P_ 6.1 (dq, ^1^*J*_PH_ = 639 Hz, ^2^*J*_PF_ = 82 Hz); ^19^F NMR (188 MHz) δ_F_ −76.8 (dd, ^2^*J*_FP_ = 82 MHz, ^3^*J*_FH_ = 4 Hz). **Caution**: Safety precautions are necessary, because CF_3_PCl_2_ reacts violently with air. Care must be taken not to warm the reaction system rapidly, because rapid volatilization of gaseous HCl will be accompanied by carrying off CF_3_PCl_2_, which can inflame.

(Difluoromethyl)phosphinic acid (**6**). A mixture of **5** [18] (8 g, 56 mmol) and NaHCO_3_ (7 g, 83 mmol) in ether (50 mL) was stirred overnight at room temperature to produce a bulky precipitate of CHF_2_P(O)H(O)^−^Na^+^ [^31^P NMR (121 MHz, H_2_O): δ_P_ 11.9 (dtd, ^1^*J*_PH_ = 570 Hz, ^2^*J*_PF_ = 87 Hz, ^2^*J*_PH_ = 25 Hz]. This precipitate was filtered, thoroughly washed with ether, solved in water (25 mL) and passed down an ion-exchange column. Water from the resulting solution was evaporated under reduced pressure and the residue was kept in vacuo (0.05 mmHg) for 24 h at room temperature to give **6** as a viscous colorless undistillable liquid (7.19 g, 78%); Anal. calcd for CH_3_F_2_O_2_P: C, 10.35; H, 2.61; P, 26.71; found: C, 10.48; H, 2.70; P, 26.59; ^1^H NMR (300 MHz, DMSO-*d*_6_) δ_H_ 6.15 (tdd, ^2^*J*_HF_ = 48.6 Hz, ^2^*J*_HP_ = 24.5 Hz, ^3^*J*_HH_ = 1.5 Hz, 1H, C*H*F_2_), 6.9 (dm, ^1^*J*_HP_ = 566.5 Hz, 1H, P*H*), 12.7 (s, 1H, O*H*); ^31^P NMR (121 MHz) δ_P_ 12.1 (dtd, ^1^*J*_PH_ = 566 Hz, ^2^*J*_PF_ = 86 Hz, ^2^*J*_PH_ = 25 Hz); ^19^F NMR (188 MHz) δ_F_ 9.6 (dd, ^2^*J*_FP_ = 86 Hz, ^2^*J*_FH_ = 49 Hz ).

### Three-component reactions

**The general procedure for the condensation of the acid 1 with dibenzylamine and aldehydes (I).** An equimolar mixture of **1** (2.68 g, 20 mmol) and dibenzylamine (3.94 g, 20 mmol) in 1 N HCl (20 mL) was heated at 80 °C under stirring. In the course of ~1 h aldehydes **12a,b** were added with a syringe and the reaction mixture was kept at this temperature for additional 1 h. The resulting mixture was left overnight at room temperature to produce the precipitate, which was filtered, washed with acetone–water (10:1) and dried to afford **13a** or **13b**. The filtrate was evaporated to the dryness, the residue was triturated with acetone–water (10:1) to give an additional quantity of **13a,b**.

[(Dibenzylamino)methyl](trifluoromethyl)phosphinic acid (**13a**). Following the general procedure (I) using 3.2 mL of 37% aqueous formaldehyde solution (20 mmol) **13a** was obtained as a white solid (3.57 g, 52%), mp 229 °C; Anal. calcd for C_16_H_17_F_3_NO_2_P: C, 55.98; H, 4.99; N, 4.08; found: C, 55.69; H, 5.28; N. 4.15; ^1^H NMR (300 MHz, DMSO-*d*_6_) δ_H_ 2.94 (d, ^2^*J*_HP_ = 9.3 Hz, 2H, C*H*_2_P), 4.45 (s, 4H, C*H*_2_Ph), 7.47–7.59 (m, 10H, *H*_arom._); ^31^P NMR (121 MHz) δ_P_ 3.8 (qt, ^2^*J*_PF_ = 81 Hz, ^2^*J*_PH_ = 9 Hz); ^19^F NMR (188 MHz) δ_F_ −73.8 (d, ^2^*J*_FP_ = 81 Hz).

**The general procedure for* N*-deprotection of compounds with *N*-Bn function under the catalytic hydrogenation conditions (II).** To a solution of compounds, containing *N-*Bn fragment (5 mmol) in ethanol (10 mL) 10% Pd/C (0.05 g) was added, and the mixture was hydrogenated at room temperature and normal pressure. After ~3 h the precipitation commenced, and water (5 mL) was added to dissolve this precipitate. The hydrogenation was then continued with a fresh portion of the catalyst (0.05 g) for a further 3 h. Last procedure was repeated whenever necessary and the reaction was left overnight. To the resulting mixture water was added until a white solid was fully dissolved, and the catalyst was then filtered off. The filtrate was evaporated to dryness; the residue was dissolved in acetone and allowed to stand at 5 °C until complete precipitation. The formed solid was filtered, washed with acetone and dried to give compounds with the free NH_2_ function.

(Aminomethyl)(trifluoromethyl)phosphinic acid (**14a**). Following the general procedure (II) **14a** was obtained as a white powder (0.78 g, 95%); mp 192 °C; Anal. calcd for C_2_H_5_F_3_NO_2_P: C, 14.73; H, 3.09; N, 8.59; found: C, 14.69; H, 2.89; N. 8.42; ^1^H NMR (300 MHz, D_2_O) δ_H_ 3.14 (d, ^2^*J*_HP_ = 11.4 Hz); ^31^P NMR (121 MHz) δ_P_ 12.2 (qt, ^2^*J*_PF_ = 96 Hz, ^2^*J*_PH_ = 11 Hz); ^19^F NMR (188 MHz) δ_F_ −76.1 (d, ^2^*J*_FP_ = 96 Hz); ^13^C NMR (125 MHz) δ_C_ 34.8 (d, ^1^*J*_CP_ = 105.6 Hz, *C*H_2_), 122,1 (qd, ^1^*J*_CF_ = 316.0 Hz, ^1^*J*_CP_ = 179.9 Hz, *C*F_3_).

### Hydrophosphinylation of azomethines

**The general procedure for the addition of acid 1 and ester 5 to substrates with the C=N double bond (III).** An equimolar mixture of an imine and **1** or **5** in DME (10 mL for 5 mmol) was stirred at room temperature under ^31^P NMR control until the ^31^P signals of starting P–H compounds disappeared. Sometimes an appropriate adduct precipitated and this was filtered off. The reaction mixture or the filtrate was then evaporated to dryness and the residue was worked up as described below for the individual substances.

Ethyl [(benzylamino)(phenyl)methyl](difluoromethyl)phosphinate (**18b**, Table 2, entry 2). Following the general procedure (III) a crude solid, obtained from **5** (0.49 g, 3.4 mmol) and **16b** (0.68g, 3.4 mmol) was extracted with boiling hexane (3 × 30 mL), this extract was evaporated to the dryness to afford **18b** as a yellowish solid (0.67 g, 58%); mp 85–93 °C, as a mixture of two diastereoisomers in an approximately 1:3.5 ratio due to ^1^H NMR (300 MHz, CDCl_3_) δ_H_ 0.97 (t, ^3^*J*_HH_ = 7.6 Hz, 0.7H, C*H*_3, _*minor isomer*), 1.25 (t, ^3^*J*_HH_ = 7.6 Hz, 2.3H, C*H*_3, _*major isomer*), 2.17 (br s, 1H, N*H*), 3.46 (d, *J*_AB_ = 12.6 Hz, 0.22H, C*H*_2_Ph, *minor isomer*), 3.51 (d, *J*_AB_ = 12.6 Hz, 0.77H, C*H*_2_Ph, *major isomer*), 3.78 (d, *J*_AB_ = 12.6 Hz, 1H, C*H*_2_Ph), 3.9 (dm, ^2^*J*_HP_ = 16.9 Hz, 0.22H, PC*H*, *minor isomer*), 4.08 (d, ^2^*J*_HP_ = 17.1 Hz, 0.8H, PC*H, major isomer*), 4.1–4.25 (m, 2H, OC*H*_2_), 5.88 (td, ^2^*J*_HF_ = 49.2 Hz, ^2^*J*_HP_ = 27.8 Hz, 0.8H, C*H*F_2_, *major isomer*), 6.25 (td, ^2^*J*_HF_ = 49.3 Hz, ^2^*J*_HP_ 27.6 Hz, 0.2H, C*H*F_2_, *minor isomer*), 7.15–7.40 (m, 10H, *H*_arom_); ^31^P NMR (81 MHz) δ_P_ 30.8 (m); ^19^F NMR (188 MHz) δ_F_ −132 to −140.5 (complex multiplet). To the viscous residue after extraction of **18b** water (20 mL) was added, resulting solution was decolorized with activated charcoal, filtrated and allowed to stand at 5 °C until crystallization completed, producing [(benzylamino)(phenyl)methyl](difluoromethyl)phosphinic acid (**19b**) as a white solid (0.32 g, 30%); mp 247 °C; Anal. calcd for C_15_H_16_F_2_NO_2_P: C, 57.88; H, 5.18; N, 4.50; found: C, 57.91; H, 5.04; N, 4.48; ^1^H NMR (300 MHz, DMSO-*d*_6_) δ_H_ 3.95 (d, *J*_AB_ = 12.9 Hz, 1H, C*H*_2_Ph), 4.04 (d, ^2^*J*_HP_ = 10.2 Hz, 1H, PC*H*), 4.10 (d, *J*_AB_ = 12.9 Hz, 1H, C*H*_2_Ph), 5.63 (td, ^2^*J*_HF_ = 49.2 Hz, ^2^*J*_HP_ = 21.9 Hz, 1H, C*H*F_2_), 7.30–7.42 (m, 10H, *H*_arom_); ^31^P NMR (81 MHz) δ_P_ 13.4 (tm, ^2^*J*_PF_ 68 Hz). An additional quantity of **19b** was obtained by hydrolysis of **18b** (0.67 g, 2 mmol) with 1N HCl (15 mL) at room temperature until the starting ester has dissolved. The resulting solution was evaporated to dryness at reduced pressure and the residue was recrystallized from water to give **19b** (0.6 g, 97%). The overall yield of **19b** is 0.92 g (87%).

See Supporting Information for details of the syntheses, characteristics and NMR spectra of all new compounds.

## Supporting Information

Experimental procedures and full characterization data for all new compounds including elemental analysis and ^1^H, ^31^P, ^19^F and ^13^C NMR are provided in the Supporting Information.

File 1 Experimental procedures, elemental analysis and NMR data.

File 2 NMR spectra of the most typical compounds.

File 3 NMR spectra of the most typical compounds (continuation).
