# Supplementary material for: A novel family of (1-aminoalkyl)(trifluoromethyl)- and -(difluoromethyl)phosphinic acids – analogues of α-amino acids
Source: Beilstein J Org Chem. 2014 Mar 26;10:722–31. doi: 10.3762/bjoc.10.66 (PMC3999843; doi:10.3762/bjoc.10.66)
Supplement: File 1 — Experimental procedures, elemental analysis and NMR data. [file Beilstein_J_Org_Chem-10-722-s001.pdf]

Supporting Information File 1
For

A novel family of (1-aminoalkyl)(trifluoromethyl)- and -(difluoromethyl)phosphinic acids – analogues of α-amino acids

Natalia V. Pavlenko[1], Tatiana I. Oos[1], Yurii L. Yagupolskii*[1], Igor I. Gerus[2], Uwe Doeller[3] and Lothar Willms[3]

Address: [1]Institute of Organic Chemistry National Academy of Sciences of Ukraine, Murmanskaya str. 5, 02660 Kiev-94, Ukraine, [2]Institute of Bioorganic Chemistry and Petrochemistry National Academy of Sciences of Ukraine, Murmanskaya str. 1, 02660 Kiev-94, Ukraine and [3]Bayer CropScience Aktiengesellschaft BCS AG-R-WC-WCC-C2 Weed Control Chemistry 2, Frankfurt, G836, 101, Germany

Email: Yurii L. Yagupolskii - Yagupolskii@ioch.kiev.ua

*Corresponding author

Experimental procedures, elemental analysis and NMR data.

[(Dibenzylamino)(phenyl)methyl](trifluoromethyl)phosphinic acid (**13b**). Following the general procedure (I) using benzaldehyde (2.12 g, 20 mmol) **13b** was obtained as a white solid (2.35 g, 28%); mp 280ºC; [Found: C, 62.88; H, 5.21; N. 3.50. $C_{22}H_{21}F_3NO_2P$ requires C, 63.00; H, 5.05; N, 3.34%]; NMR (DMSO-d_6) δ_H (300 MHz) 4.02 (2H, d, J_{AB} 12.5, CH_2Ph), 4.07 (1H, d, $^2J_{HP}$ 11.1, PC*H*), 4.14

(2H, d, J_{AB} 12.5, CH_2Ph), 7.37-7.39 (15 H, m, $H_{arom.}$); δ_P (121 MHz) 6.4 (qd, $^2J_{PF}$ 79, $^2J_{PH}$ 11); δ_F (188 MHz) -74.8 (d, $^2J_{FP}$ 79).

[1-(Dibenzylamino)ethyl](trifluoromethyl)phosphinic acid (**13c**). **13c** was synthesized according to the general procedure (I) and was characterized in a mixture with $Bn_2NH{\cdot}HCl$ (~1:7) by NMR spectroscopy. NMR (DMSO-d_6): δ_H (300 MHz) 1.44 (3H, dd, $^3J_{HP}$ 13.9, $^3J_{HH}$ 7.9, CH_3), 3.08 (1H, m, PC*H*), 4.28 (1H, d, J_{AB} 13.5, CH_2Ph), 4.47 (1H, d, J_{AB} 13.5, CH_2Ph), 7.38-7.63 (m, H_{arom}), 9.81 (br s, N*H*); δ_P (81 MHz) 5.9 (qm, $^2J_{PF}$ 80).

Ammonium [hydroxy(phenyl)methyl](trifluoromethyl)phosphinate (**15**). The filtrate after separating of **13b** was evaporated to the dryness. The addition to the residue of an excess of ethanolic ammonia solution gave the precipitate, which was filtered, crystallized and dried to give **15** as a white solid (3.1 g, 60%); mp 210ºC (dec.)(from EtOH); [Found: C, 37.55; H, 3.72; N, 5.42. $C_8H_{11}F_3NO_3P$ requires C, 37.66; H, 3.56; N, 5.49%]; NMR (D_2O): δ_H (300 MHz) 4.88 (1H, d, $^2J_{HP}$ 6.9, PC*H*), 7.22-7.31 (5H, m, H_{arom}); δ_P (121 MHz) 6.8 (qd, $^2J_{PF}$ 76, $^2J_{PH}$ 7); δ_F (188 MHz) -74.2 (d, $^2J_{FP}$ 76).

[Amino(phenyl)methyl](trifluoromethyl)phosphinic acid (**14b**). Following the general procedure (II) **14b** was obtained as a white powder (1.08 g, 90%); mp 263ºC (dec.); [Found: C, 39.96; H, 3.60; N. 5.69. $C_8H_9F_3NO_2P$ requires C, 40.18; H, 3.79; N, 5.86%]; NMR (D_2O): δ_H (300 MHz) 4.57 (1H, d, $^2J_{HP}$ 9.3, PC*H*), 7.2-7.25 (5H, m, H_{arom}); δ_P (121 MHz) 11.5 (qd, $^2J_{PF}$ 82, $^2J_{PH}$ 9); δ_F (188 MHz) -75.5 (d, $^2J_{FP}$ 82); δ_C (125 MHz) 51.9 (d, $^1J_{CP}$ 102.5, P*C*H), 122,5 (qd, $^1J_{CF}$ 316.8, $^1J_{CP}$ 176.2, *C*F_3), 128.4, 128.5, 129.4, 129.7, 130.0, 130.1.

[(Benzylamino)methyl](trifluoromethyl)phosphinic acid (**17a**) (Table 1, entry 1). Following the general procedure (III) a crude solid, obtained from **1** (1.28 g, 9.6 mmol) and **16a** (1.14 g, 3.2 mmol) was purified by recrystallization to give **17a** as a white solid (2.0 g, 83%); mp 282°C (from water); [Found: C, 42.93; H, 4.51; N. 5.66. $C_9H_{11}F_3NO_2P$ requires C, 42.70; H, 4.38; N, 5.53%]; NMR (DMSO-d_6): δ_H (300 MHz) 2.85 (2H, d, $^2J_{HP}$ 9.6, PCH_2), 4.16 (2H, s, CH_2Ph), 7.42 (3H, m,

H_{arom}), 7.50 (2H, m, H_{arom}), 9.07 (1H, br s, NH); δ_P (81 MHz) 4.6 (qt, $^2J_{PF}$ 85, $^2J_{PH}$ 10); δ_F (188 MHz) -74.2 (d, $^2J_{FP}$ 85).

[(Benzylamino)(phenyl)methyl](trifluoromethyl)phosphinic acid (**17b**) (Table 1, entry 2). Following the general procedure (III) a crude solid, obtained from **1** (0.13 g, 1.0 mmol) and **16b** (0.20 g, 1.0 mmol) was purified by recrystallization to give **17b** as a white solid (0.29 g, 87%); mp 293°C (from EtOH / water); [Found: C, 54.80; H, 4.62; N. 4.17. $C_{15}H_{15}F_3NO_2P$ requires C, 54.72; H, 4.59; N, 4.25%]; NMR (DMSO-d_6): δH (300 MHz) 3.95 (1H, d, J_{AB} 12.3, CH_2Ph), 4.05 (1H, d, $^2J_{HP}$ 10.0, PCH), 4.12 (1H, d, J_{AB} 12.3, CH_2Ph), 7.33-7.42 (10H, m, H_{arom}); δ_P (121 MHz) 7.9 (qd, $^2J_{PF}$ 79, $^2J_{PH}$ 10); δ_F (188 MHz) -74.0 (d, $^2J_{FP}$ 79).

[1-(Benzylamino)ethyl](trifluoromethyl)phosphinic acid (**17c**) (Table 1, entry 3). Following the general procedure (III) a crude solid, obtained from **1** (0.5 g, 3.7 mmol) and the freshly prepared **16c** (0.99 g, 7.4 mmol) was dissolved in acetone. Upon standing at 5 °C overnight **17c** crystallized, the crystals were filtered, dissolved in water and passed down an ion-exchange column to give after the evaporation of water **17c** as a white solid (0.58 g, 59%); mp 212ºC; [Found: C, 45.09; H, 5.01; N, 5.08. $C_{10}H_{13}F_3NO_2P$ requires C, 44.95; H, 4.90; N, 5.24%]; NMR (DMSO-d_6): δ_H (300 MHz) 1.33 (3H, dd, $^3J_{HP}$ 13.5, $^3J_{HH}$ 7.5, CH_3), 2.86-2.94 (1H, m, PCH), 4.21 (1H, d, J_{AB} 13.3, CH_2Ph), 4.29 (1H, d, J_{AB} 13.3, CH_2Ph), 7.38-7.46 (3H, m, H_{arom}), 7.50-7.54 (2H, m, H_{arom}), 8.88 (1H, br s, NH); δ_P (81 MHz) 4.6 (qm, $^2J_{PF}$ 79). δ_F (188 MHz) -72.8 (d, $^2J_{FP}$ 79).

[1-(Benzylamino)-2-methylpropyl](trifluoromethyl)phosphinic acid (**17d**) (Table 1, entry 4). Following the general procedure (III) a crude solid, obtained from **1** (0.20 g,1.5 mmol) and **16d** (0.24 g, 1.5 mmol) was triturated with acetone to afford **17d** of a sufficient purity as a white solid (0.40 g, 92%), mp 207ºC; [Found: C, 48.77; H, 5.79; N, 4.78. $C_{12}H_{17}F_3NO_2P$ requires C, 48.82; H, 5.80; N, 4.74%]; NMR (DMSO-d_6): δ_H (300 MHz) 1.03 (3H, d, $^3J_{HH}$ 6.6, CH_3), 1.09 (3H, d, $^3J_{HH}$ 6.6, CH_3), 2.24 (1H, m, CHMe$_2$), 2.81 (1H, m, PCH), 3.25 (1H, br s, NH), 4.24 (1H, d, J_{AB} 13.5, CH_2Ph), 4.37 (1H, d, J_{AB} 13.5, CH_2Ph), 7.34-7.46 (3H, m,

H_{arom}), 7.51-7.55 (2H, m, H_{arom}), 8.64 (1H, br s, OH); δ_P (81 MHz) 6.2 (qm, $^2J_{PF}$ 79); δ_F (188 MHz) -74.2 (d, $^2J_{FP}$ 79).

Pyrrolidin-2-yl-(trifluoromethyl)phosphinic acid (**14e**) (Table 1, entry 5). Following the general procedure (III) a crude product, obtained from **1** (1.0 g, 7.5 mmol) and **16e** (0.52 g, 2.5 mmol) was dissolved in ethanolic ammonia solution until pH 8–9 and this solution was evaporated to the dryness. The residue was triturated with acetone affording a solid, which was dissolved in water and passed down an ion-exchange column to give after the evaporation of water **14e** as a white solid (1.0 g, 66%); mp 293ºC; [Found: C, 29.40; H, 4.52; N, 6.89. $C_5H_9F_3NO_2P$ requires C, 29.52; H, 4.47; N, 6.89%]; NMR (D_2O): δ_H (500 MHz) 1.9-2.3 (4H, m, CH_2-CH_2), 3.34 (2H, m, CH_2-N), 3.74 (1H, m, CH-P); δ_P (81 MHz) 12.1 (qm, $^2J_{PF}$ 94); δ_F (188 MHz) -76.4 (d, $^2J_{FP}$ 94); δ_C (125 MHz) 23.8 (d, $^2J_{CP}$ 8, C-3), 24.9 (C-4), 47.8 (d, $^3J_{CP}$ 5, C-5), 53.4 (d, $^1J_{CP}$ 107.2, C-2), 122.2 (qd, $^1J_{CF}$ 316.2, $^1J_{CP}$ 177.3, CF$_3$)

[(Benzylamino)methyl](difluoromethyl)phosphinic acid (**19a**) (Table 2, entry 1). Following the general procedure (III) a mixture of **5** (0.56 g, 3.9 mmol) and **16a** (0.46 g, 1.3 mmol) in DME (10 mL) was heated at 50°C for 24 h to give a complex mixture with a major product ethyl [(benzylamino)-methyl](difluoromethyl)phosphinate (**18a**) according to ^{31}P NMR: δP (121 MHz, DME) 33.7 (tm, $^2J_{PF}$ 73). This mixture was chromatographed on SiO_2 with eluent ethylacetate-methanol (1:3) to afford **19a** as a white solid (0.62 g, 68%); mp 226°C; [Found: C, 46.13; H, 5.33; N. 5.79. $C_9H_{12}F_2NO_2P$ requires C, 45.94; H, 5.14; N, 5.96%]; NMR (DMSO-d_6): δ_H (300 MHz) 2.75 (2H, d, $^2J_{HP}$ 7.2, PCH_2), 4.10 (2H, s, CH_2Ph), 5.88 (1H, td, $^2J_{HF}$ 48.7, $^2J_{HP}$ 21.5, CHF$_2$), 7.37-7.41 (3H, m, H_{arom}), 7.51-7.55 (2H, m, H_{arom}), 9.21 (1H, br s, NH); δ_P (81 MHz) 11.8 (tm, $^2J_{PF}$ 70); δ_F (188 MHz) -135.8 (dd, $^2J_{FP}$ 70, $^2J_{FH}$ 49).

Ethyl [1-(benzylamino)ethyl](difluoromethyl)phosphinate (**18c**) (Table 2, entry 3). Following the general procedure (III) a crude product from **5** (0.30 g, 2.1 mmol) and **16c** (0.28 g, 2.1 mmol) was worked up as described above for **18b** to afford **18c** as a yellowish semisolid (0.27 g, 46%), as a mixture of two

diastereoisomers in an approximately 3:2 ratio due to NMR ($CDCl_3$): δ_H (300 MHz) 1.24-1.38 (6H, m, OCH_2CH_3 and $PCHCH_3$), 2.95-3.15 (1H, m, PC*H*), 3.64-4.07 (2H, m, CH_2Ph), 4.15-4.30 (2H, m, OCH_2), 6.14 (0.6H, td, $^2J_{HF}$ 49.5, $^2J_{HP}$ 25.4, CHF_2 , *major isomer*), 6.22 (0.4 H, td, $^2J_{HF}$ 49.3, $^2J_{HP}$ 26.0, CHF_2, *minor isomer*), 7.18-7.32 (5H, m, H_{arom}); δ_P (81 MHz) 35.7 (m); δ_F (188 MHz) -130 ÷ -142 (complex multiplet). From the residue after extraction of **18c** [1-(benzylamino)ethyl](difluoro-methyl)phosphinic acid (**19c**) was obtained by the analogy with **19b** as a white solid (0.22 g, 42%); mp 216ºC; [Found: C, 47.96; H, 5.72; N, 5.68. $C_{10}H_{14}F_2NO_2P$ requires C, 48.12; H, 5.66; N, 5.62%]; NMR (DMSO-d_6): δ_H (300 MHz) 1.35 (3H, dd, $^3J_{HP}$ 12.9, $^3J_{HH}$ 7.4, CH_3), 2.98 (1H, dq, $^2J_{HP}$ 15.0, $^3J_{HH}$ 7.4, PC*H*), 4.23 (1H, d, J_{AB} 9.1, CH_2Ph), 4.32 (1H, d, J_{AB} 9.1, CH_2Ph), 5.91 (1H, td, $^2J_{HF}$ 49.2, $^2J_{HP}$ 20.4, CHF_2), 7.39-7.45 (3H, m, H_{arom}), 7.52-7.59 (2H, m, H_{arom}); δ_P (81 MHz) 16.6 (tm, $^2J_{PF}$ 75); δ_F (188 MHz) -134.5 (1F, ddd, J_{AB} 349, $^2J_{FP}$ 75, $^2J_{HF}$ 49), -136.5 (1F, ddd, J_{AB} 349, $^2J_{FP}$ 75, $^2J_{FH}$ 49). By hydrolysis of **18c** (0.27 g, 0.98 mmol) the additional quantity of **19c** (0.20 g, 83%) was obtained. The overall yield of **19c** is 0.42 g (80%).

Ethyl [1-(benzylamino)-2-methylpropyl)(difluoromethyl)phosphinate (**18d**) (Table 2, entry 4). Following the general procedure (III) a crude solid, obtained from **5** (0.29 g, 2.0 mmol) and **16d** (0.32 g, 2.0 mmol) was worked up as described above for **18b** to afford **18d** as a yellowish semisolid (0.22 g, 36%) as a mixture of two diastereoisomers in an approximately 3:2 ratio due to NMR ($CDCl_3$): δ_H (300 MHz) 1.05 (6H, m, $PCHCH_3$), 1.29 (3H, m, OCH_2CH_3), 2.19 (1H, m, $CHMe_2$), 2.86 (0.6H, ddm, $^2J_{HP}$ 12.9, $^3J_{HH}$ 5.2, PC*H*, *major isomer*), 2.96 (0.4H, ddm, $^2J_{HP}$ 11.6, $^3J_{HH}$ 4.8, PC*H*, *minor isomer*), 3.72-3.95 (2H, m, CH_2Ph), 4.02-4.30 (2H, m, OCH_2), 6.08 (0.4H, td, $^2J_{HF}$ 49.6, $^2J_{HP}$ 24.8, CHF_2, *minor isomer*), 6.18 (0.6H, td, $^2J_{HF}$ 49.6, $^2J_{HP}$ 25.2, CHF_2, *major isomer*), 7.18-7.32 (5H, m, H_{arom}); δ_P (81 MHz) 35.4 (0.4P, m, *minor isomer*), 36.8 (0.6P, m, *major isomer*); δ_F (188 MHz) -132 ÷ -140.5 (complex multiplet). From the residue after extraction of **18d** [1-(benzylamino)-2-methyl-propyl]-(difluoromethyl)phosphinic acid (**19d**) was obtained by analogy with **19b** as a white solid (0.27 g, 48%); mp 242ºC; [Found: C, 52.16; H, 6.69; N, 4.89.

$C_{12}H_{18}F_2NO_2P$ requires C, 51.99; H, 6.54; N, 5.05%]; NMR (DMSO-d_6): δ_H (300 MHz) 0.98 (3H, d, $^3J_{HH}$ 6.9, C*H*$_3$), 1.06 (3H, d, $^3J_{HH}$ 6.9, C*H*$_3$), 2.10-2.30 (1H, m, C*H*Me$_2$), 2.68-2.78 (1H, m, PC*H*), 4.20 (1H, d, J_{AB} 13.1, C*H*$_2$Ph), 4.30 (1H, d, J_{AB} 13.1, C*H*$_2$Ph), 5.72 (1H, td, $^2J_{HF}$ 49.5, $^2J_{HP}$ 19.4, C*H*F$_2$), 7.36-7.44 (3H, m, H_{arom}), 7.48-7.56 (2H, m, H_{arom}); δ_P (81 MHz) 12.3 (tm, $^2J_{PF}$ 81); δ_F (188 MHz) -132.8 (1F, ddd, J_{AB} 348, $^2J_{FP}$ 81, $^2J_{HF}$ 49), -134.1 (1F, ddd, J_{AB} 348, $^2J_{FP}$ 81, $^2J_{HF}$ 49). By hydrolysis of **18d** (0.22 g, 0.72 mmol) the additional quantity of **19d** (0.19 g, 95%) was obtained. The overall yield of **19d** is 0.46 g (82%).

(Difluoromethyl)pyrrolidin-2-ylphosphinic acid (**20e**) (Table 2, entry 5). Following the general procedure (III) a crude product, obtained from **5** (0.54 g, 3.8 mmol) and **16e** (0.26 g, 1.25 mmol) was worked up as described for **14e** to afford **20e** as a white solid (0.54 g, 79%); mp 246°C; [Found: C, 32.30; H, 5.52; N, 7.68. $C_5H_{10}F_2NO_2P$ requires C, 32.44; H, 5.45; N, 7.57%]; NMR (D_2O): δ_H (500 MHz) 1.90-2.02 (1H, m, C*H*$_2$), 2.04-2.12 (2H, m, C*H*$_2$), 2.20-2.30 (1H, m, C*H*$_2$), 3.33 (2H, t, $^3J_{HH}$ 6.5, C*H*$_2$N), 3.71 (1H, m, C*H*P), 5.95 (1H, td, $^2J_{HF}$ 48.5, $^2J_{HP}$ 21.0, C*H*F$_2$); δ_P (81 MHz) 17.6 (tm, $^2J_{PF}$ 78); δ_F (188 MHz) -139.2 (1F, ddd, J_{AB} 351, $^2J_{FP}$ 78, $^2J_{FH}$ 49), -137.3 (1F, ddd, J_{AB} 351, $^2J_{FP}$ 78, $^2J_{FH}$ 49); δ_C (125 MHz) 24.3 (d, $^2J_{CP}$ 8, *C*-3), 24.9 (*C*-4), 47.8 (d, $^3J_{CP}$ 5, *C*-5), 53.4 (d, $^1J_{CP}$ 107.2, *C*-2), 122,2 (qd, $^1J_{CF}$ 316.2, $^1J_{CP}$ 177.3, *C*F$_3$)

(1-Aminoethyl)(trifluoromethyl)phosphinic acid (**14c**) (Table 1, entry 3). Following the general procedure (II) **14c** was obtained as a white powder, mp 255°C (dec.); [Found: C, 20.28; H, 3.89; N. 7.79. $C_3H_7F_3NO_2P$ requires C, 20.35; H, 3.98; N, 7.91%]; NMR (D_2O): δ_H (300 MHz) 1.30 (3H, dd, $^3J_{HP}$ 14.7, $^3J_{HH}$ 7.5, C*H*$_3$), 3.46 (1H, m, PC*H*); δ_P (121 MHz) 15.2 (qm, $^2J_{PF}$ 91); δ_F (188 MHz) -73.8 (d, $^2J_{FP}$ 91); δ_C (125 MHz) 31.8 (*C*H$_3$); 44.1 (d, $^1J_{CP}$ 105.5, P*C*H), 122,1 (qd, $^1J_{CF}$ 316.9, $^1J_{CP}$ 179.8, *C*F$_3$).

(1-Amino-2-methylpropyl)(trifluoromethyl)phosphinic acid (**14d**) (Table 1, entry 4). Following the general procedure (II) **14d** was obtained as a white powder, mp 225°C (dec.); [Found: C, 29.22; H, 5.31; N. 6.78. $C_5H_{11}F_3NO_2P$ requires C, 29.28; H, 5.41; N, 6.83%]; NMR (D_2O): δ_H (500 MHz) 1.06 (3H, d,

$^3J_{HH}$ 7, CH_3), 1.09 (3H, d, $^3J_{HH}$ 7, CH_3), 2.37 (1H, m, CHMe$_2$), 3.43 (1H, dd, $^2J_{HP}$ 8.1, $^3J_{HH}$ 4.5, PCH); δ_P (121 MHz) 14.8 (qt, $^2J_{PF}$ 93, $^2J_{PH}$ = $^3J_{PH}$ = 8); δ_F (188 MHz) -75.3 (d, $^2J_{FP}$ 93); δ_C (125 MHz) 17.6 (d, $^3J_{CP}$ 4, CH$_3$), 19.4 (d, $^3J_{CP}$ 8.5, CH$_3$), 27.1 (CHMe$_2$), 52.8 (d, $^1J_{CP}$ 105.0, PCH), 122,2 (qd, $^1J_{CF}$ 317.5, $^1J_{CP}$ 173.5, CF$_3$).

(Aminomethyl)(difluoromethyl)phosphinic acid (**20a**) (Table 2, entry 1). Following the general procedure (II) **20a** was obtained as a white powder, mp 242°C; [Found: C, 16.62; H, 4.29; N 9.59. $C_2H_6F_2NO_2P$ requires C, 16.56; H, 4.17; N 9.66%]; NMR (D_2O): δ_H (300 MHz) 3.03 (2H, d, $^2J_{HP}$ 10.4, CH_2), 5.77 (1H, td, $^2J_{HF}$ 49.2, $^2J_{HP}$ 22.2, CHF$_2$); δ_P (121 MHz) 16.1 (tdt, $^2J_{PF}$ 78, $^2J_{PH}$ 22 and 10); δ_F (188 MHz) -136.7 (dd, $^2J_{FP}$ 78, $^2J_{FH}$ 49); δ_C (125 MHz) 35.3 (d, $^1J_{CP}$ 103.2, CH$_2$), 114.5 (td, $^1J_{CF}$ 246.8, $^1J_{CP}$ 129.3, CHF$_2$).

[Amino(phenyl)methyl](difluoromethyl)phosphinic acid (**20b**) (Table 2, entry 2). Following the general procedure (II) **20b** was obtained as a white powder, mp 256°C; [Found: C, 43.59; H, 4.68; N. 6.29. $C_8H_{10}F_2NO_2P$ requires C, 43.45; H, 4.56; N, 6.33%]; NMR (D_2O): δ_H (300 MHz) 4.48 (1H, d, $^2J_{HP}$ 9.6, PCH), 5.78 (1H, td, $^2J_{HF}$ 48.9, $^2J_{HP}$ 22.5, CHF$_2$), 7.27-7.30 (5H, m, H_{arom}); δ_P (121 MHz) 15.9 (tdd, $^2J_{PF}$ 88, $^2J_{PH}$ 22 and 10); δ_F (376 MHz) -135.8 (symm. complex multiplet); δ_C (125 MHz) 51.5 (d, $^1J_{CP}$ 94.2, PCH), 114.8 (td, $^1J_{CF}$ 257.8, $^1J_{CP}$ 138.3, CF$_3$), 128.1, 129.3, 129.4, 130.5.

(1-Aminoethyl)(difluoromethyl)phosphinic acid (**20c**) (Table 2, entry 3). Following the general procedure (II) **20c** was obtained as a white powder, mp 255°C; [Found: C, 22,48; H, 4.96; N. 8.92. $C_3H_8F_2NO_2P$ requires C, 22.65; H, 5.07; N, 8.81%]; NMR (D_2O): δ_H (500 MHz) 1.41 (3H, dd, $^3J_{HP}$ 13.5, $^3J_{HH}$ 7.5, CH_3), 3.52 (1H, m, PCH), 5.97 (1H, td, $^2J_{HF}$ 48.5, $^2J_{HP}$ 22.5, CHF$_2$); δ_P (121 MHz) 18.6 (tm, $^2J_{PF}$ 75); δ_F (376 MHz) -136.6 (1F, ddd, J_{AB} 348.8, $^2J_{FP}$ 74.9, $^2J_{FH}$ 48.5), -134.9 (1F, ddd, J_{AB} 348.8, $^2J_{FP}$ 74.9, $^2J_{FH}$ 48.5); δ_C (125 MHz) 12.1 (CH$_3$); 43.3 (d, $^1J_{CP}$ 100, PCH), 114.5 (td, $^1J_{CF}$ 248.2, $^1J_{CP}$ 136.3, CHF$_2$).

(1-Amino-2-methylpropyl)(difluoromethyl)phosphinic acid (**20d**) (Table 2, entry 4). Following the general procedure (II) **20d** was obtained as a white powder, mp 232°C; [Found: C, 32.12; H, 6.37; N. 7.61. $C_5H_{12}F_2NO_2P$ requires C,

32.09; H, 6.46; N, 7.49%]; NMR (D_2O): δ_H (300 MHz) 0.89 (3H, d, $^3J_{HH}$ 7.9, CH_3), 0.94 (3H, d, $^3J_{HH}$ 7.9, CH_3), 2.18 (1H, m, CHMe$_2$), 3.18 (1H, m, PCH), 5.75 (1H, td, $^2J_{HF}$ 48.9, $^2J_{HP}$ 22.5, CHF$_2$); δ_P (81 MHz) 19.2 (tm, $^2J_{PF}$ 77); δ_F (377 MHz) -135.3 (symm. complex multiplet, J_{AB} 346); δ_C (125 MHz) 23.8 (d, $^3J_{CP}$ 6.9, CH$_3$), 24.8 (CH$_3$), 47.7 (CHMe$_2$), 53.2 (d, $^1J_{CP}$ 99.3, PCH), 114.9 (td, $^1J_{CF}$ 257.8, $^1J_{CP}$ 139.6, CHF$_2$).

The reaction of the acid **1** with the imine **21**. A mixture of **1** (0.10 g, 0.75 mmol) and **21** [1] (0.15 g, 0.75 mmol) in DME (5 mL) was heated at 50 °C under stirring for 48 h. After cooling to room temperature the formed precipitate was filtered, washed with acetone and dried to give **14b (**0.06 g, 32%).

The reactions of the acids **1** and **6** with the imines **22** and **27** (general procedure IV). An equimolar mixture of **1** or **6** and **22** or **27** in DME (5 mL per 1 mmol) was stirred at 50 °C under monitoring with ^{31}P NMR until the signals of **1** or **6** disappeared. After cooling to room temperature the formed precipitate was filtered, thoroughly washed with acetone and dried in vacuo.

(1-Amino-2-ethoxy-2-oxoethyl)(trifluoromethyl)phosphinic acid (**23**). Following the general procedure (IV) using **1** (0.95 g, 7.1 mmol) and **22** [2] (11.42 g. 7.1 mmol) **23** was obtained as a white solid (1.13 g, 68%), mp 241°C (dec.). [Found: C, 25.48; H, 3.83; N. 5.96. $C_5H_9F_3NO_4P$ requires C, 25.54; H, 3.86; N, 5.96%]; NMR (D_2O): δ_H (300 MHz) 1.10 (3H, t, $^3J_{HH}$ 6.8, CH_3), 3.92 (1H, d, $^2J_{HP}$ 11.7, PCH), 4.11 (2H, q, $^3J_{HH}$ 6.8, CH_2); δ_P (121 MHz) 6.2 (dq, $^2J_{PF}$ 86, $^2J_{PH}$ 12); δ_F (188 MHz) -72.2 (d, $^2J_{FP}$ 86).

Sodium salt of **23**. NMR (D_2O): δ_H (500 MHz) 1.22 (3H, t, $^3J_{HH}$ 7.0, CH_3), 4.20 (2H, q, $^3J_{HH}$ 7.0, CH_2); δ_C (125 MHz) 13.3 (CH3), 53.9 (m, CH), 63.0 (CH$_2$), 122,4 (qd, $^1J_{CF}$ 318, $^1J_{CP}$ 171, CF$_3$), 171.3 (O=C).

(1-Amino-2-ethoxy-2-oxoethyl)(difluoromethyl)phosphinic acid (**24**). Following the general procedure (IV) using **6** (0.83 g, 7.2 mmol) and **22** [2] (1.44 g, 7.2 mmol) **24** was obtained as a white solid (0.95 g, 61%), mp 223°C (dec.). [Found: C, 27.77; H, 4.74; N. 6.40. $C_5H_{10}F_2NO_4P$ requires C, 27.66; H, 4.64; N, 6.45%]; NMR (D_2O): δ_H (300 MHz) 1.11 (3H, t, $^3J_{HH}$ 6.9, CH_3), 4.08 (2H, q, $^3J_{HH}$

6.9, CH_2), 4.31 (1H, d, $^2J_{HP}$ 14.1, PCH), 5.9 (1H, td, $^2J_{HF}$ 48.5, $^2J_{HP}$ 25.8, CHF$_2$); δ_P (121 MHz) 12.1 (tdd, $^2J_{PF}$ 80, $^2J_{PH}$ 26 and 14); δ_F (282 MHz) -135.3 (1F, ddd, J_{AB} 347, $^2J_{FP}$ 80, $^2J_{FH}$ 49), -137.1 (1F, ddd, J_{AB} 347, $^2J_{FP}$ 80, $^2J_{FH}$ 49); δ_C (125 MHz) 13.2 (CH$_3$), 51.4 (d, $^1J_{CP}$ 78, CH), 64.1 (CH$_2$), 114.0 (td, $^1J_{CF}$ 259, $^1J_{CP}$ 148, CHF$_2$), 166.4 (O=C).

Amino[(difluoromethyl)(hydroxy)phosphoryl]acetic acid (**25**). A mixture of **24** (0.52 g, 2.4 mmol) and Me_3SiONa (0.54 g, 4.8 mmol) in DME (10 mL) was stirred at 50 °C for 24 h. After cooling to room temperature the formed precipitate was filtered, washed with acetone and dissolved in water. The resulting solution was discolored with charcoal and then passed down an ion-exchange column. Water was evaporated under reduced pressure, the residue was thoroughly dried in vacuo to afford **25** as a white powder (0.16 g, 35 %), mp 265°C (dec.). [Found: C, 18.98; H, 3.25; N. 7.38. $C_3H_6F_2NO_4P$ requires C, 19.06; H, 3.20; N, 7.41%]; NMR (D_2O): δ_H (300 MHz) 3.48 (1H, d, $^2J_{HP}$ 12.5, PCH), 5.9 (1H, td, $^2J_{HF}$ 50.7, $^2J_{HP}$ 25.2, CHF$_2$); δ_P (121 MHz) 18.2 (tdd, $^2J_{PF}$ 78, $^2J_{PH}$ 25, $^2J_{PH}$ 12); δ_F (282 MHz) -133.1 (1F, ddd, J_{AB} 338, $^2J_{FP}$ 78, $^2J_{FH}$ 51), -135.0 (1F, ddd, J_{AB} 338, $^2J_{FP}$ 78, $^2J_{FH}$ 51).

Methyl 2-[hydroxy(trifluoromethyl)phosphoryl]valinate (**27**). Following the general procedure (IV) using **1** (1.0 g, 7.4 mmol) and **26** [3] (0.96 g, 7.4 mmol) **27** was obtained as a white solid (1.24 g, 63 %), mp 218°C (dec.). [Found: C, 31.99; H, 5.10; N. 5.29. $C_7H_{13}F_3NO_4P$ requires C, 31.95; H, 4.98; N, 5.32%]; NMR (D_2O): δ_H (300 MHz) 0.76 (3H, d, $^3J_{HH}$ 7.0, CCH_3), 0.99 (3H, d, $^3J_{HH}$ 7.0, CCH_3), 2.69 (1H, septet of doublets, $^3J_{HH}$ 7.0, $^3J_{HP}$ 5.9, CHMe$_2$), 3.70 (3H, s, OCH_3); δ_P (121 MHz) 10.4 (dq, $^2J_{PF}$ 95, $^3J_{PH}$ 6); δ_F (282 MHz) -72.1 (d, $^2J_{FP}$ 95).

Methyl 2-[(difluoromethyl)(hydroxy)phosphoryl]valinate (**28**). Following the general procedure (IV) using **6** (0.45 g, 3.9 mmol) and **26** [3] (0.50 g, 3.9 mmol) **28** was obtained as a white solid (0.38 g, 40 %), mp 209°C (dec.). [Found: C, 34.30; H, 5.80; N. 5.80. $C_7H_{14}F_2NO_4P$ requires C, 34.29; H, 5.76; N, 5.71%]; NMR (D_2O): δ_H (300 MHz) 0.78 (3H, d, $^3J_{HH}$ 6.8, CCH_3), 1.00 (3H, d, $^3J_{HH}$ 6.8, CCH_3), 2.70 (1H, septet of doublets, $^3J_{HH}$ 6.8, $^3J_{HP}$ 6.0, CHMe$_2$), 3.72 (3H, s, OCH_3), 5.82 (1H, td, $^2J_{HF}$ 48.6, $^2J_{HP}$ 25.2, CHF$_2$); δ_P (121 MHz) 14.7 (tdd, $^2J_{PF}$ 76,

$^{2}J_{PH}$ 25, $^{3}J_{PH}$ 6); δ_F (282 MHz): -133.2 (1F, ddd, J_{AB} 349, $^{2}J_{FP}$ 76, $^{2}J_{FH}$ 49), -135.3 (1F, ddd, J_{AB} 349, $^{2}J_{FP}$ 76, $^{2}J_{FH}$ 49).

The reaction of the acid **1** with aminoacrylate **29**. Following the general procedure (IV) using **1** (0.51 g, 3.8 mmol) and **29** [4] (0.81 g, 3.8 mmol) ethyl 2-[hydroxy(trifluoromethyl)phosphoryl]alaninate (**31**) was obtained as a white solid (0.56 g, 59%), mp 242ºC (dec.). [Found: C, 28.79; H, 4.38; N. 5.66. $C_6H_{11}F_3NO_4P$ requires C, 28.93; H, 4.45; N, 5.62 %]; NMR (DMSO-d$_6$): δ_H (300 MHz) 1.20 (3H, t, $^{3}J_{HH}$ 6.9, CH_2CH_3), 1.55 (3H, d, $^{3}J_{HP}$ 12.9, PCCH_3), 4.13 (2H, q, $^{3}J_{HH}$ 6.9, CH_2); δ_P (121 MHz) 7.2 (qq, $^{2}J_{PF}$ 84, $^{3}J_{PH}$ 13); δ_F (282 MHz) -69.3 (d, $^{2}J_{FP}$ 84).

2-[Hydroxy(trifluoromethyl)phosphoryl]alanine (**32**). Hydrolysis of **31** (1.2 g, 4.8 mmol) with 5 N HCl (10 mL) was performed at room temperature for 48 h under stirring. Resulting solution was evaporated to the dryness, the residue was dissolved in the ethanolic ammonia solution at 0 ºC until pH 7–8. Ethanol was evaporated to the dryness and the residue was triturated with acetone to afford yellowish solid. This solid was dissolved in water; the resulting solution was discolored with charcoal and then passed down an ion-exchange column. After the evaporation of water the residue was thoroughly dried in vacuo to afford **32** as a white powder (0.58 g, 54%), mp 266ºC (dec.). [Found: C, 21.56; H, 3.24; N. 6.32. $C_4H_7F_3NO_4P$ requires C, 21.73; H, 3.19; N, 6.34 %]; NMR (D_2O): δ_H (500 MHz) 1.58 (3H, d, $^{3}J_{HP}$ 14.5, CH_3); δ_P (121 MHz) 21.0 (qq, $^{2}J_{PF}$ 82, $^{3}J_{PH}$ 14); δ_F (282 MHz) -70.3 (d, $^{2}J_{FP}$ 82); δ_C (125 MHz) 20.8 (*C*H3), 75.3 (d, $^{1}J_{CP}$ 115, P*C*), 122.9 (qd, $^{1}J_{CF}$ 318, $^{1}J_{CP}$ 166, *C*F_3), 175.2 (O=*C*).

The reaction of a mixture of the esters **3** and **4** with aminoacrylic acid **33.** The freshly distilled mixture of **3** (3.69 g, 19.4 mmol) and **4** (2.10 g, 12.9 mmol) (bp 65–75 °C, 70 mm Hg) [5] and **33** (1.66 g, 12.9 mmol) was stirred at 20 °C for 72 h under the ^{31}P NMR control. The resulting viscous mixture was diluted with 25 ml ether and the formed precipitate was filtered, washed with acetone and dried in vacuo to afford [1-(acetylamino)ethyl](trifluoromethyl)phosphinic acid (**37**) as a white solid (0.75 g, 18%), mp 199ºC (dec.). [Found: C, 27.41; H, 4.14; N. 6.39.

$C_5H_9F_3NO_3P$ requires C, 27.62; H, 4.24; N, 6.16 %]; NMR (D_2O): δ_H (300 MHz) 1.07 (3H, dd, $^3J_{HP}$ 14.7, $^3J_{HH}$ 7.5, CHCH_3), 1.75 (3H, s, O=CCH_3), 4.07 (1H, dq, $^2J_{HP}$ 10.3, $^3J_{HH}$ 7.5, PC*H*); δ_P (121 MHz) 19.4 (qqd, $^2J_{PF}$ 85, $^3J_{PH}$ 15, $^2J_{PH}$ 10); δ_F (282 MHz) -73.3 (d, $^2J_{FP}$ 85). All volatiles from the filtrate after the isolation of **37** were removed by heating to 50 °C at a pressure of 0.1 mm Hg. The residue was fractionated by the silica gel column chromatography (hexane/EtOAc 10:1 to EtOAc/EtOH 1:1, gradient).

Ethyl *N*-acetyl-2-[hydroxy(trifluoromethyl)phosphoryl]alaninate (**35**) was isolated with the eluent EtOAc/EtOH 2:1 as a white solid (0.26 g, 7%), mp 203°C (dec.). [Found: C, 33.22; H, 4.68; N. 4.70. $C_8H_{13}F_3NO_5P$ requires C, 33.00; H, 4.50; N, 4.81 %]; NMR (DMSO-d_6): δ_H (300 MHz) 1.11 (3H, t, $^3J_{HH}$ 7.0, CH_2CH_3), 1.48 (3H, d, $^3J_{HP}$ 14.1, PCCH_3), 1.81 (3H, s, O=CCH_3), 4.18 (2H, q, $^3J_{HH}$ 7.0, CH_2); δ_P (121 MHz) 19.2 (qq, $^2J_{PF}$ 81, $^3J_{PH}$ 14); δ_F (282 MHz) -74.1 (d, $^2J_{FP}$ 81).

Ethyl [1-(acetylamino)ethyl](trifluoromethyl)phosphinate (**36**) was isolated with the eluent hexane/ethyl acetate 2:1 as a yellowish viscous liquid (1.28 g, 40%) as a mixture of two diastereoisomers in an approximately 7:8 ratio due to NMR ($CDCl_3$): δ_H (300 MHz) 1.25-1.45 (6H, m, CHCH_3 and CH_2CH_3), 1.96 (1.4H, s, O=CCH_3, *minor isomer*), 1.99 (1.6H, s, O=CCH_3, *major isomer*), 4.15-4.42 (2H, m, OCH_2), 4.68-4.84 (1H, m, PC*H*), 7.55 (0.45H, br s, N*H*, *minor isomer*), 7.76 (0.55 H, br s, N*H*, *major isomer*); δ_P (121 MHz) 27.6 (qm, *minor isomer*), 28.4 (qm, *major isomer*); δ_F (282 MHz) -72.1 (1.6 F, d, $^2J_{FP}$ 87, *major isomer*), -72.9 (1.4F, d, $^2J_{FP}$ 87, *minor isomer*). Hydrolysis of **36** (1.28 g, 5.2 mmol) with 5N HCl (15 mL) at room temperature for 24 h gave **37** (1.07 g, 95%). The overall yield of **37** from **33** was 1.82 g (65%). The *N*-deprotection of **37** (1.5 g, 6.9 mmol) was performed with 5 N HCl (20 mL) in an ampoule with Teflon stopcock at 130ºC for 8 h to give **14c** (0.41 g, 34%).

The reaction of the acid **1** with the malonate **38**. A mixture of **1** (1.05 g, 7.8 mmol) and **38** (1.79 g, 7.8 mmol) in CH_3CN (15 mL) was stirred at room temperature for a week under the ^{31}P NMR control and then cooled to –20 °C. The formed precipitate was filtered and recrystallized to give [1-(acetylamino)-3-

ethoxy-2-(ethoxycarbonyl)-3-oxopropyl](trifluoromethyl)phosphinic acid (**39**) as a white solid (1.26 g, 44%), mp 176°C (dec.) (from a wet dioxane). [Found: C, 36.24; H, 4.84; N. 3.90. $C_{11}H_{17}F_3NO_7P$ requires C, 36.37; H, 4.72; N, 3.86 %]; NMR (DMSO-d_6): δ_H (300 MHz) 1.16 (3H, t, $^3J_{HH}$ 6.9, CH_2CH_3), 1.18 (3H, t, $^3J_{HH}$ 6.9, CH_2CH_3), 1.81 (3H, s, O=CCH_3), 3.72 (1H, t, $^3J_{HH}$ = $^3J_{HP}$ = 8.0, PCHC*H*), 4.07 (4H, q, $^3J_{HH}$ 6.9, CH_2), 4.87 (1H, dd, $^2J_{HP}$ 17.1, $^3J_{HH}$ 8.0, PC*H*), 7.62 (1H, br s, N*H*); δ_P (121 MHz) 12.5 (qm, $^2J_{PF}$ 87); δ_F (282 MHz) -73.2 (d, $^2J_{FP}$ 87).

3-(Acetylamino)-3-[hydroxy(trifluoromethyl)phosphoryl]propanoic acid (**40**). A suspension of **39** (0.84 g, 2.3 mmol) in 5N HCl (10 mL) was heated to reflux for 12 h. The resulting solution was evaporated to the dryness, and the residue was triturated with acetone and dried in vacuo to afford **40** as a white solid (0.50 g, 82%), mp 178°C (dec.). [Found: C, 27.48; H, 3.57; N, 5.20. $C_6H_9F_3NO_5P$ requires C, 27.39; H, 3.45; N, 5.32 %]; NMR (DMSO-d_6): δ_H (300 MHz) 2.01 (3H, s, O=CCH_3), 2.78 (1H, ddd, J_{AB} 17.5, $^3J_{HP}$ 10.8, $^3J_{HH}$ 4.5, CH_2), 2.94 (1H, ddd, J_{AB} 17.5, $^3J_{HH}$ 4.5, $^3J_{HP}$ 2.7, CH_2), 4.42-4.50 (1H, m, PC*H*); δ_P (121 MHz) 7.9 (qm, $^2J_{PF}$ 91); δ_F (282 MHz) -73.8 (d, $^2J_{FP}$ 91).

3-Amino-3-[hydroxy(trifluoromethyl)phosphoryl)propanoic acid (**41**). A mixture of **40** (0.50 g, 1.9 mmol) and 5N HCl (5 mL) was stirred in an ampoule with Teflon stopcock at 130 °C for 5 h. The resulting solution was evaporated under reduced pressure and co-evaporated with water to the dryness. The crude product was triturated with acetone, dissolved in water and passed down an ion-exchange column to give **41** as a white powder (0.18 g, 43%), mp 210°C (dec.). [Found: C, 21.67; H, 3.09; N. 6.32. $C_4H_7F_3NO_4P$ requires C, 21.73; H, 3.19; N, 6.34 %]; NMR (D_2O): δ_H (500 MHz): 2.81 (1H, ddd, J_{AB} 18.1, $^3J_{HP}$ 10.0, $^3J_{HH}$ 8.1, CH_2), 2.97 (1H, ddd, J_{AB} 18.1, $^3J_{HP}$ 12.5, $^3J_{HH}$ 4.5, CH_2), 3.84-3.92 (1H, m, PC*H*); δ_P (121 MHz) 12.4 (qm, $^2J_{PF}$ 93); δ_F (282 MHz) -74.3 (d, $^2J_{FP}$ 93); δ_C (125 MHz) 31.8 (CH_2), 44.1 (d, $^1J_{CP}$ 105, P*C*H), 122.2 (qd, $^1J_{CF}$ 316, $^1J_{CP}$ 179, CF_3), 173.7 (*C*OOH).

References

1. Vidal, J.; Guy, L.; Sterin, S.; Collet, A. *J. Org. Chem.* **1993**, *58*, 4791 - 4793.
2. Jung, M. E.; Shishido, K.; Light, L.; Davis, L. *Tetrahedron Lett.* **1981**, 4607 - 4610.
3. Poisel, H.; Schmidt, U. *Chem. Ber.* **1975**, *108*, 2547 - 2553.
4. Ramapanicker, R.; Kavita, De; Srinivasan, Ch. *Tetrahedron* **2007**, *63*, 10534-10542.
5. Golovanov, A. V.; Maslennikov, I. G.; Lavrent`ev, A.N. *Zh. Obsch. Khim.* **1988**, *58*, 1525 – 1529; *J. Gen. Chem. USSR* **1988**, *58*, 1357 – 1360;. *Chem Abstr*. **1988**, *108*, 21973h.
