# Supplementary material for: A novel family of (1-aminoalkyl)(trifluoromethyl)- and -(difluoromethyl)phosphinic acids – analogues of α-amino acids
Source: Beilstein J Org Chem. 2014 Mar 26;10:722–31. doi: 10.3762/bjoc.10.66 (PMC3999843; doi:10.3762/bjoc.10.66)

Supporting Information File 2
for

A novel family of (1-aminoalkyl)(trifluoromethyl)- and -(difluoromethyl)phosphinic acids – analogues of α-amino acids

Natalia V. Pavlenko[1], Tatiana I. Oos[1], Yurii L. Yagupolskii*[1], Igor I. Gerus[2], Uwe Doeller[3] and Lothar Willms[3]

Address: [1]Institute of Organic Chemistry National Academy of Sciences of Ukraine, Murmanskaya str. 5, 02660 Kiev-94, Ukraine, [2]Institute of Bioorganic Chemistry and Petrochemistry National Academy of Sciences of Ukraine, Murmanskaya str. 1, 02660 Kiev-94, Ukraine and [3]Bayer CropScience Aktiengesellschaft BCS AG-R-WC-WCC-C2 Weed Control Chemistry 2, Frankfurt, G836, 101, Germany

Email: Yurii L. Yagupolskii - Yagupolskii@ioch.kiev.ua

*Corresponding author

NMR spectra of the most typical compounds.

(Dibenzylamino)methyl](trifluoromethyl)phosphinic acid (**13a**).

^1H (300 MHz), DMSO-d_6

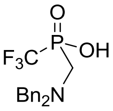

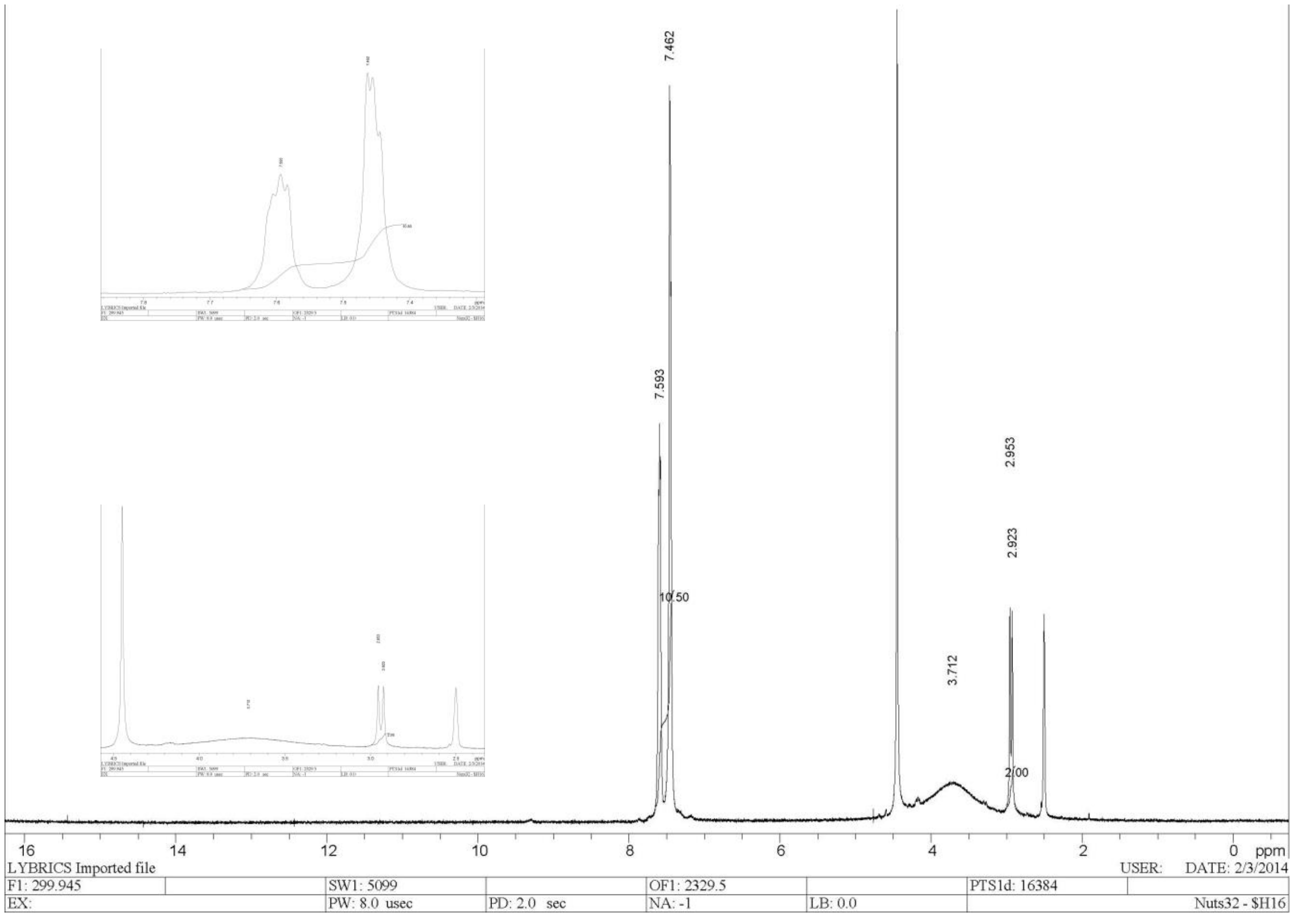

^{31}P (121 MHz), DMSO-d_6

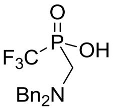

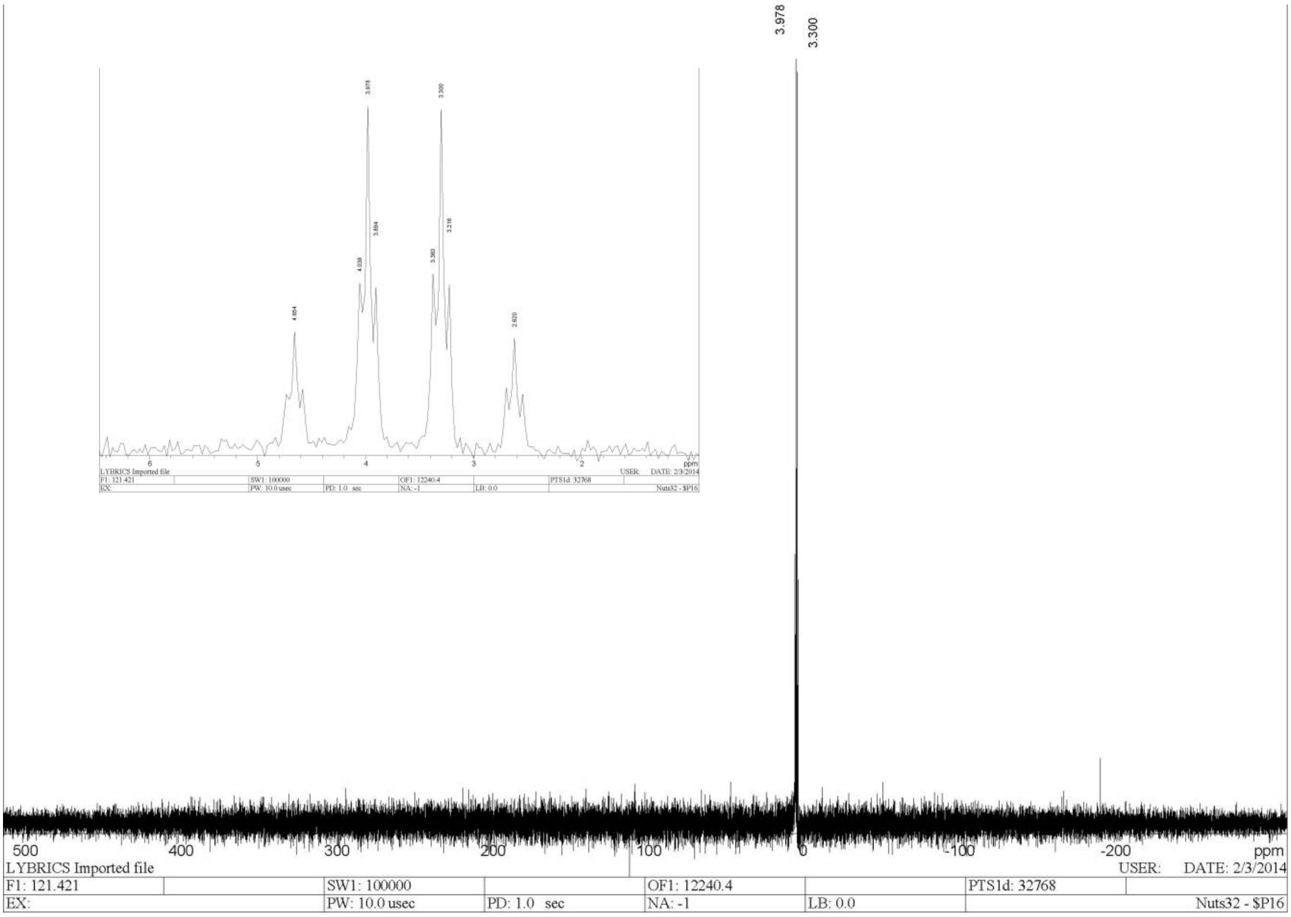

^{19}F (188 MHz), DMSO-d_6

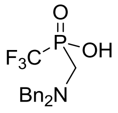

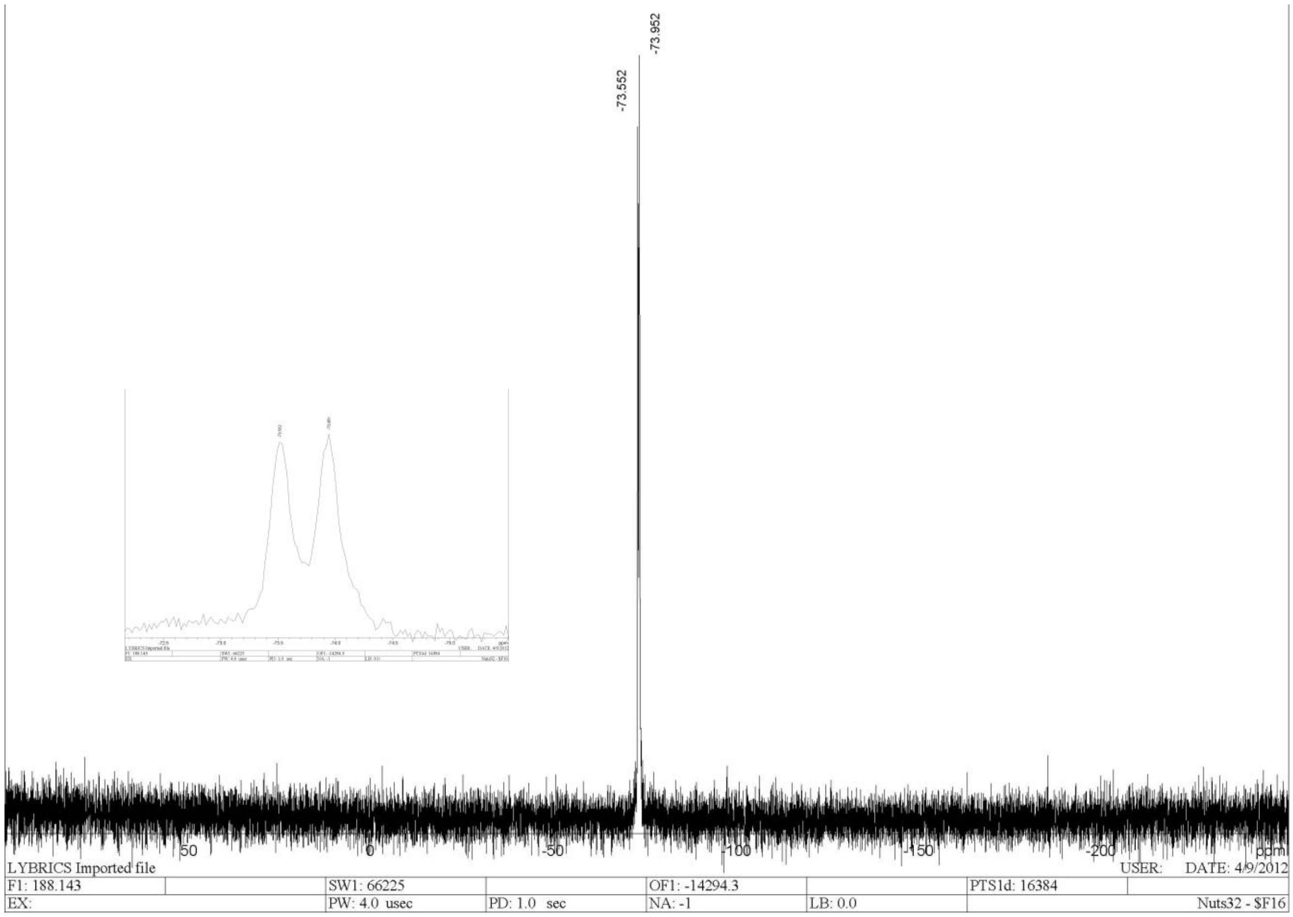

[1-(Dibenzylamino)ethyl](trifluoromethyl)phosphinic acid (**13c**) in a mixture with $Bn_2NH{\cdot}HCl$ (~1:7).

^1H (300 MHz), DMSO-d_6

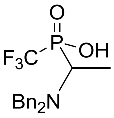

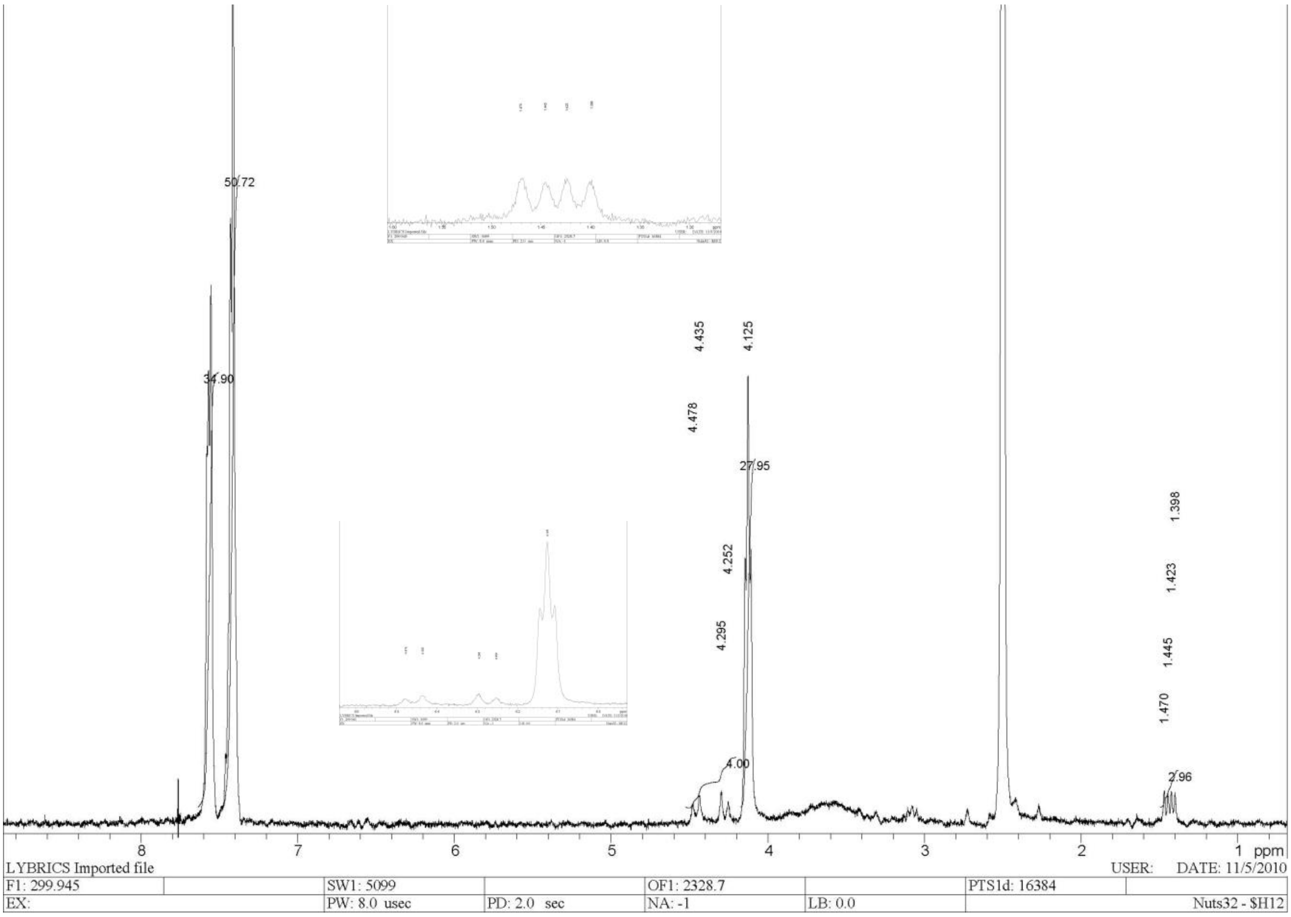

(Aminomethyl)(trifluoromethyl)phosphinic acid (**14a**) (Table 1, entry 1).

^{1}H (500 MHz), D_2O

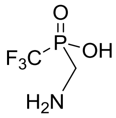

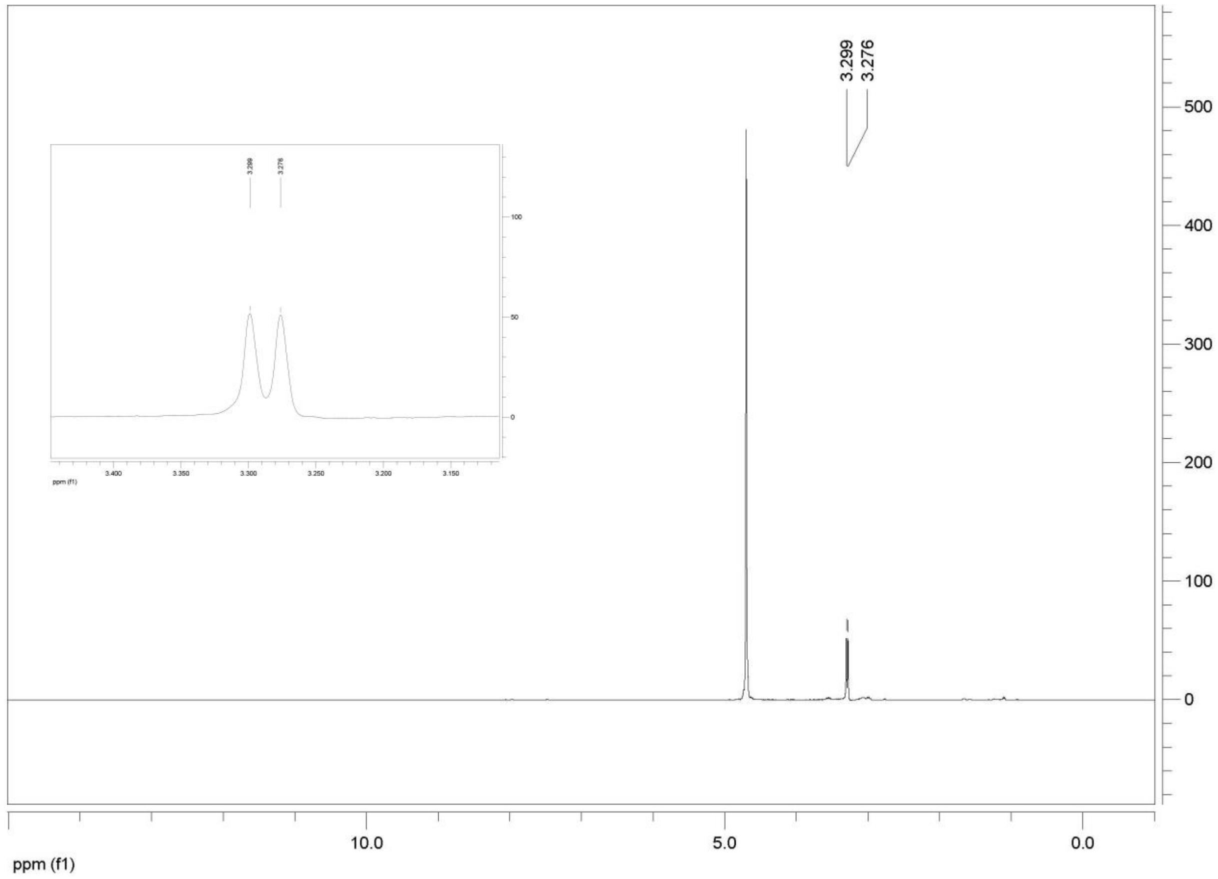

^{13}C (125 MHz), D_2O

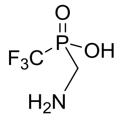

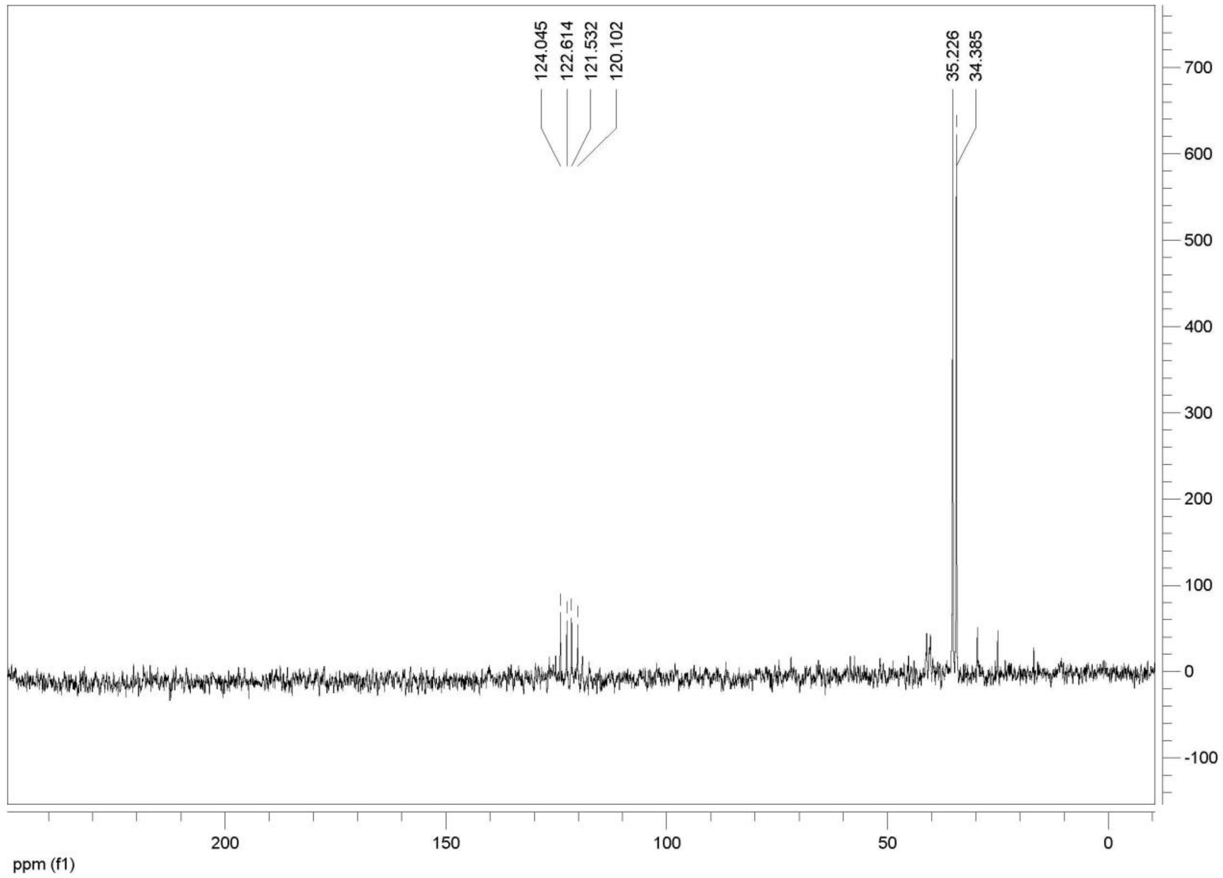

^{31}P (81 MHz), D_2O

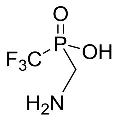

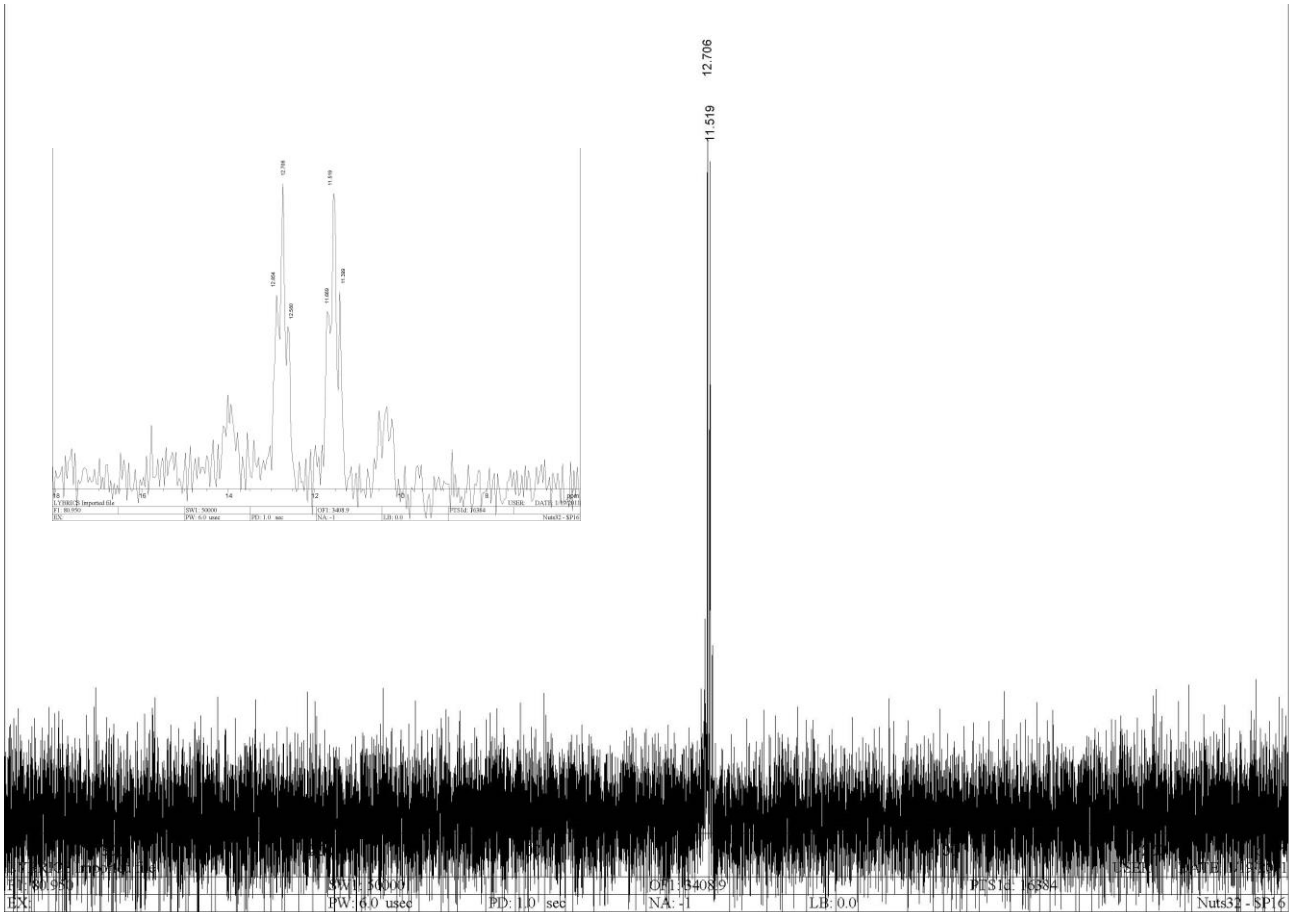

(1-Amino-2-methylpropyl)(trifluoromethyl)phosphinic acid (**14d**) (Table 1, entry 4).

^{1}H (500 MHz), D_2O

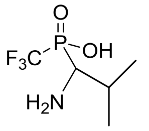

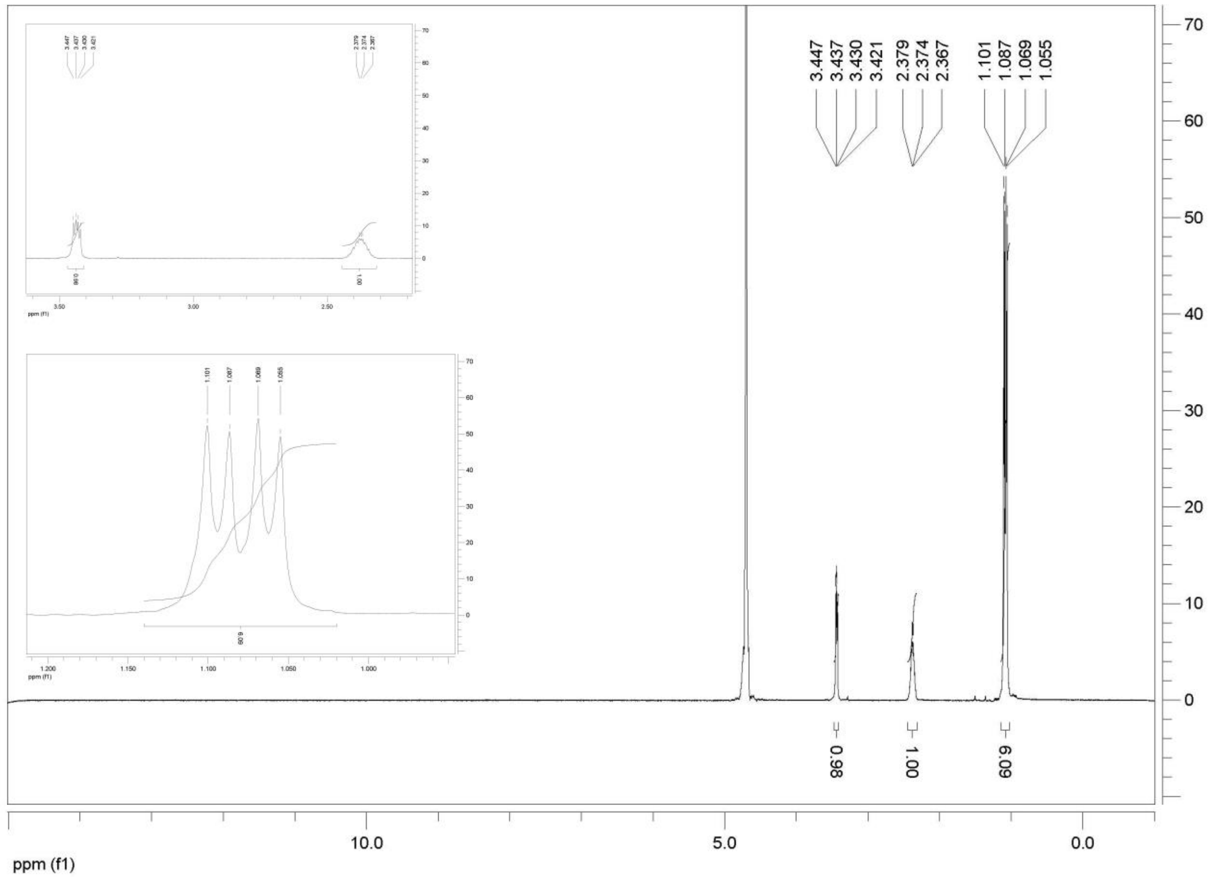

^{13}C (125 MHz), D_2O

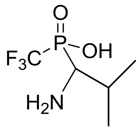

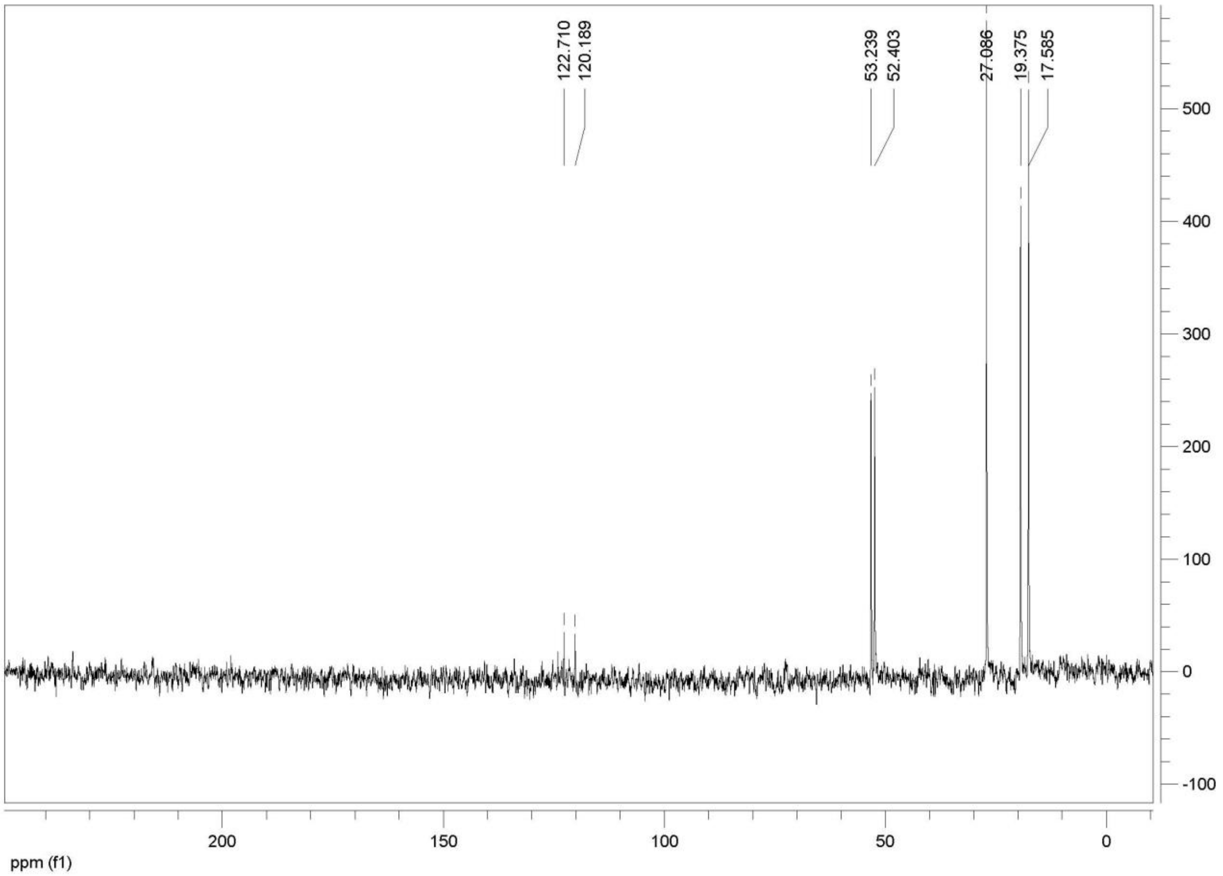

^{31}P (81 MHz), D_2O

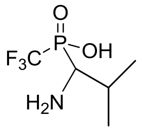

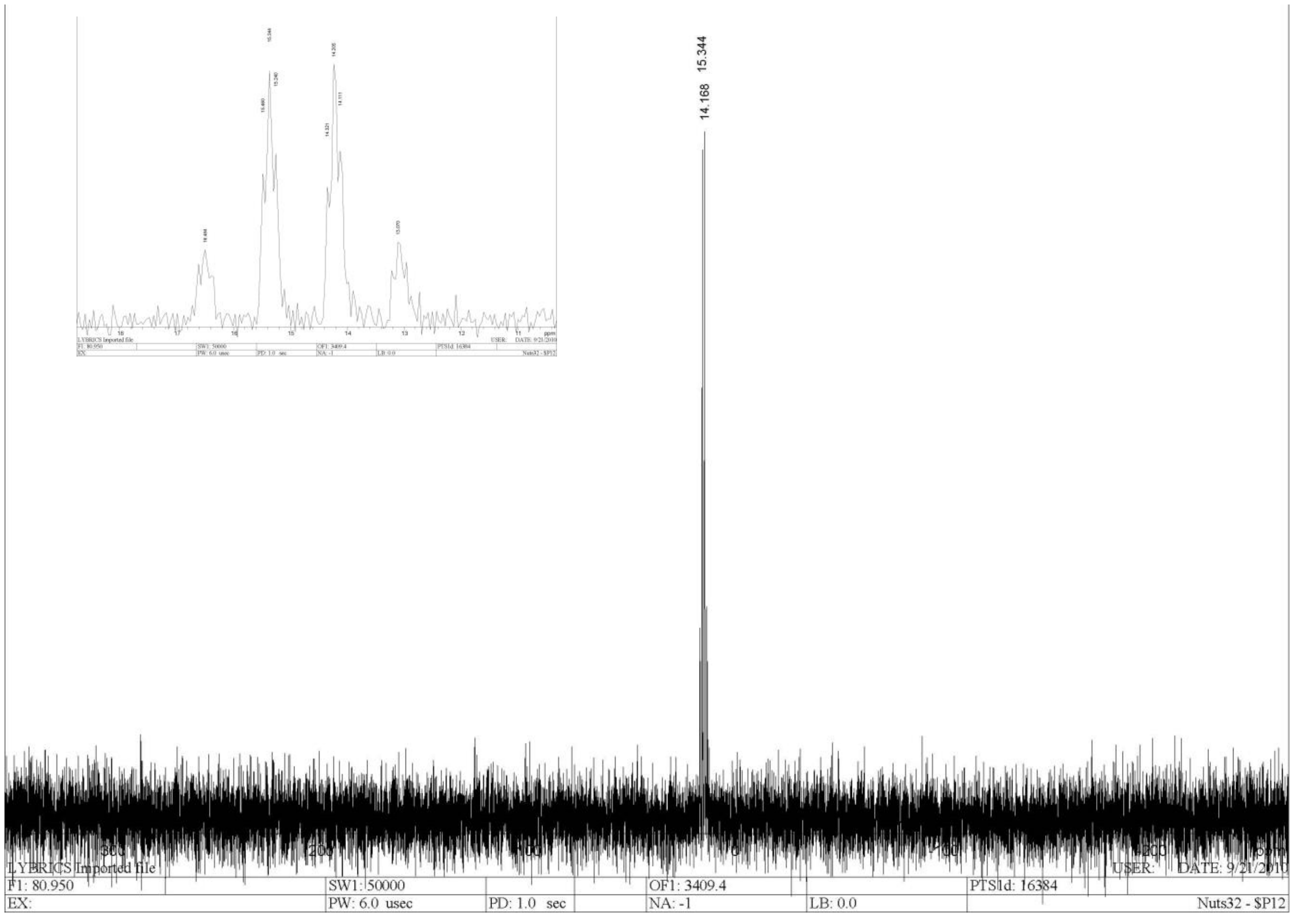

^{19}F (188 MHz), D_2O

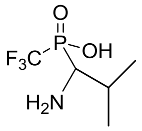

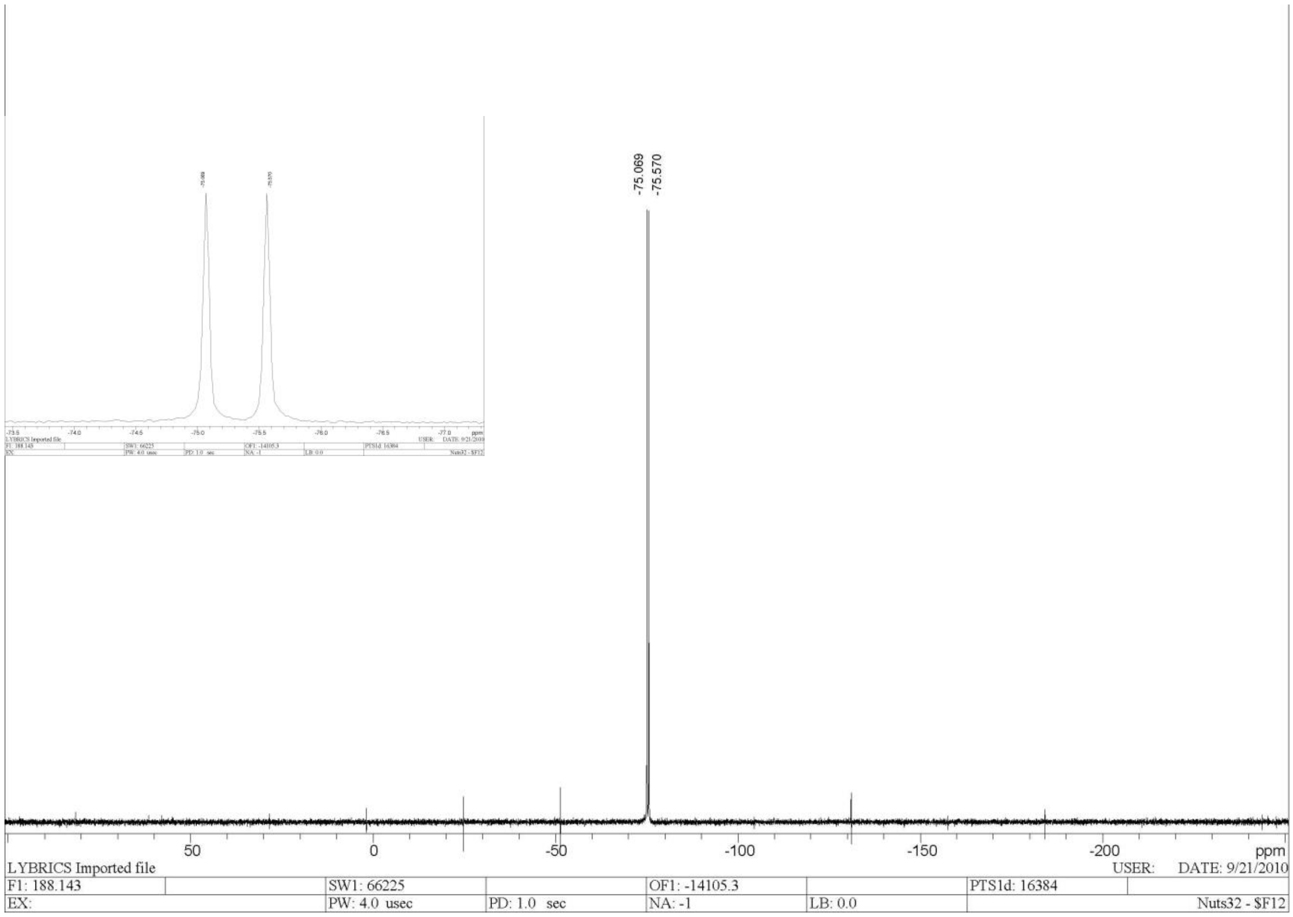

Pyrrolidin-2-yl-(trifluoromethyl)phosphinic acid (**14e**) (Table 1, entry 5).

^{1}H (500 MHz), D_2O

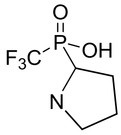

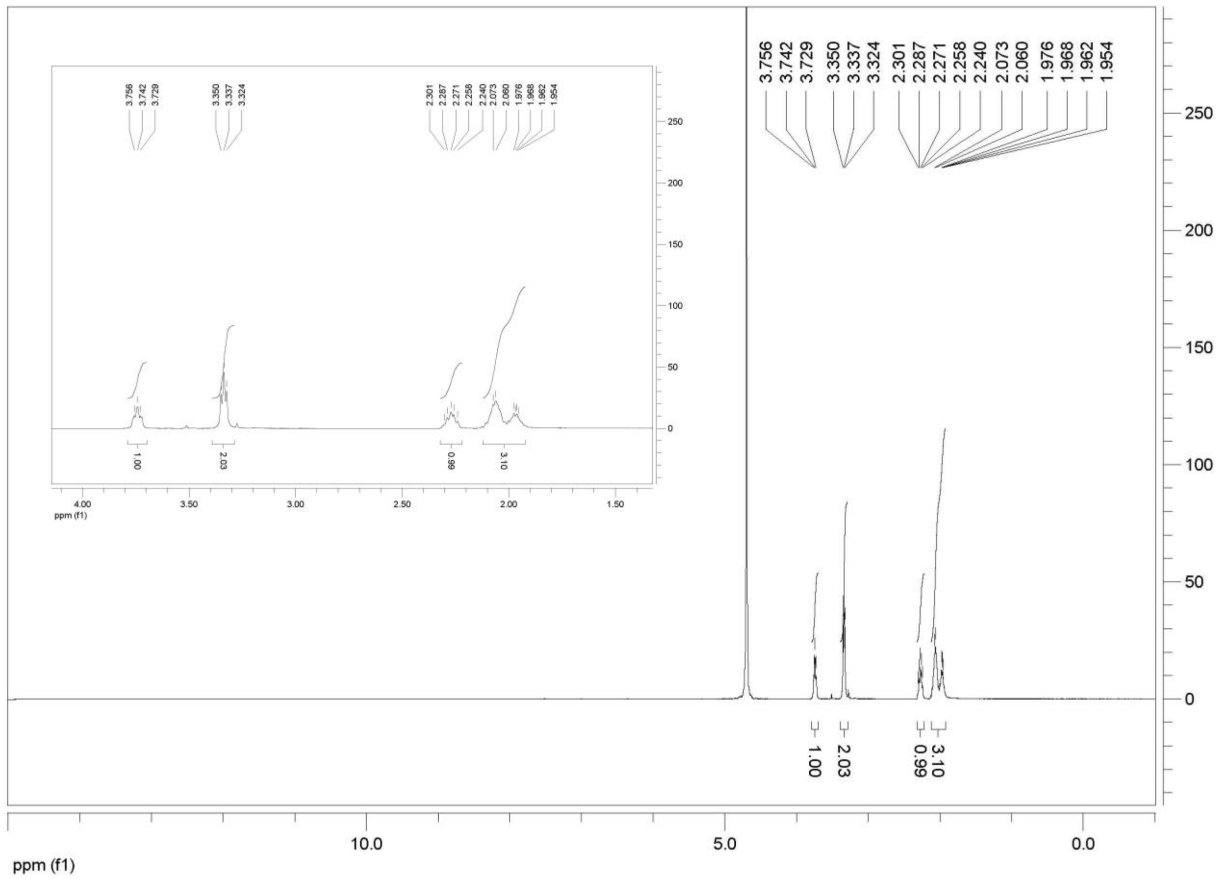

^{13}C (125 MHz), D_2O

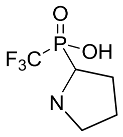

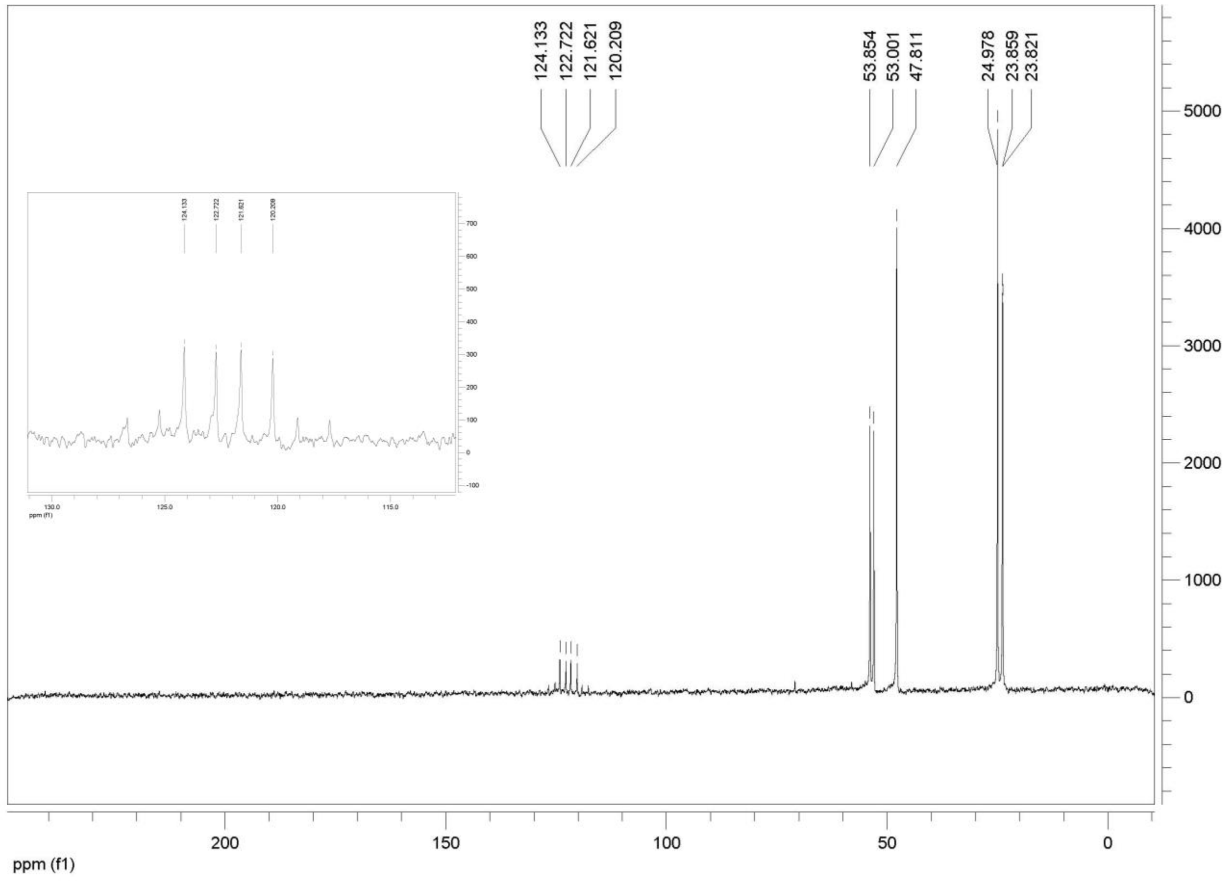

^{31}P (81 MHz), D_2O

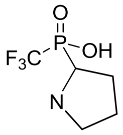

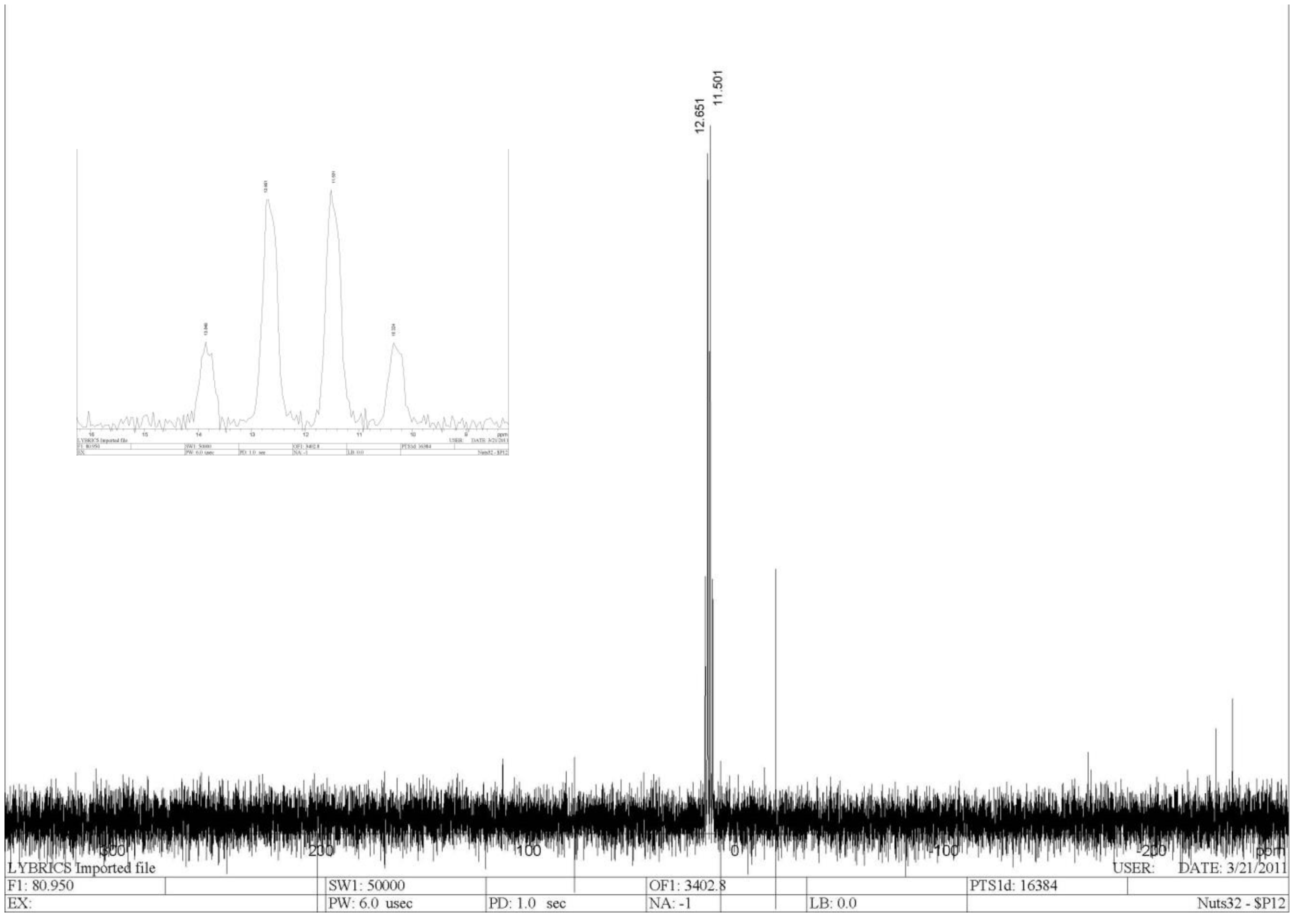

^{19}F (188 MHz), D_2O

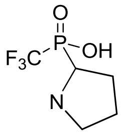

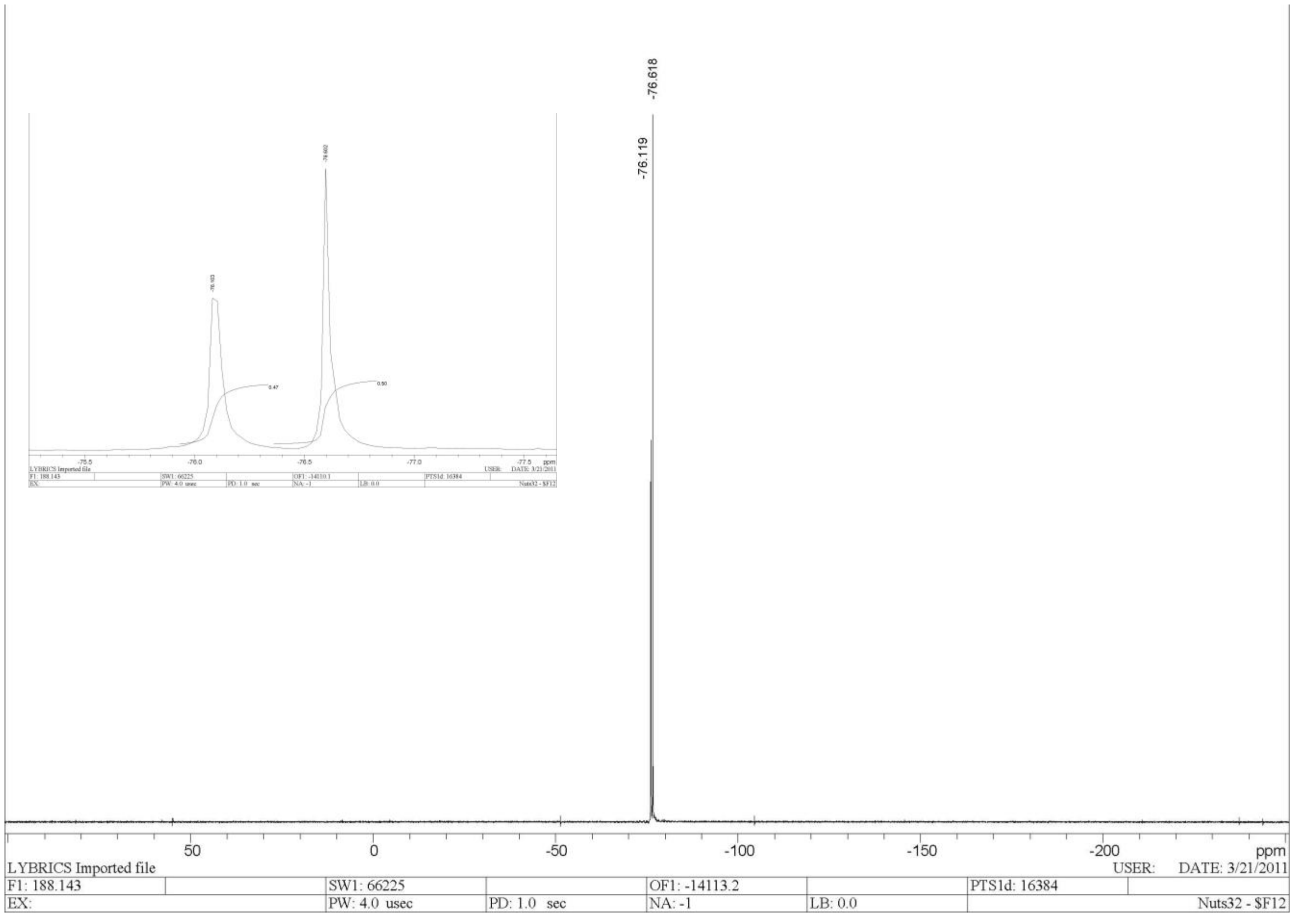

[1-(Benzylamino)-2-methylpropyl](trifluoromethyl)phosphinic acid (**17d**) (Table 1, entry 4).

^1H (300 MHz), DMSO-d_6

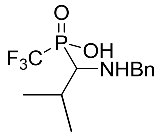

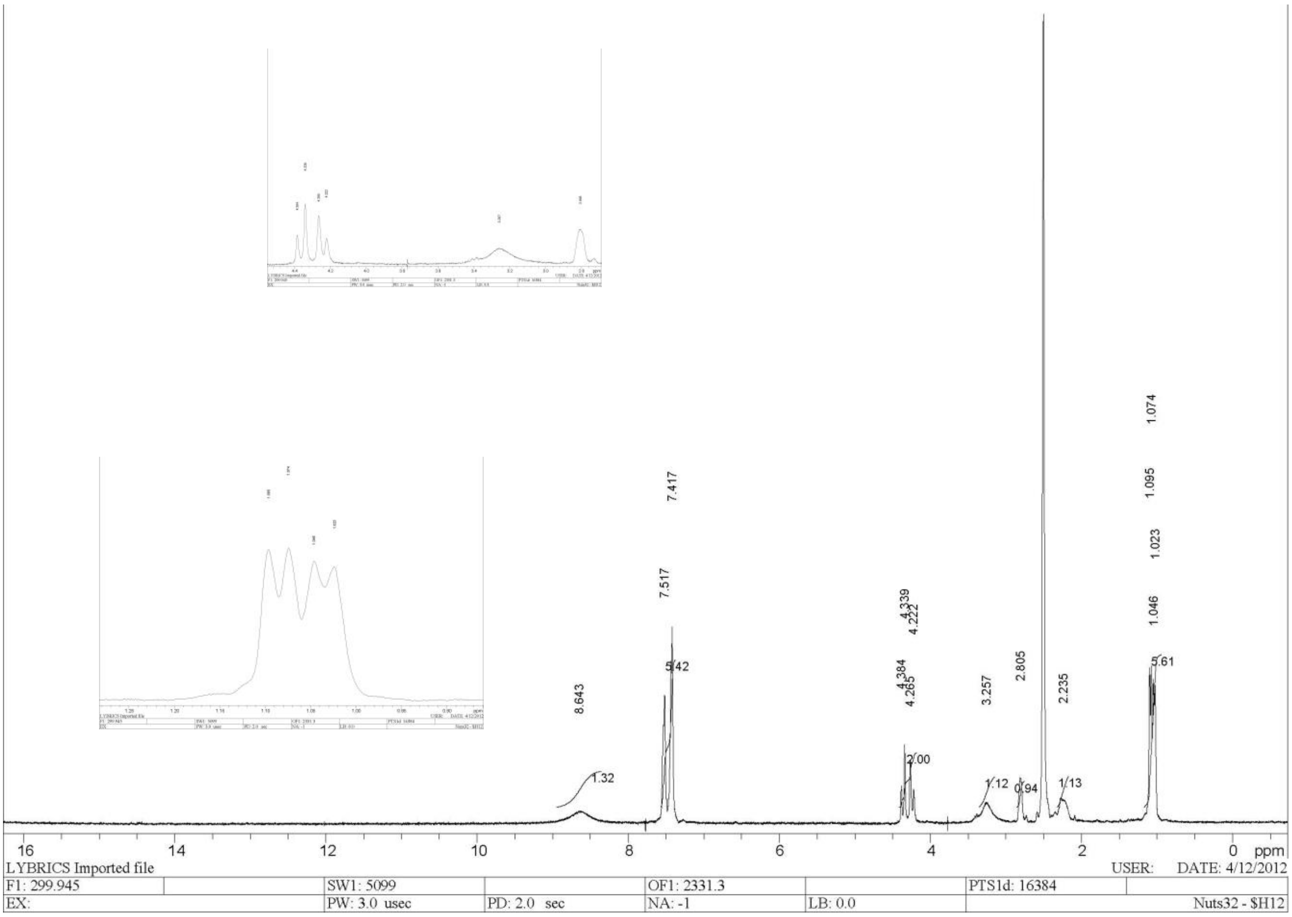

^{19}F (188 MHz), DMSO-d_6

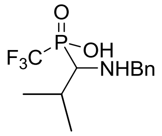

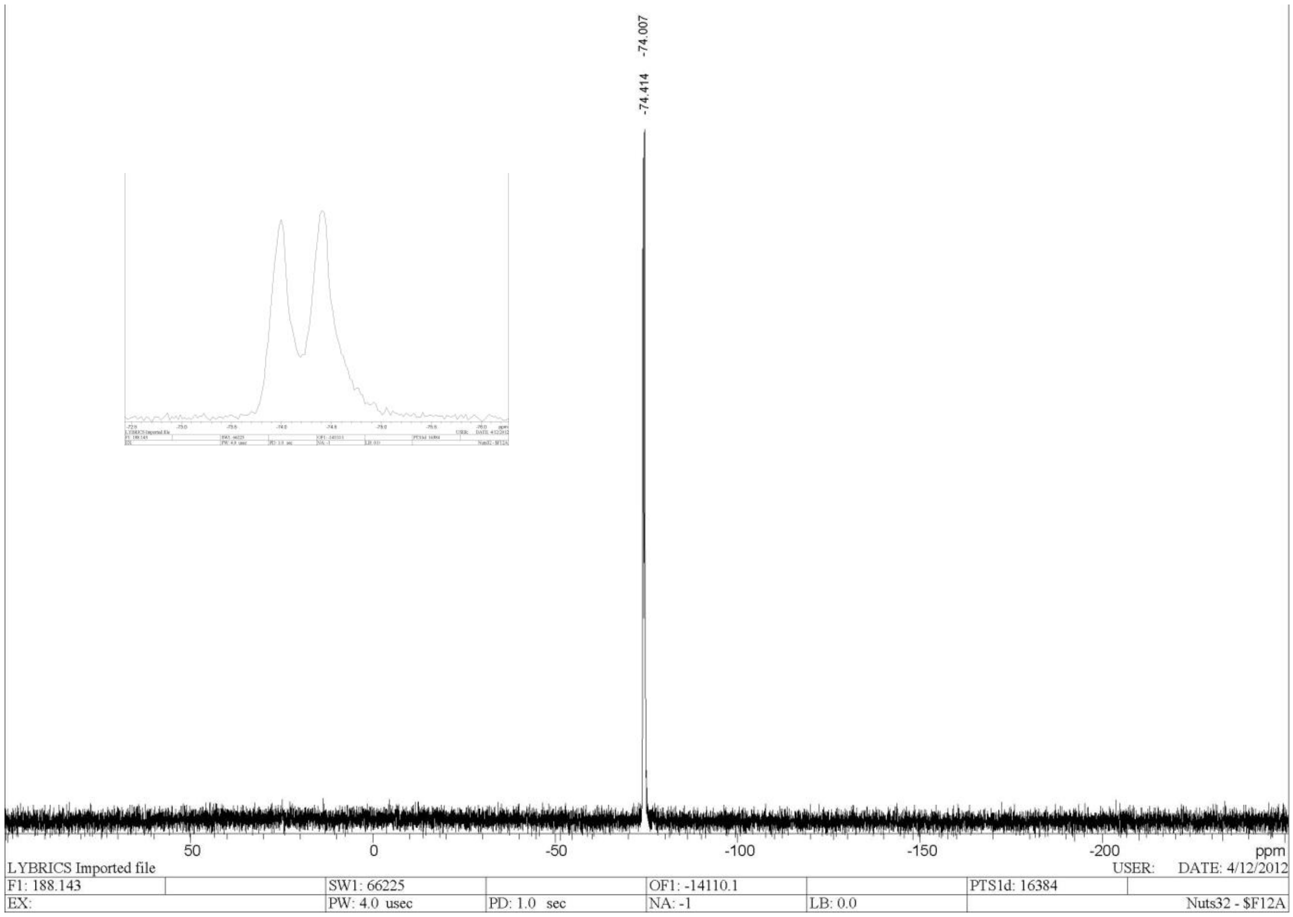

^{31}P (121 MHz), DMSO-d_6

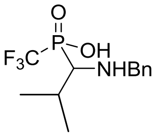

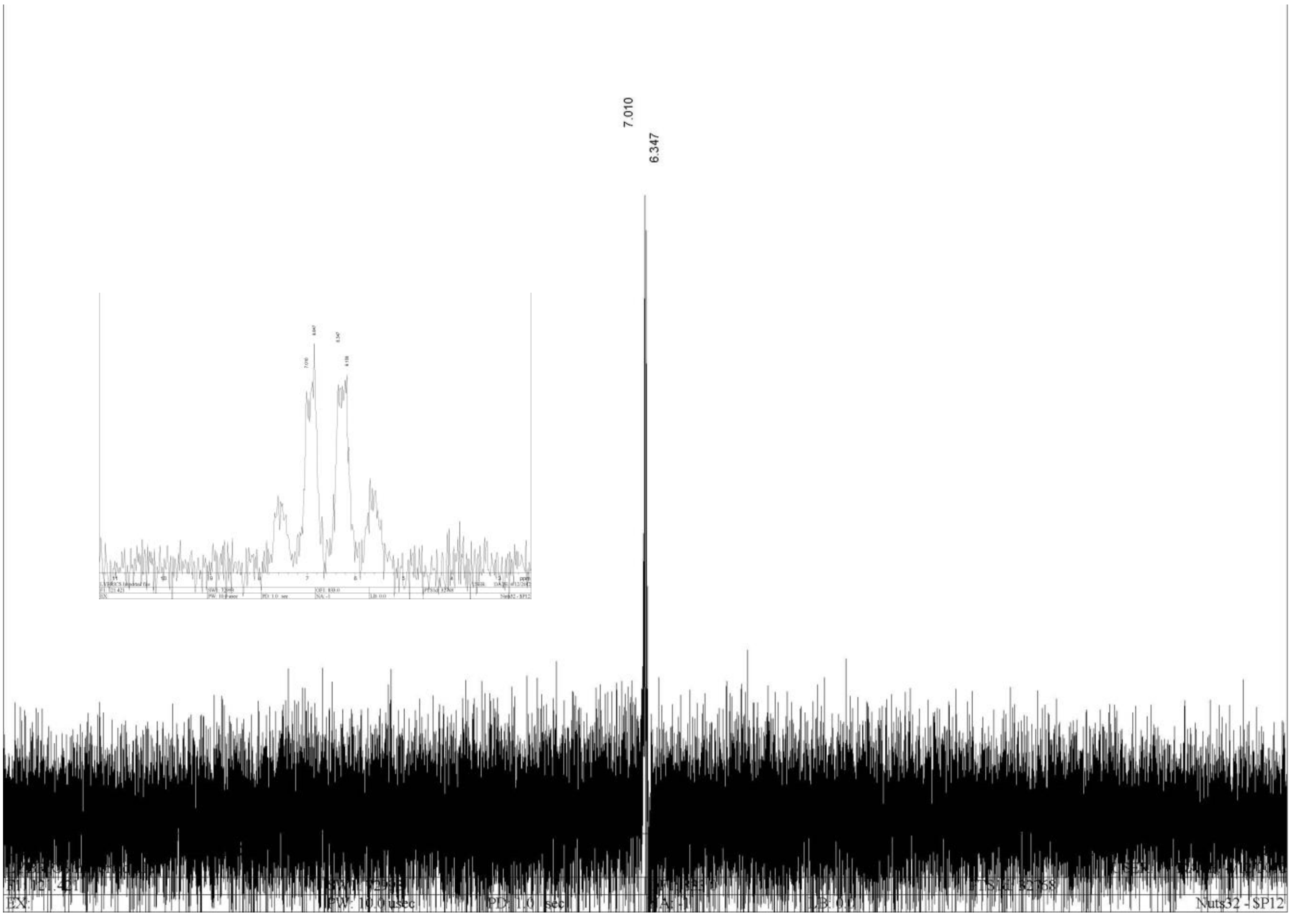

Ethyl [(benzylamino)(phenyl)methyl](difluoromethyl)phosphinate (**18b**) (Table 2, entry 2).

^{1}H (300 MHz), $CDCl_3$

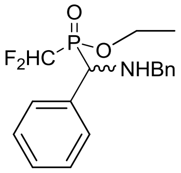

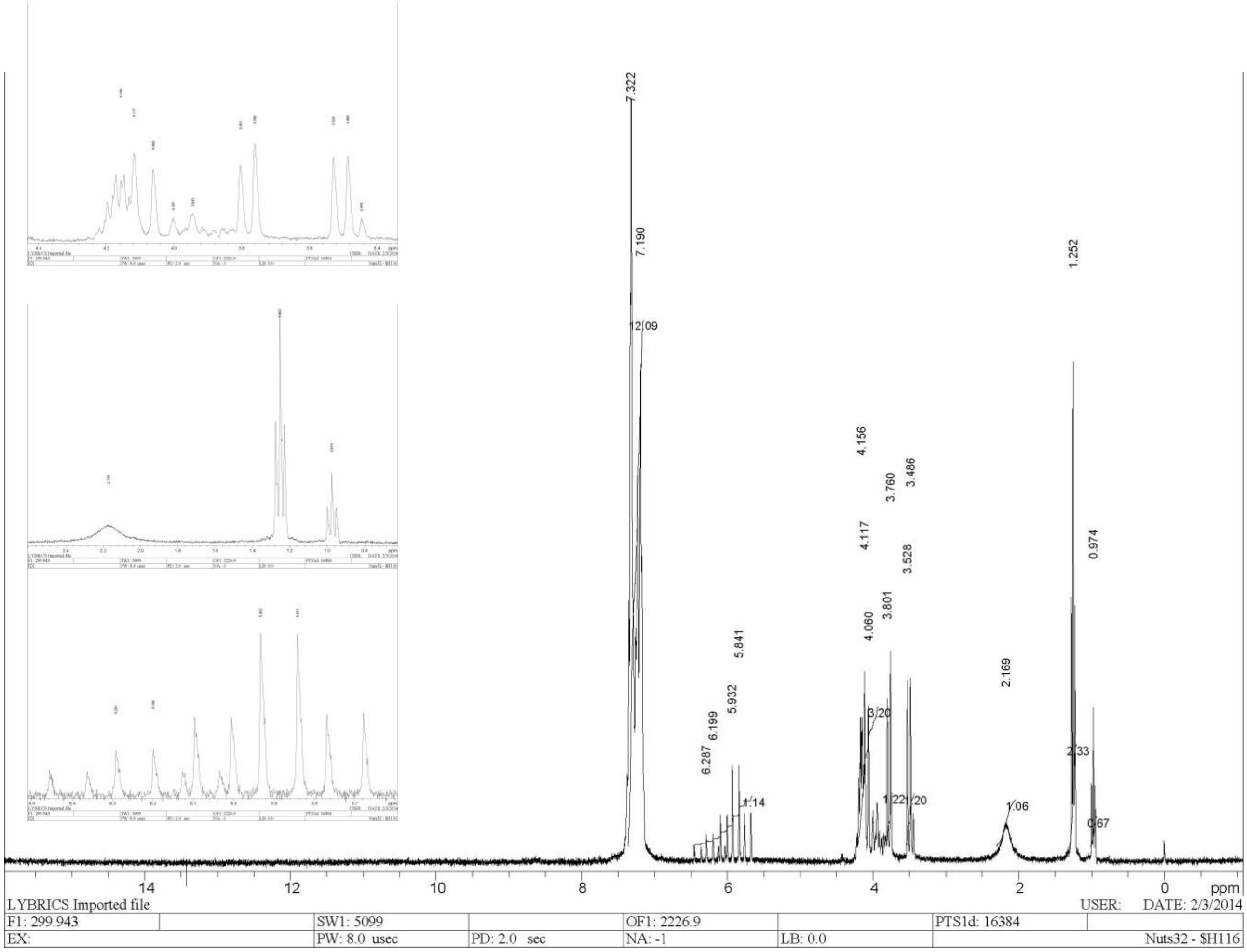

Ethyl [1-(benzylamino)ethyl](difluoromethyl)phosphinate (**18c**) (Table 2, entry 3).

^{19}F (188 MHz), D_2O

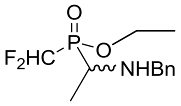

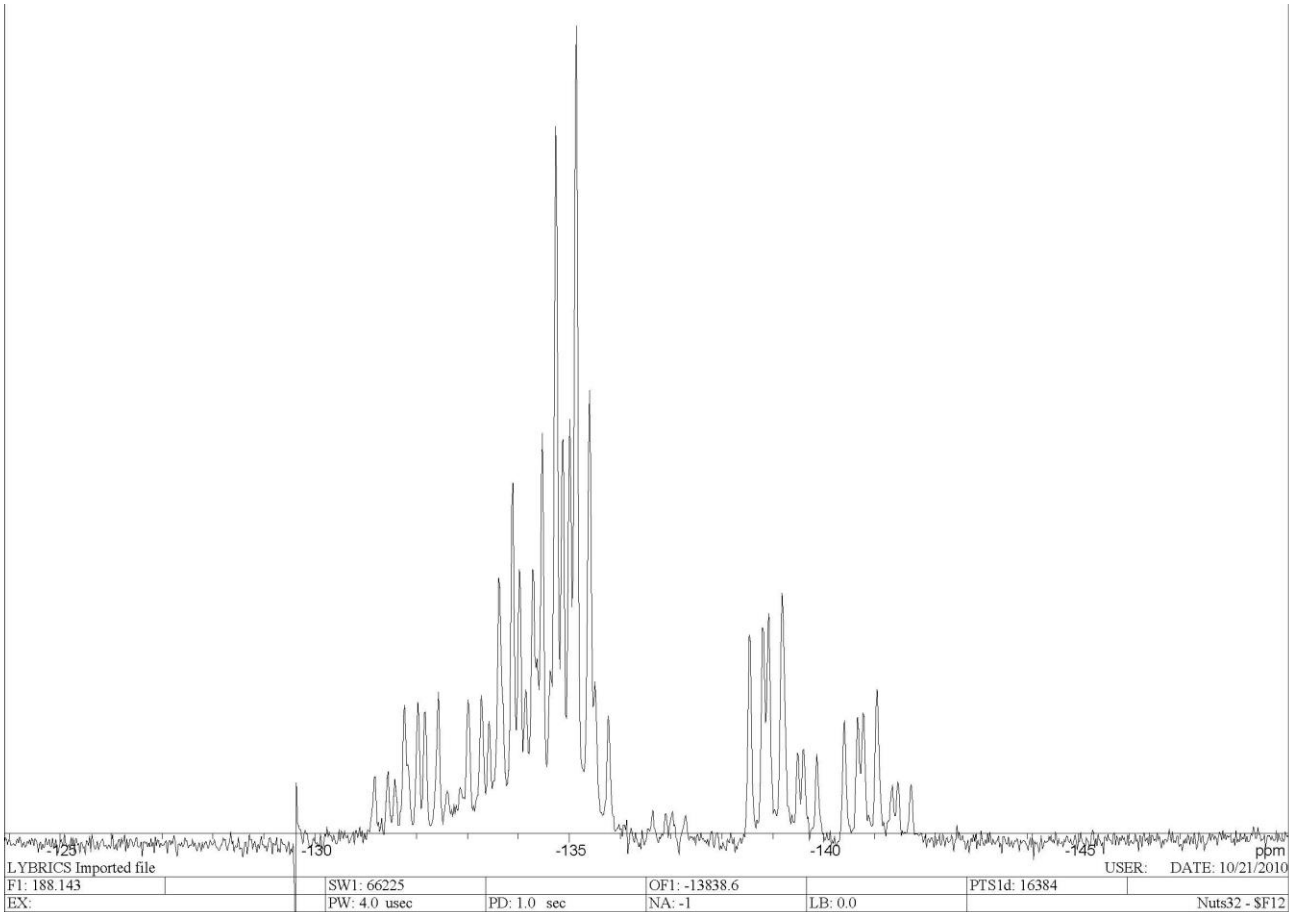

[Amino(phenyl)methyl](difluoromethyl)phosphinic acid (**20b**) (Table 2, entry 2).

^1H (500 MHz), D_2O

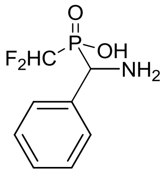

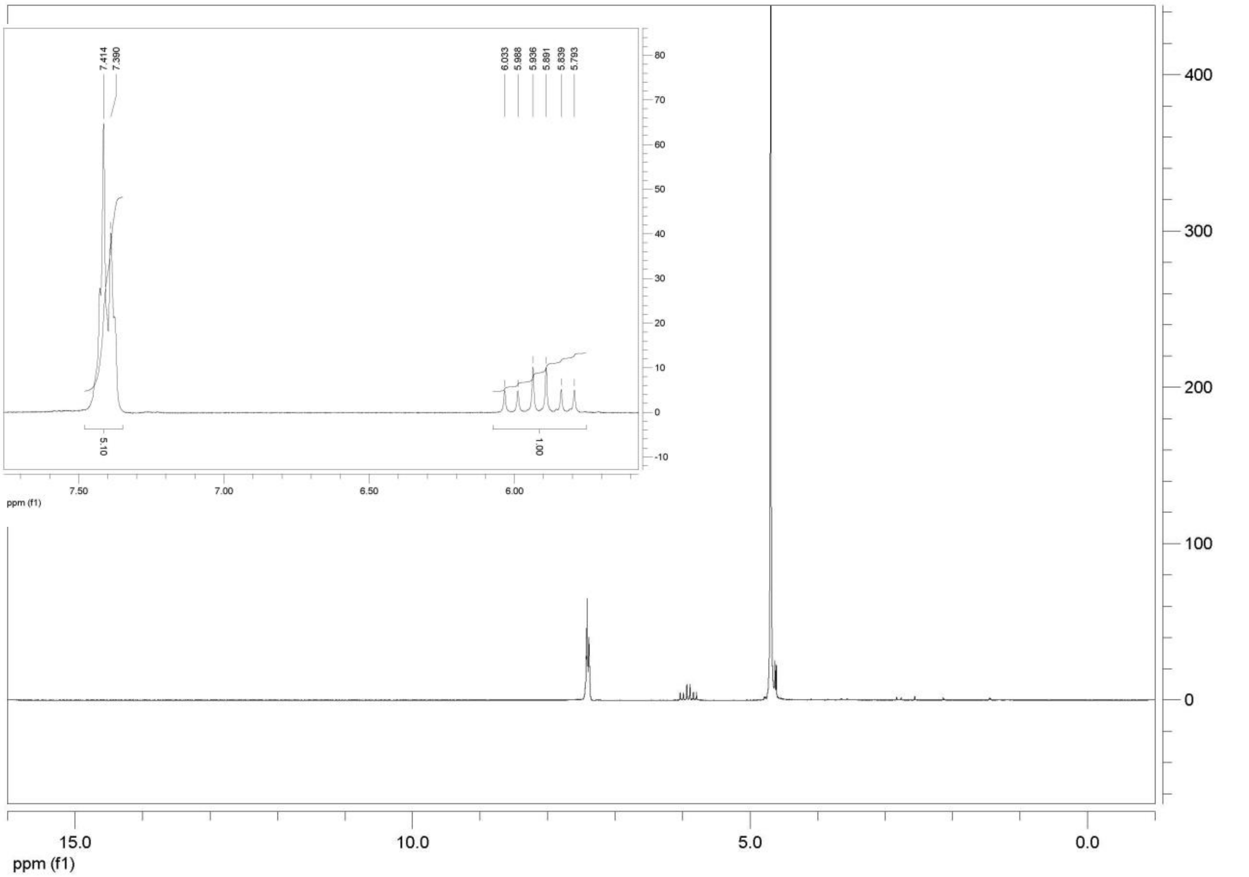

^{1}H (300 MHz), D_2O

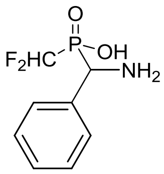

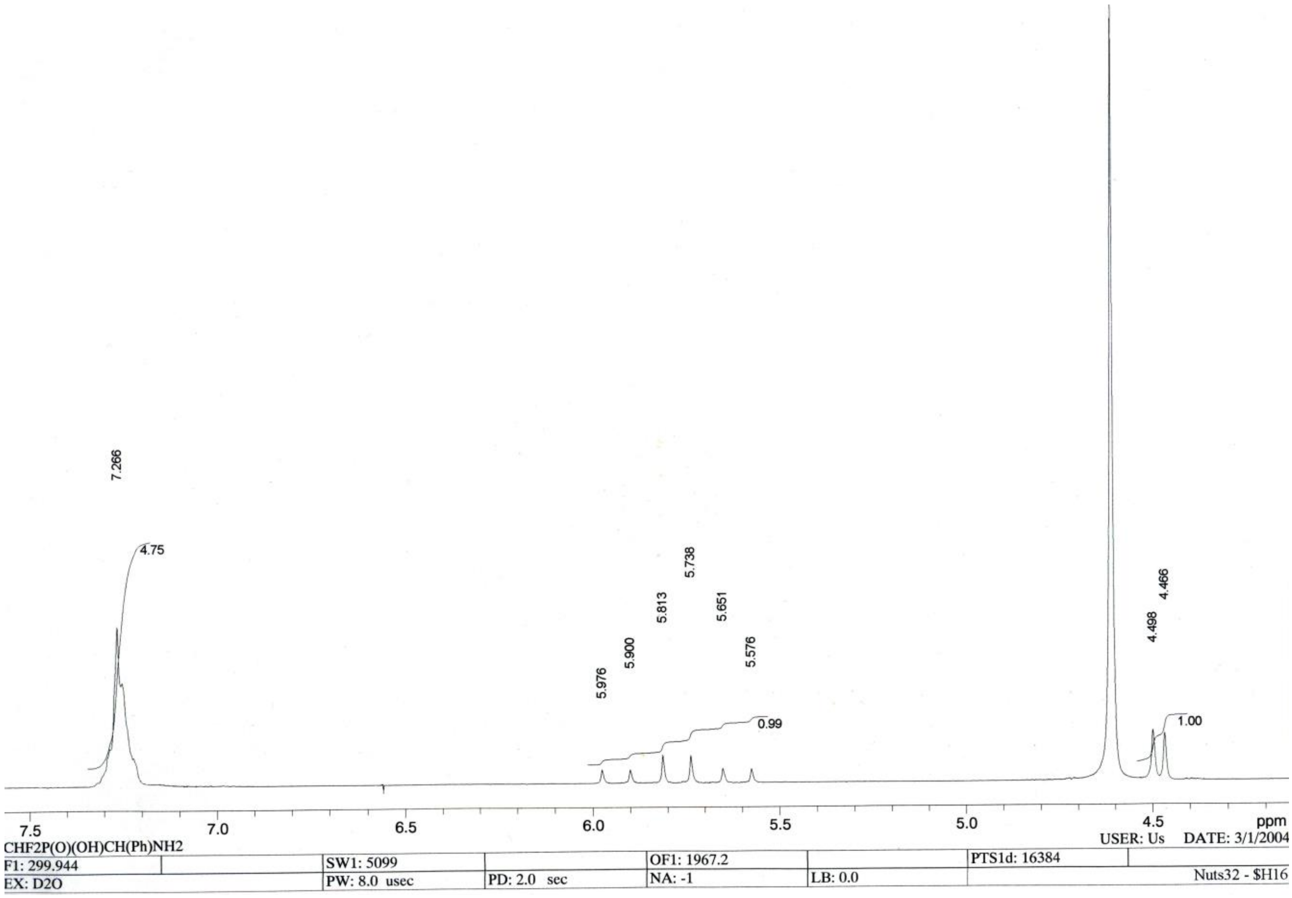

^{13}C (125 MHz), D_2O

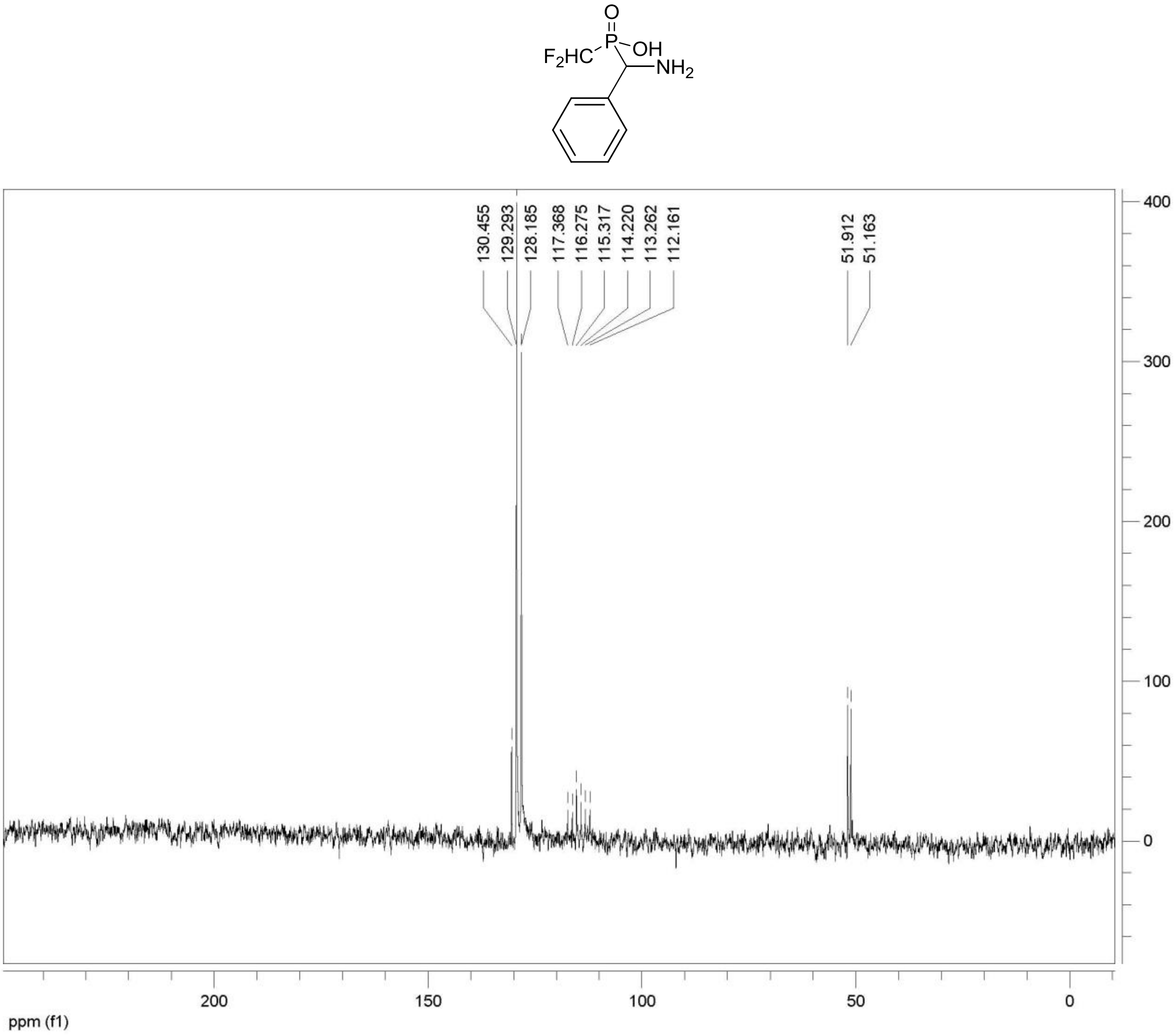

^{19}F (376 MHz), D_2O

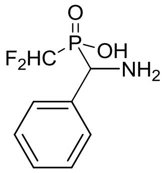

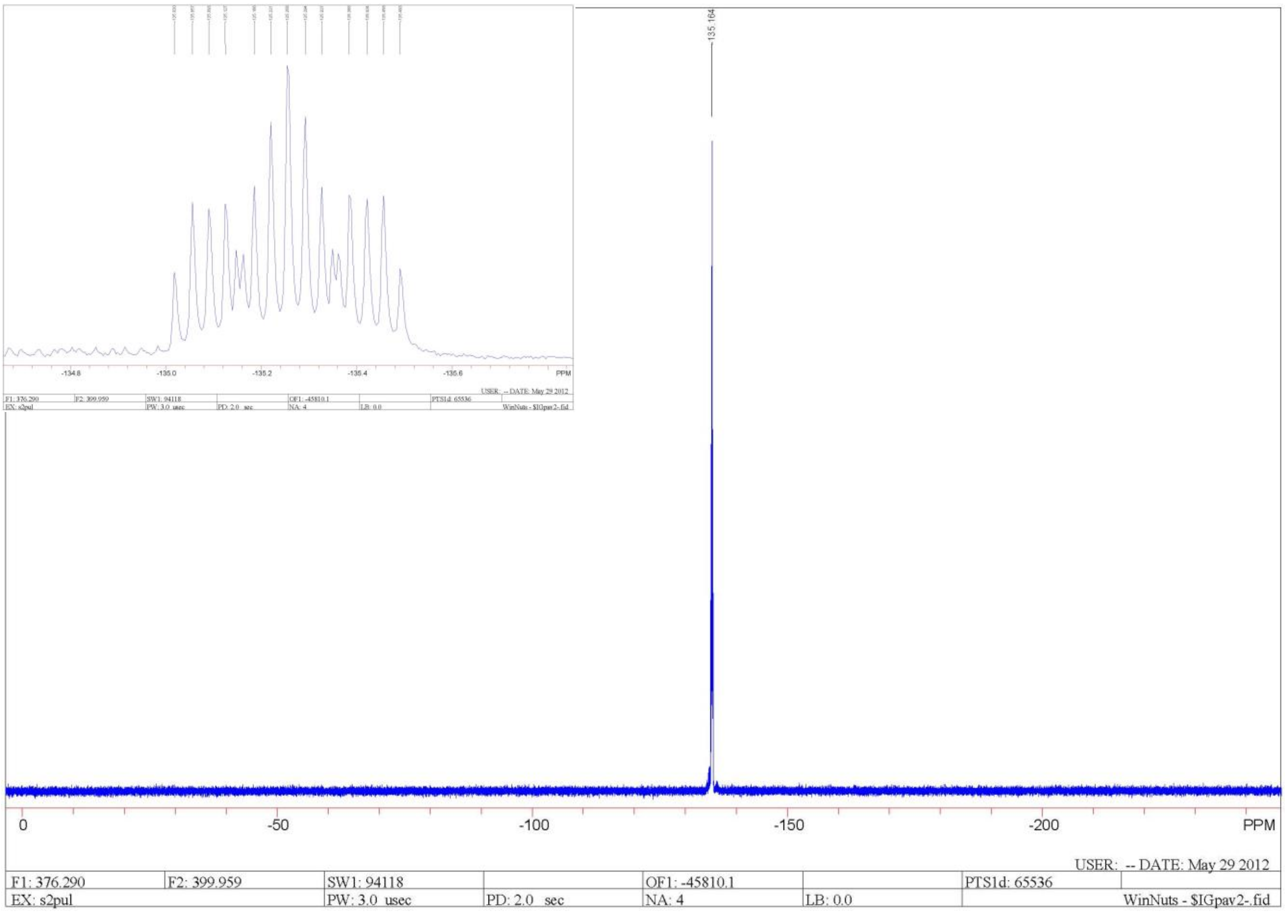

(1-Aminoethyl)(difluoromethyl)phosphinic acid (**20c**) (Table 2, entry 3).

^{1}H (500 MHz), D_2O

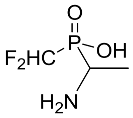

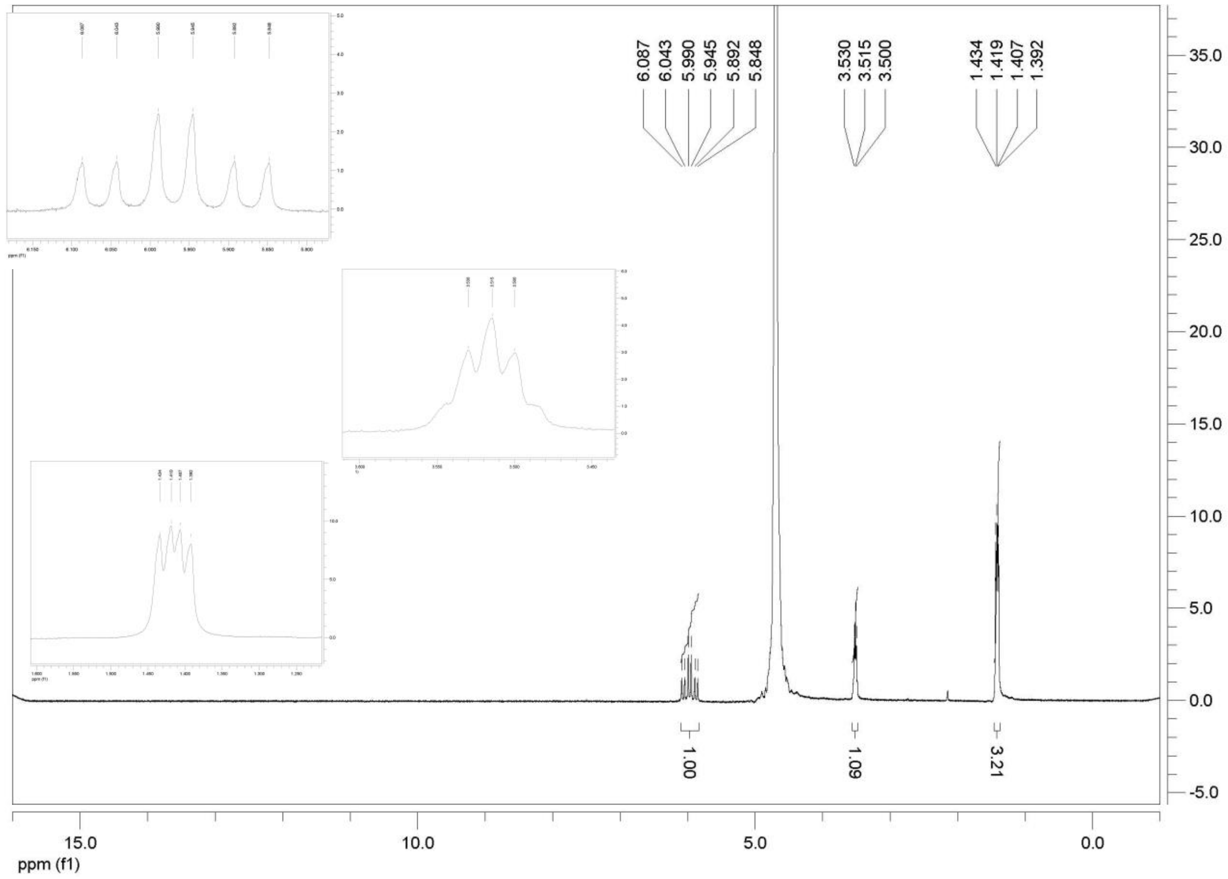

^{13}C (125 MHz), D_2O

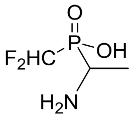

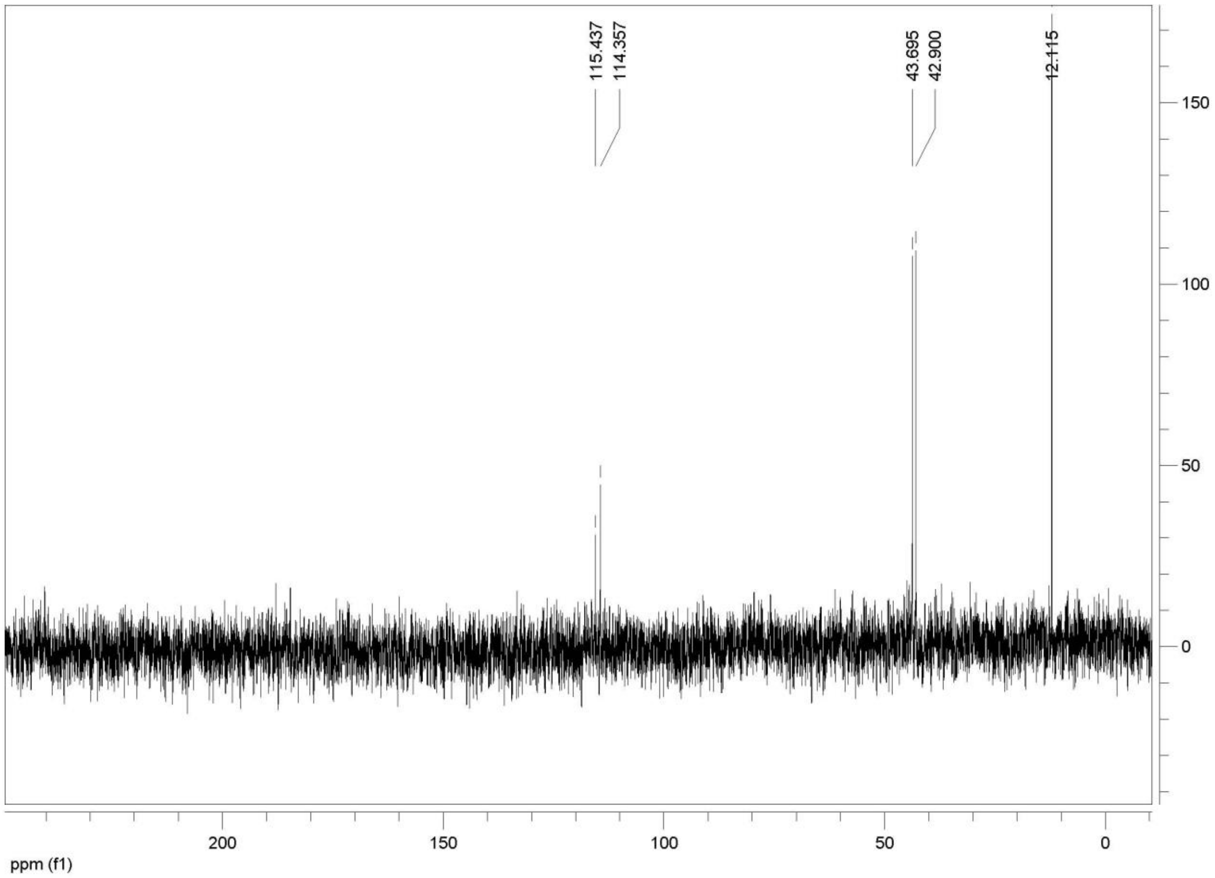

^{19}F (376 MHz), D_2O

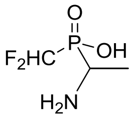

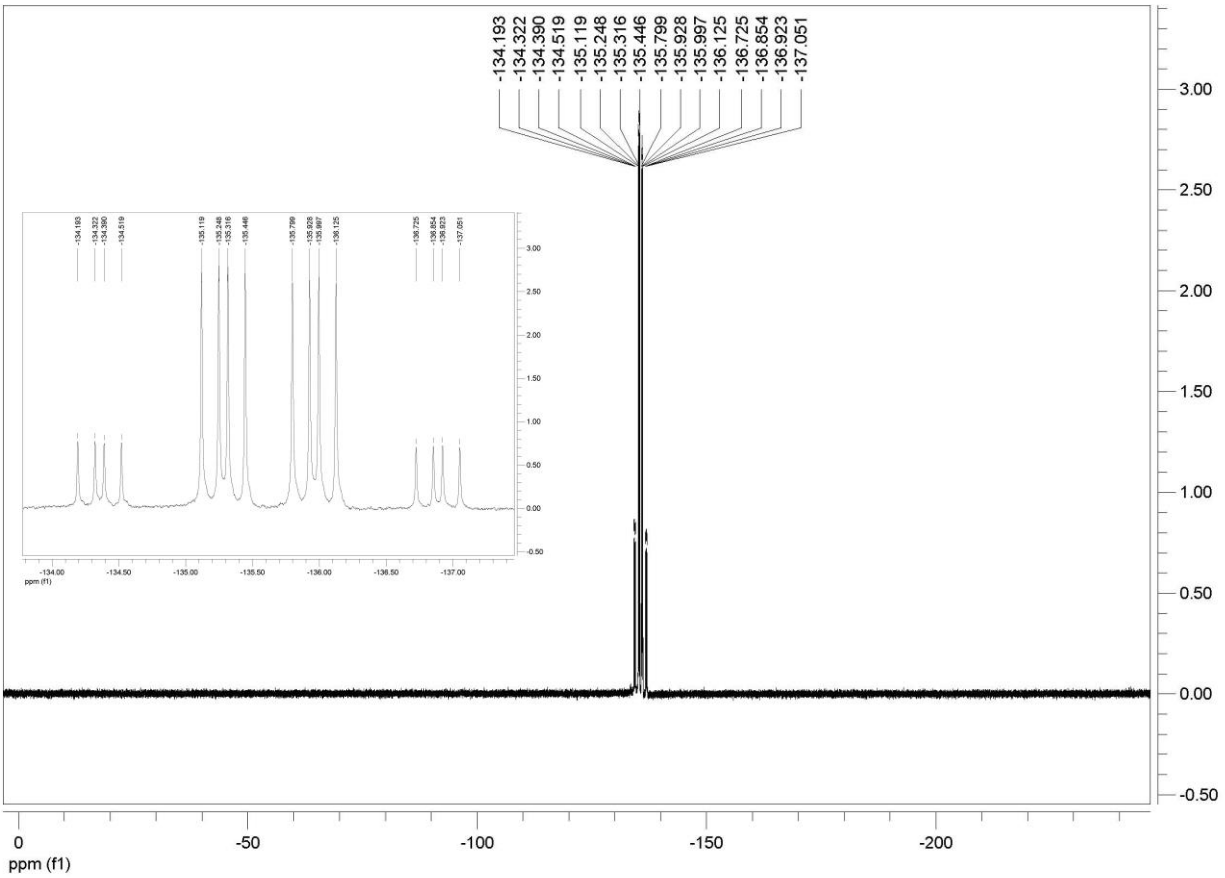

^{31}P (121 MHz), D_2O

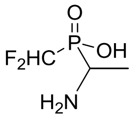

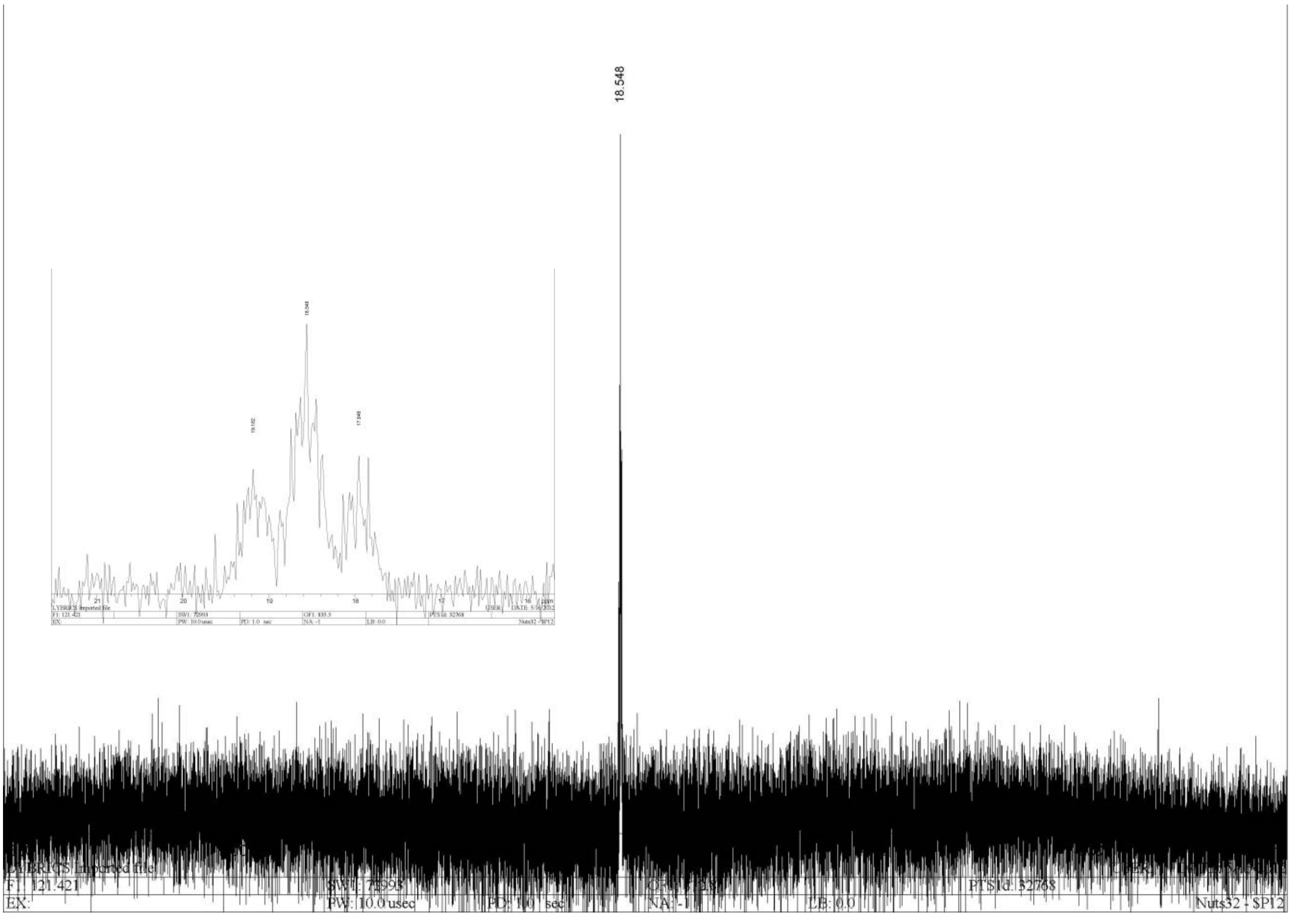

Supplement: File 2 — NMR spectra of the most typical compounds. [file Beilstein_J_Org_Chem-10-722-s002.pdf]
