# Supplementary material for: A novel family of (1-aminoalkyl)(trifluoromethyl)- and -(difluoromethyl)phosphinic acids – analogues of α-amino acids
Source: Beilstein J Org Chem. 2014 Mar 26;10:722–31. doi: 10.3762/bjoc.10.66 (PMC3999843; doi:10.3762/bjoc.10.66)

Supporting Information File 3
for

A novel family of (1-aminoalkyl)(trifluoromethyl)- and -(difluoromethyl)phosphinic acids – analogues of α-amino acids

Natalia V. Pavlenko[1], Tatiana I. Oos[1], Yurii L. Yagupolskii*[1], Igor I. Gerus[2], Uwe Doeller[3] and Lothar Willms[3]

Address: [1]Institute of Organic Chemistry National Academy of Sciences of Ukraine, Murmanskaya str. 5, 02660 Kiev-94, Ukraine, [2]Institute of Bioorganic Chemistry and Petrochemistry National Academy of Sciences of Ukraine, Murmanskaya str. 1, 02660 Kiev-94, Ukraine and [3]Bayer CropScience Aktiengesellschaft BCS AG-R-WC-WCC-C2 Weed Control Chemistry 2, Frankfurt, G836, 101, Germany

Email: Yurii L. Yagupolskii - Yagupolskii@ioch.kiev.ua

*Corresponding author

NMR spectra of the most typical compounds (continuation).

(Difluoromethyl)pyrrolidin-2-ylphosphinic acid (**20e**) (Table 2, entry 5).

^{1}H (500 MHz), D_2O

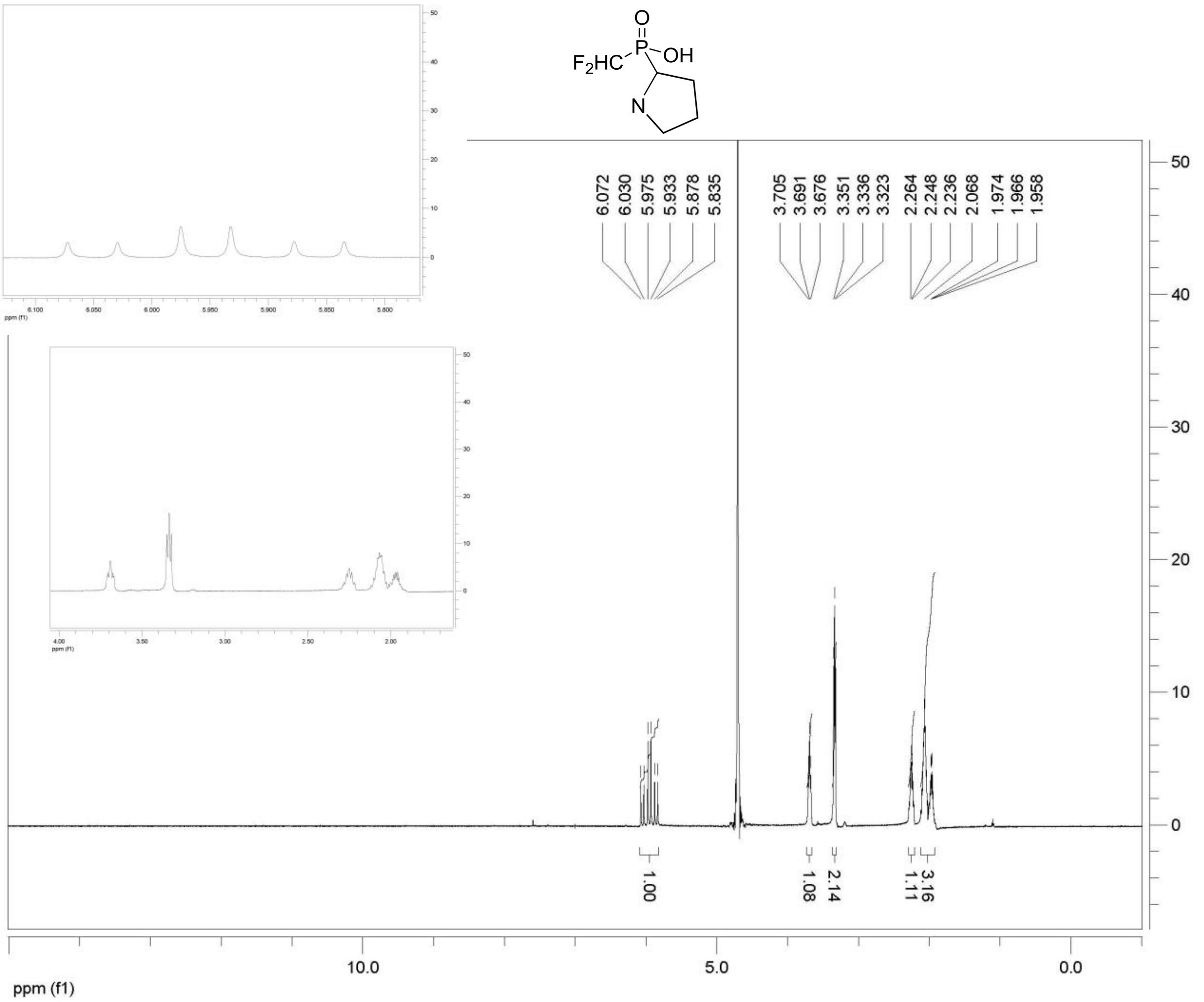

^{13}C (125 MHz), D_2O

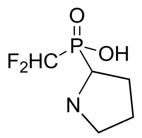

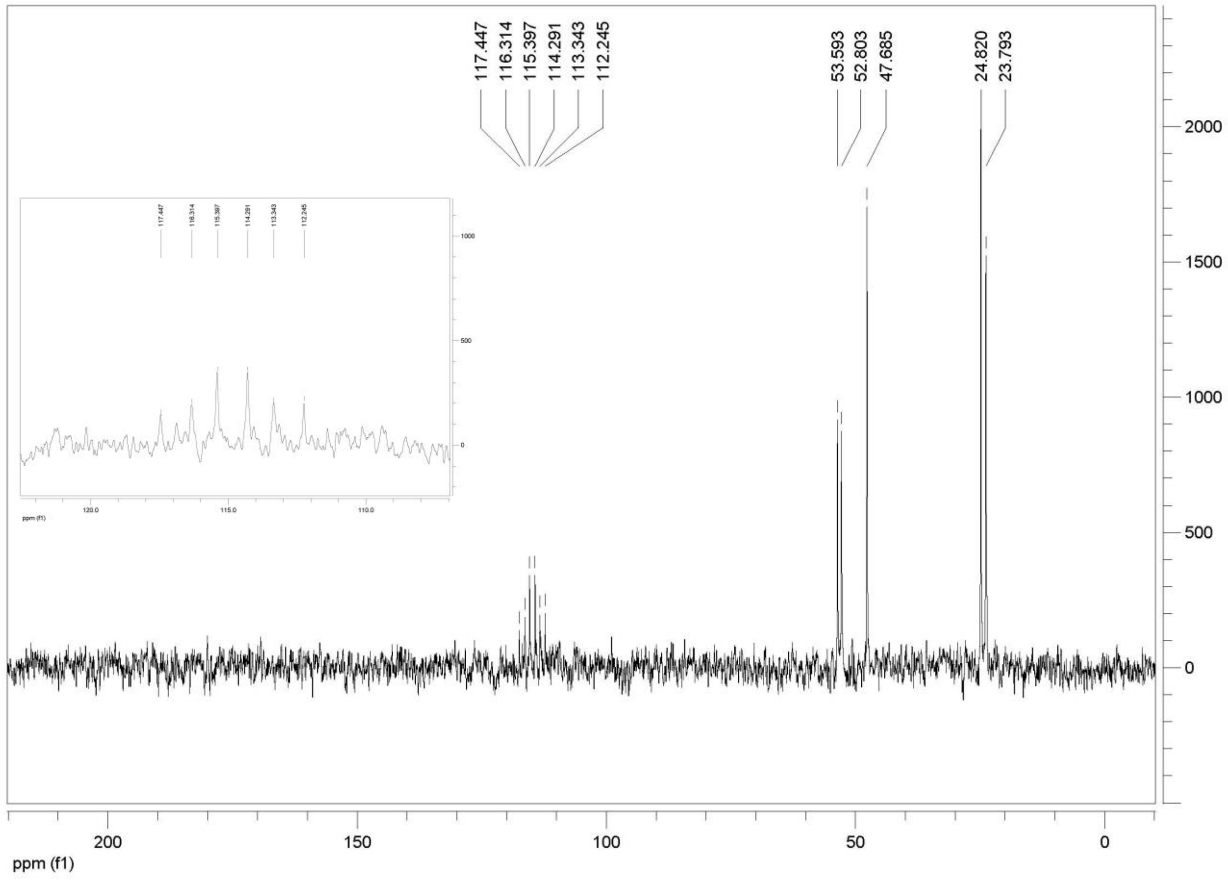

^{31}P (81 MHz), D_2O

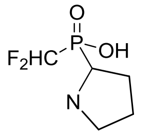

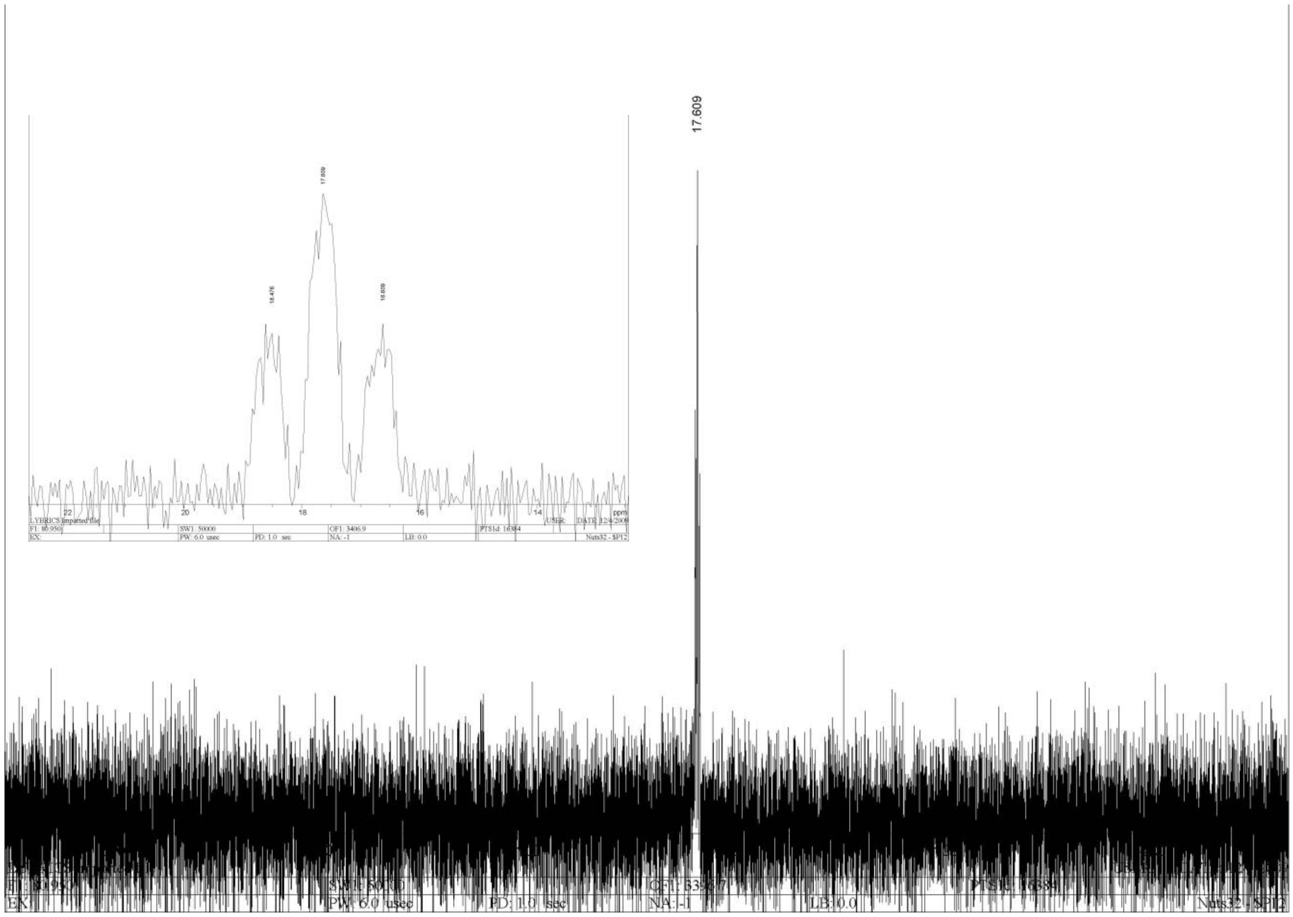

^{19}F (188 MHz), D_2O

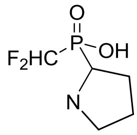

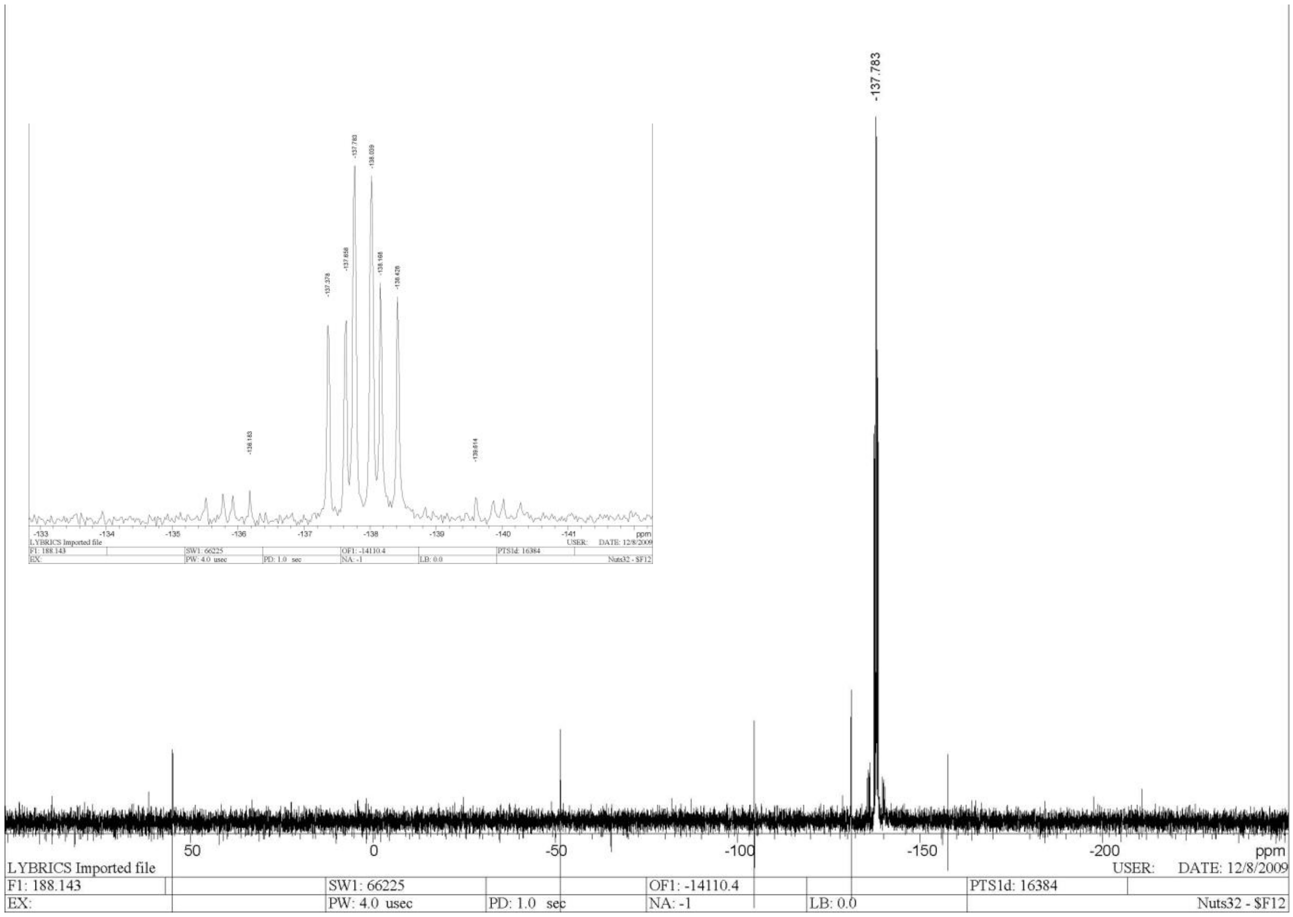

(1-Amino-2-ethoxy-2-oxoethyl)(difluoromethyl)phosphinic acid (**24**).

^{1}H (500 MHz), D_2O

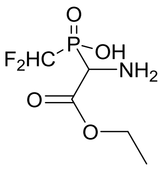

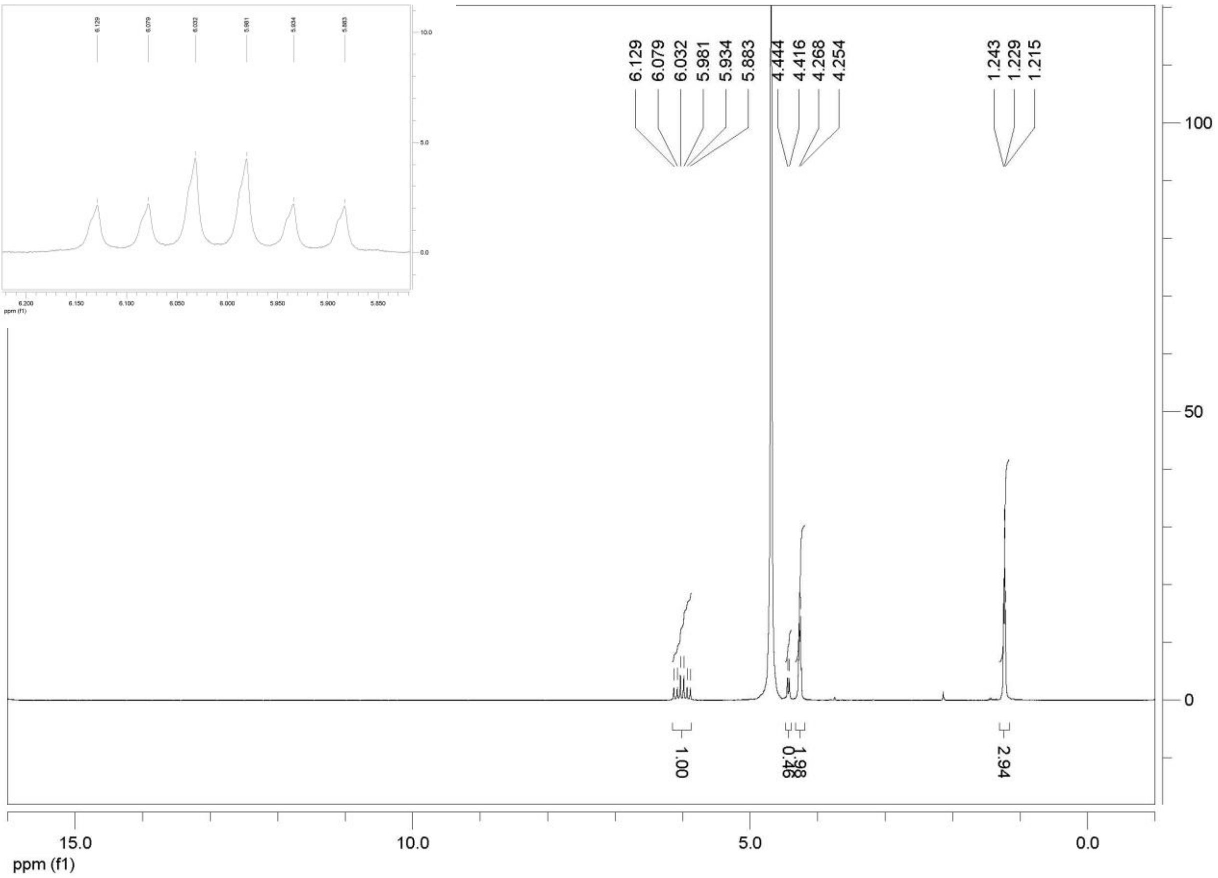

^{13}C (125 MHz), D_2O

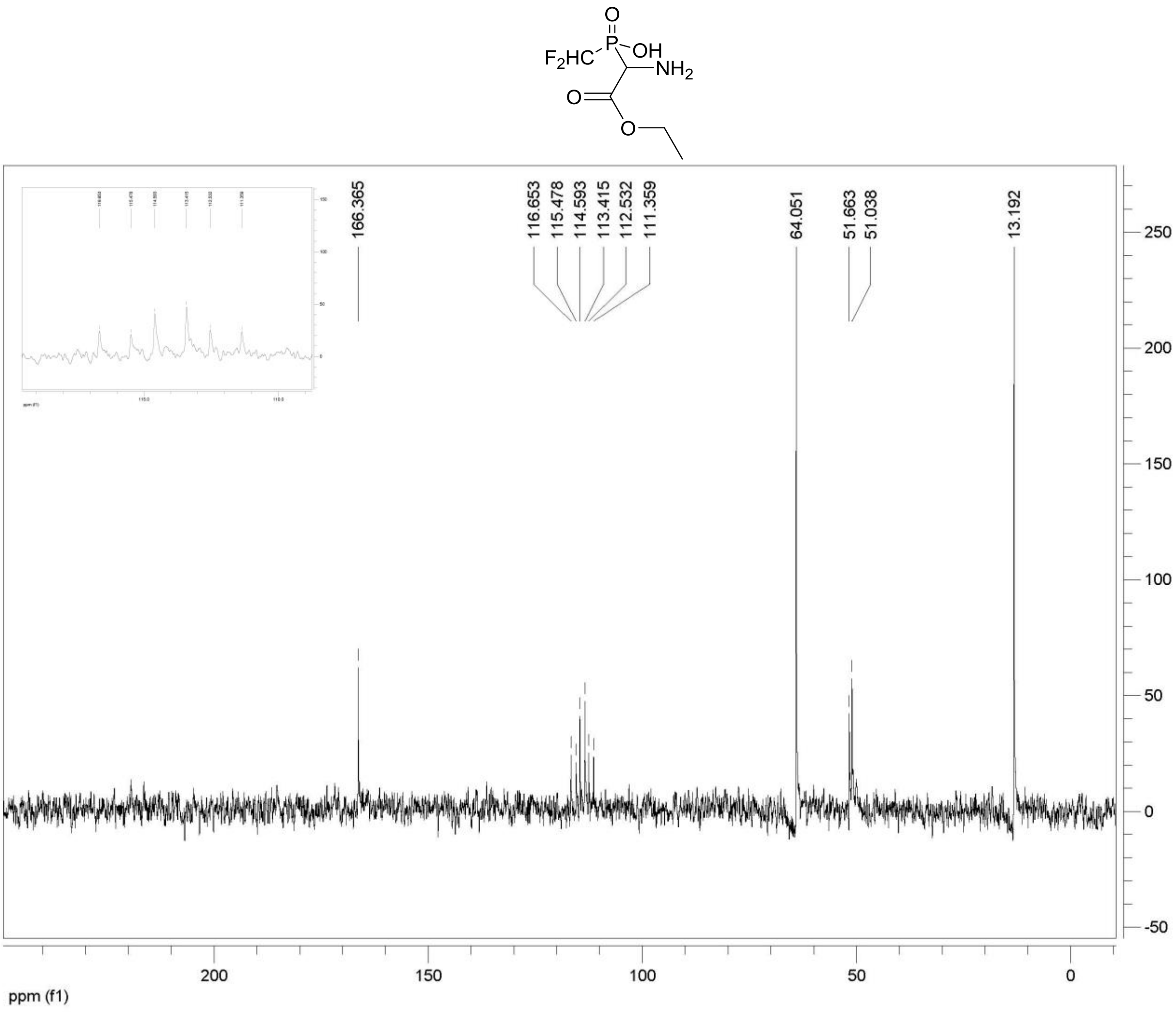

^{19}F (282 MHz), D_2O

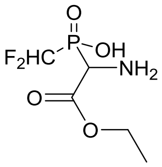

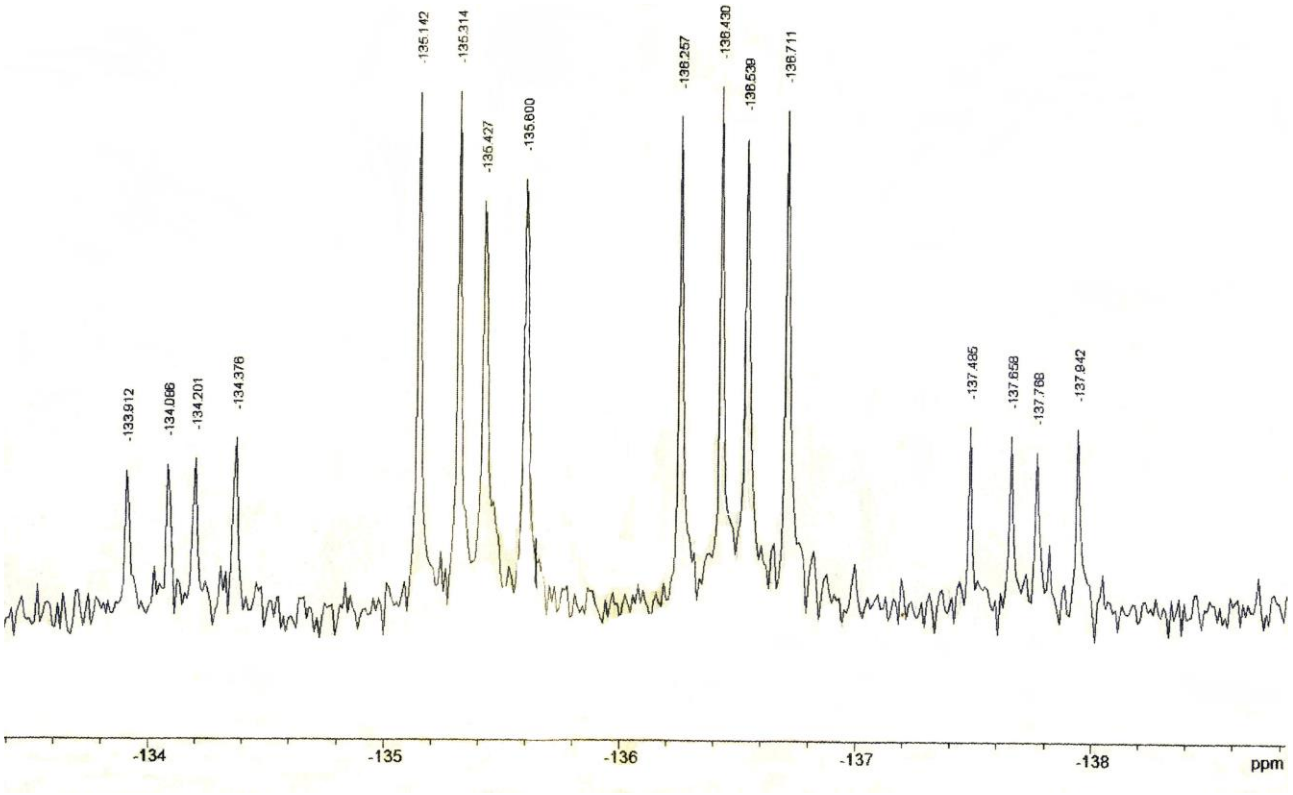

^{31}P (121 MHz), D_2O

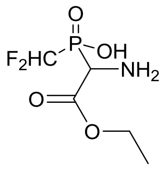

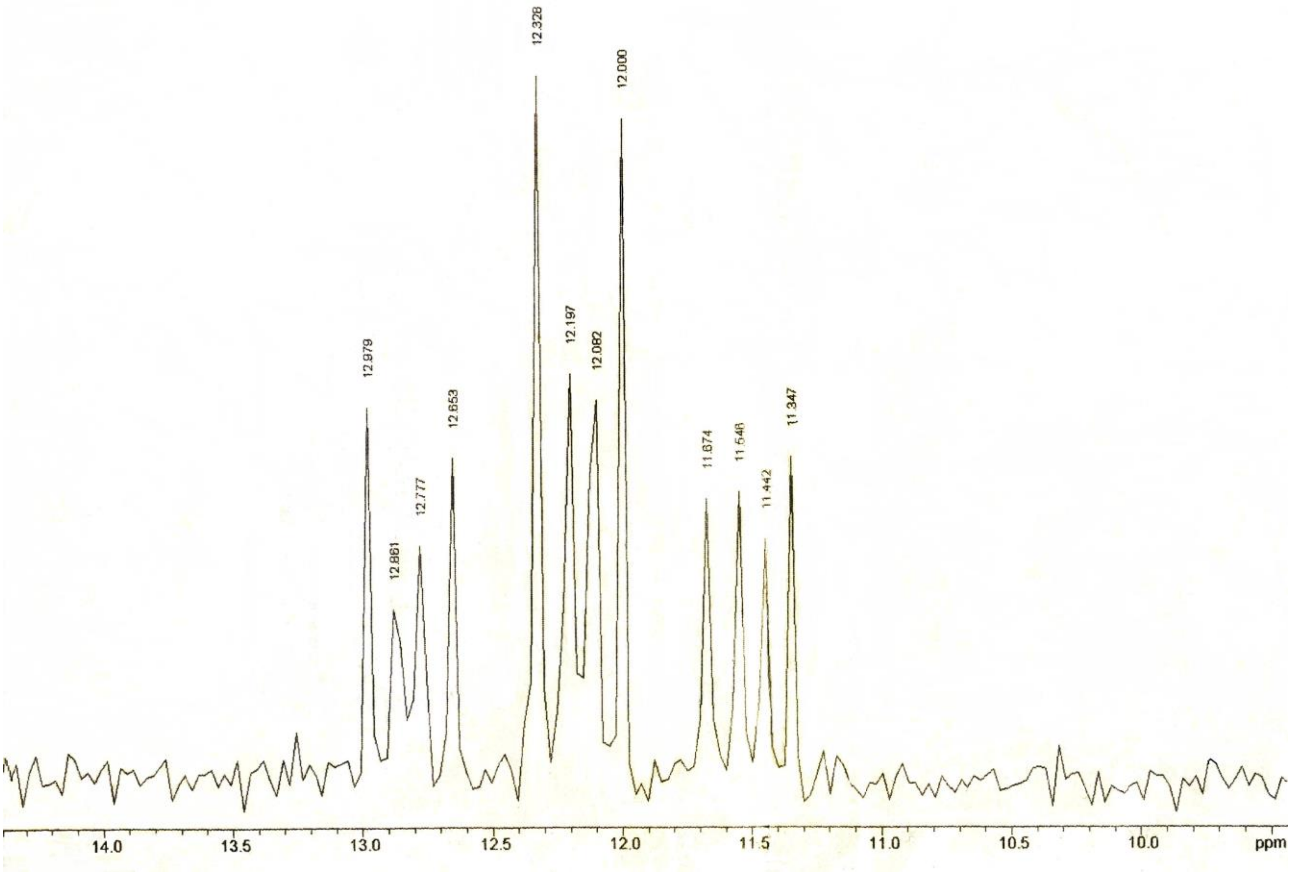

2-[Hydroxy(trifluoromethyl)phosphoryl]alanine (**32**).

^{1}H (500 MHz), D_2O

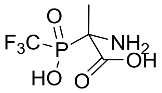

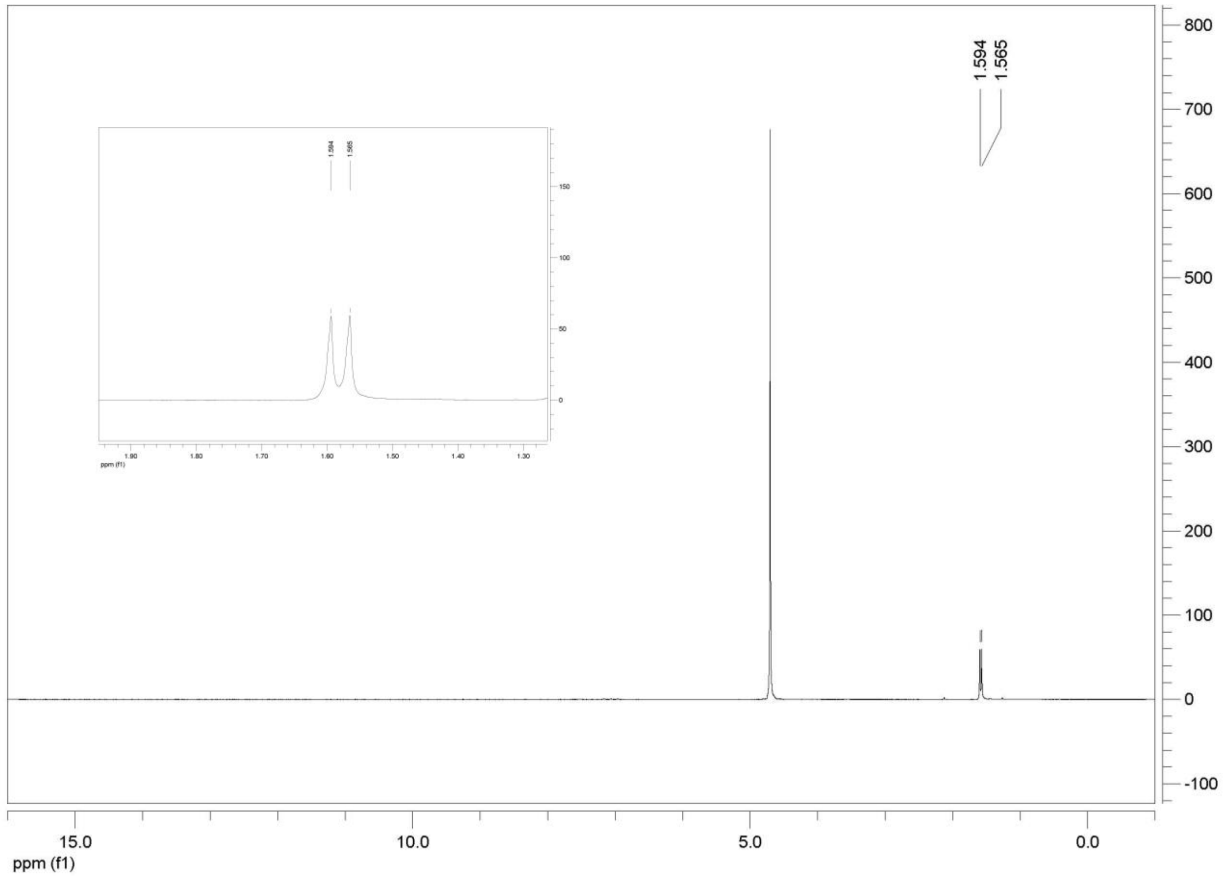

^{13}C (125 MHz), D_2O

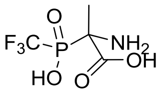

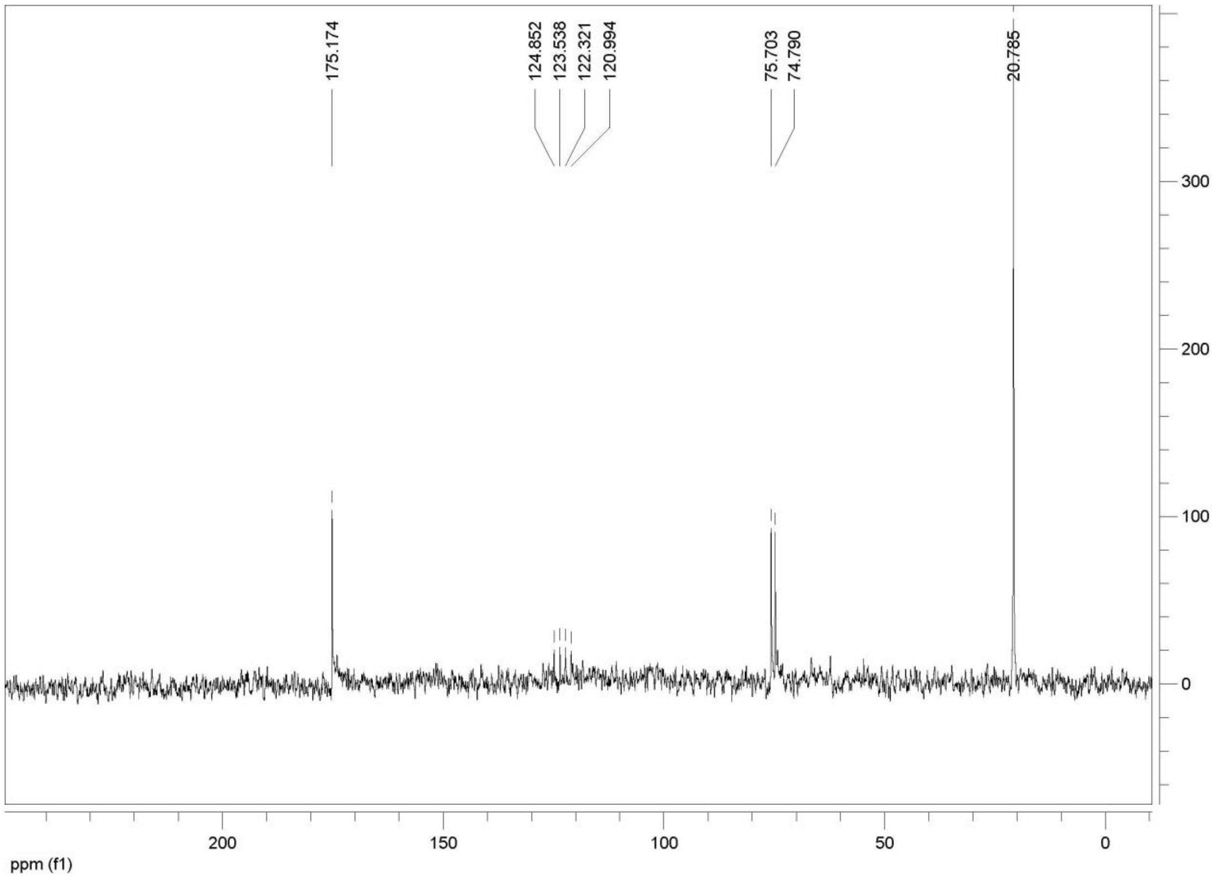

^{19}F (188 MHz), D_2O

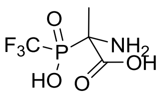

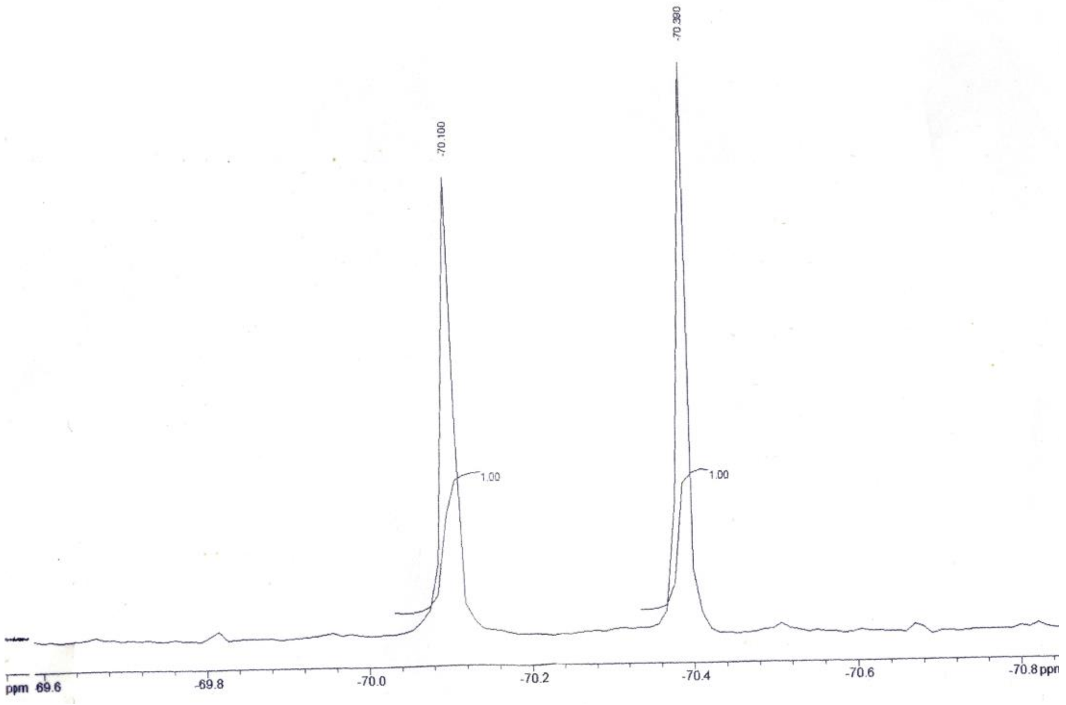

^{31}P (81 MHz), D_2O

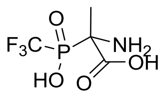

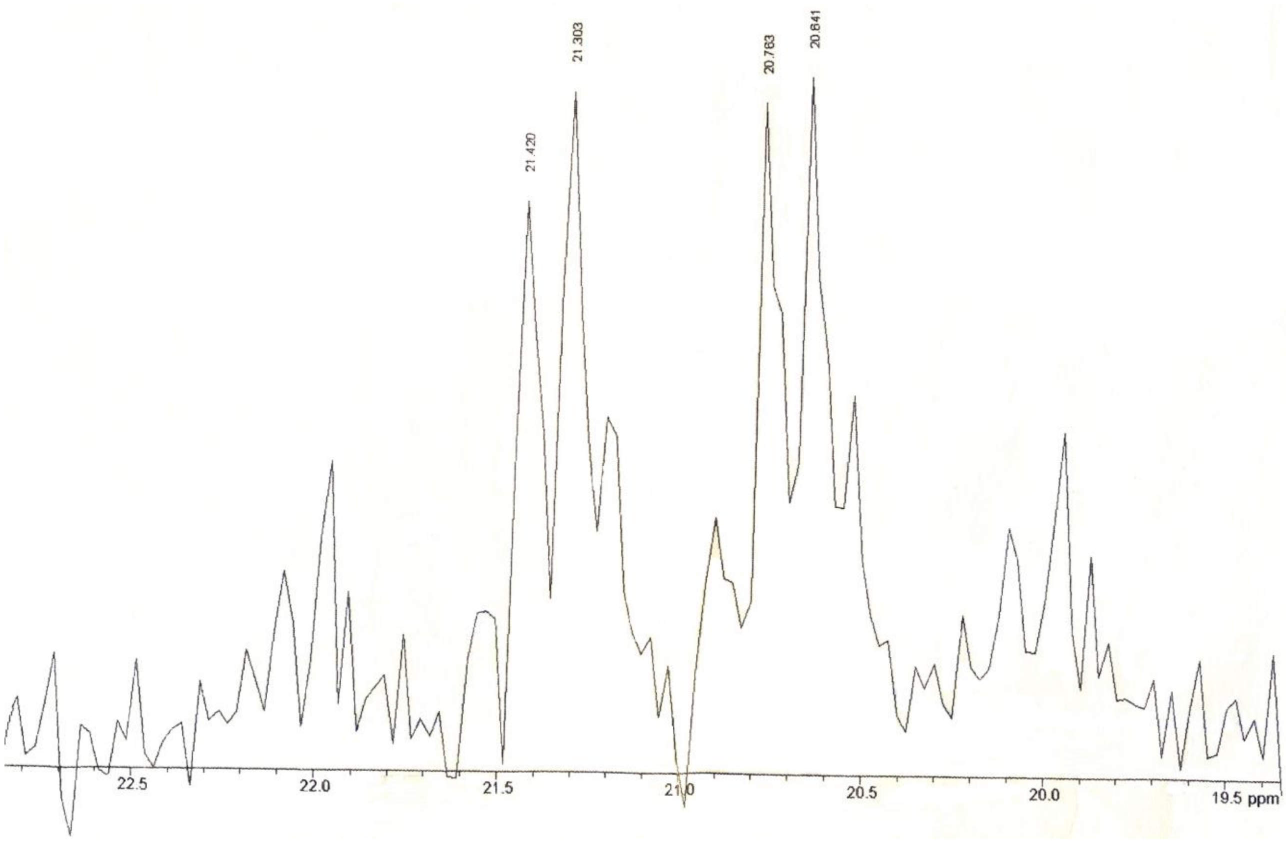

Supplement: File 3 — NMR spectra of the most typical compounds (continuation). [file Beilstein_J_Org_Chem-10-722-s003.pdf]
